# Early Home Visits and Health Outcomes in Low-Income Mothers and Offspring: 18-Year Follow-Up of a Randomized Clinical Trial

**DOI:** 10.1001/jamanetworkopen.2023.51752

**Published:** 2024-01-18

**Authors:** Gabriella Conti, Joyce Smith, Elizabeth Anson, Susan Groth, Michael Knudtson, Andrea Salvati, David Olds

**Affiliations:** 1 University College London, London, United Kingdom; 2 University of Rochester School of Nursing, Rochester, New York; 3 University of Colorado Denver–Anschutz Medical Campus, Aurora, Colorado

## Abstract

**Importance:**

Individuals with low income may have heightened rates of obesity and hypertension.

**Objective:**

To determine whether prenatal and infancy home visitation by nurses reduces maternal and offspring obesity and hypertension.

**Design, Setting, and Participants:**

This randomized clinical trial of prenatal and infancy nurse home visitation in a public health care system in Memphis, Tennessee, enrolled 742 women with no previous live births and at least 2 sociodemographic risk factors (unmarried, <12 years of education, unemployed) from June 1, 1990, through August 31, 1991. At registration during pregnancy, 727 mothers (98%) were unmarried, and 631 (85%) lived below the federal poverty level. At offspring ages 12 and 18 years, maternal and offspring obesity and hypertension were assessed by staff masked to treatment. The data analysis was performed from July 1, 2021, to October 31, 2023.

**Interventions:**

Women assigned to the control group received free transportation for prenatal care and child developmental screening and referral at child ages 6, 12, and 24 months. Women assigned to nurse visitation received transportation and screening plus prenatal and infant and toddler nurse home visits.

**Main Outcomes and Measures:**

Obesity and hypertension among mothers and their offspring at child ages 12 and 18 years, although not hypothesized in the original trial design, were analyzed using post–double selection lasso method.

**Results:**

Of the 742 participants randomized (mean [SD] age, 18.1 [3.2] years), interviews were completed with 594 mothers and 578 offspring at child age 12 years and 618 mothers and 629 offspring at child age 18 years. Obesity was assessed for 576 offspring at age 12 years and 605 at age 18 years and for 563 and 598 mothers at child ages 12 and 18 years, respectively. Blood pressure was assessed for 568 offspring aged 12 years and 596 aged 18 years and 507 and 592 mothers at child ages 12 and 18 years, respectively. There were no overall treatment-control differences in offspring obesity or hypertension at ages 12 and 18 years combined, although nurse-visited female offspring, compared with controls, had a lower prevalence of obesity (adjusted relative risk [ARR], 0.449; 95% CI, 0.234-0.858; *P* = .003) and severe obesity (ARR, 0.185; 95% CI, 0.046-0.748; *P* < .001). There were reductions at ages 12 and 18 years combined for stage 1 and stage 2 hypertension for nurse-visited vs control group mothers, with differences limited to mothers of females (stage 1: ARR, 0.613 [95% CI, 0.440-0.855; *P* = .001]; stage 2: ARR, 0.217 [95% CI, 0.081-0.582; *P* < .001]). For both obesity and hypertension outcomes, there was no intervention effect among male offspring or the mothers of males. Self-reported maternal health aligned with program effects on hypertension.

**Conclusions and Relevance:**

In this clinical trial follow-up at offspring ages 12 and 18, nurse-visited female offspring had lower rates of obesity and mothers of females had lower rates of hypertension than control-group counterparts. These findings suggest that risks for chronic disease among mothers of females and their female offspring who live in extreme poverty may be prevented with prenatal and infant and toddler home visitations by nurses.

**Trial Registration:**

ClinicalTrials.gov Identifier: NCT00708695

## Key Points

**Question:**

Does prenatal and infancy home visitation by nurses reduce obesity and hypertension among mothers with very low income and their first-born offspring at 12 and 18 years following birth?

**Findings:**

In this randomized clinical trial of 742 participants, nurse-visited female offspring had lower rates of obesity and severe obesity at age 12 and 18 years combined than their control group counterparts; nurse-visited mothers of females had lower rates of stage 1 and stage 2 hypertension at child age 12 and 18 years combined compared with their control group counterparts. No intervention effect was found for these outcomes among male offspring or mothers of males.

**Meaning:**

These findings suggest that prenatal and infancy home visitation holds promise for reducing obesity among female offspring living in extreme poverty and reducing their mothers’ hypertension.

## Teaser

This randomized clinical trial determines whether prenatal and infancy home visits by nurses reduce obesity and hypertension in mothers with low income and their offspring.

## Introduction

Poverty and adverse childhood experiences (ACEs) pose substantial risks for a range of health problems,^1,2,3,4^ including obesity among female children and adolescents^5^ and hypertensive disorders among adults.^6,7,8,9,10^ These disparities are especially pronounced among females and Black individuals.^11,12,13^ Obesity and hypertension are major public health concerns because they increase the risk for a variety of chronic health conditions, including cardiovascular disease, type 2 diabetes, and kidney failure, as well as premature mortality.^11,14,15^ Addressing these conditions among people living with very low income, and especially among Black individuals, is a public health imperative.^16^

The Nurse-Family Partnership (NFP) is a program of prenatal and infant and toddler home visiting by nurses designed to improve outcomes of (1) pregnancy, (2) child health and development, and (3) maternal health and life course (eMethods in Supplement 2).^17,18^ The NFP has been tested in a series of randomized clinical trials in the US for different populations living in different contexts, with replicated benefits found in these domains in at least 2 of these trials.^17,18^ Many program effects were most pronounced among families experiencing the greatest adversity.^18^ The focus of the current investigation is on participants in Memphis, Tennessee, living in deep poverty and in resource-poor neighborhoods.^19^ Program effects on maternal and child health in this trial have been reported for prenatal health (including pregnancy-induced hypertension),^19^ child health and development,^19,20,21,22,23^ and maternal health and life course.^19,20,21,24,25,26^

This study examines the Memphis program’s effects on maternal and offspring obesity and hypertension at offspring ages 12 and 18 years, aspects of health that were not primary or secondary outcomes in the original study design or the follow-ups at child ages 12 and 18 years.^19,22,23,25,26^ We focused on directly measured obesity (including some self-reports) and blood pressure among mothers and offspring given the relationship between obesity and hypertension and subsequent chronic disease.^11,14,15^ We examined offspring sex differences given evidence that offspring sex may moderate the association of ACEs with chronic disease.^3,4^

## Methods

For this randomized clinical trial of NFP^17,18^ in Memphis, Tennessee, recruitment was completed between June 1, 1990, and August 31, 1991.^19^ We enrolled pregnant women of less than 29 weeks gestation, with no previous live births, and with at least 2 sociodemographic risk factors (unmarried, <12 years of education, unemployed). We randomly assigned 742 women, of whom 727 (98%) were unmarried and 631 (85%) lived below the federal poverty level, to receive either free transportation for prenatal care plus child development screening and referral alone (control condition, 514 participants) or augmented with prenatal and infant and toddler home nurse visits through child age 2 years (treatment condition, 228 participants). Participants’ race and ethnicity were self-reported from a list (Aleut, Eskimo, or American Indian; Asian or Pacific Islander; Black; or White race and Hispanic or non-Hispanic ethnicity [eTable 14 in Supplement 2]). Participating mothers, other caregivers, and youths completed informed consent procedures approved by the University of Rochester institutional review board. This study followed the Consolidated Standards of Reporting Trials (CONSORT) reporting guideline.

A summary of participant baseline characteristics by treatment condition is provided in eTable 2 in Supplement 2. A description of the intervention and trial protocol is provided in Supplement 1, and the CONSORT table and analytic plan are provided in eTable 1 and the eMethods in Supplement 2. Nurses provided guidance to women on improving diet and exercise, avoiding use of toxic substances, coping with adverse conditions, monitoring blood pressure, coordinating care with primary care physicians, promoting breastfeeding and healthy feeding practices, regulating infant sleep, promoting sensitive and responsive caregiving, and encouraging opportunities for child exploration in safe environments.^17,18^ We reported previously on other maternal and child outcomes.^19,20,21,22,23,24,25,26^ In this study, we examined intervention effects on maternal and child obesity and hypertension and the moderating role of offspring sex.^3,4^

At child ages 12 and 18 years, assessed during 2003-2006 and 2008-2014, respectively, study staff measured maternal and child height and weight with shoes removed using a mechanical beam physician scale (Health O Meter 402KLS; McKesson) and blood pressure using a digital blood pressure monitor (model HEM-712C; OMRON Healthcare); in several cases (eTable 1 in Supplement 2), we accepted maternal self-report of weight and height because of factors interfering with their being weighed, including weight in excess of 350 pounds (the scale maximum), telephone interviews, and incarceration. Blood pressure measurements were repeated after 10 minutes if the initial reading exceeded normal ranges, with the last reading recorded. Elevated readings were shared with participants, who were encouraged to seek medical care. From these measurements, we constructed 4 binary outcomes: obesity, severe obesity,^27^ stage 1 hypertension, and stage 2 hypertension.^28,29^

Mothers also were asked about health conditions at the 18-year interview (eMethods in Supplement 2). To investigate the timing of treatment effects and to assist with their interpretation, we examined additional maternal and offspring health outcomes (child birth weight and gestational age and maternal blood pressure at pregnancy and birth derived from medical records). We also examined offspring weight and height (extracted from medical records) through 12 months of age and maternal reports of their child being overweight at 2 years of age (eMethods in Supplement 2). These outcomes were examined to understand their possible mediating role in explaining program effects on maternal and offspring obesity and hypertension at child ages 12 and 18 years. The main results for obesity and hypertension are reported in Table 1, Table 2, Table 3, and Table 4 and the Figure; the additional outcomes are reported in eTables 5 and 6 and eFigures 1 and 2 in Supplement 2; and the results of mediation and intergenerational analyses are presented in eTables 7 and 8 in Supplement 2. Additional supporting analyses are provided in the eMethods in Supplement 2.

**Table 1.** Postintervention Nurse-Family Partnership Effects on Offspring Obesity and Severe Obesity by Offspring Sex^a^

Outcome	No. (%)	Proportion, mean (SD)		ARR (95% CI)^b^	*P* value^c^	MHT *P* value^d^
		Control	Treatment			
**Obesity at age 12 y**						
Overall	576	0.26 (0.44)	0.23 (0.42)	0.881 (0.655-1.186)	.39	.39
Female	286 (49.8)	0.32 (0.47)	0.15 (0.36)	0.493 (0.292-0.833)	.002	.006
Male	288 (50.2)	0.21 (0.41)	0.30 (0.46)	1.454 (0.963-2.197)	.09	.15
**Obesity at age 18 y**						
Overall	605	0.25 (0.43)	0.20 (0.40)	0.784 (0.574-1.068)	.11	.23
Female	307 (50.7)	0.31 (0.47)	0.22 (0.42)	0.698 (0.455-1.073)	.08	.09
Male	297 (49.3)	0.18 (0.39)	0.18 (0.39)	1.030 (0.616-1.723)	.91	.91
**Obesity at age 12 and 18 y** ^e^						
Overall	539	0.19 (0.39)	0.14 (0.35)	0.755 (0.494-1.155)	.17	.28
Female	269 (50.0)	0.25 (0.43)	0.11 (0.31)	0.449 (0.234-0.858)	.003	.01
Male	269 (50.0)	0.14 (0.34)	0.16 (0.37)	1.248 (0.682-2.282)	.49	.60
**Severe obesity at age 12 y**						
Overall	576	0.14 (0.35)	0.12 (0.33)	0.860 (0.541-1.367)	.51	.50
Female	287 (50.0)	0.18 (0.39)	0.07 (0.25)	0.396 (0.169-0.931)	.007	.04
Male	288 (50.0)	0.10 (0.30)	0.16 (0.37)	1.510 (0.803-2.837)	.23	.38
**Severe obesity at age 18 y**						
Overall	605	0.15 (0.36)	0.11 (0.32)	0.742 (0.466-1.182)	.18	.36
Female	307 (50.8)	0.18 (0.39)	0.11 (0.31)	0.561 (0.293-1.075)	.05	.05
Male	297 (49.2)	0.12 (0.32)	0.11 (0.31)	0.928 (0.460-1.871)	.83	.84
**Severe obesity at age 12 and 18 y** ^f^						
Overall	539	0.09 (0.29)	0.07 (0.25)	0.696 (0.361-1.343)	.24	.38
Female	269 (50.0)	0.12 (0.33)	0.02 (0.16)	0.185 (0.046-0.748)	<.001	.04
Male	269 (50.0)	0.06 (0.24)	0.09 (0.29)	1.517 (0. 613-3.753)	.40	.51

Abbreviations: ARR, adjusted relative risk; MHT, multiple hypothesis testing.

^a^ One or 2 observations were dropped from some of the female-specific analyses because of missing values of control variables selected by the lasso routine.

^b^ By logistic regression.

^c^ Two-sided asymptotic *P* value for the null hypothesis that the treatment effect is 0.

^d^ Two-sided MHT *P* value using the stepdown methodology of Romano and Wolf.^30^ For MHT, we grouped outcomes by severity (obesity and severe obesity separately) and by gender (overall, females, and males separately) for a total of 6 blocks, with each block including 3 outcomes (age 12 years, age 18 years, and both age 12 and 18 years).

^e^ Offspring obesity at age 12 and 18 years is defined as a standardized body mass index (BMI) at or above the 95th percentile of the BMI-for-age distribution.^27^

^f^ Offspring severe obesity at age 12 and 18 years is defined as a standardized BMI at or above the 99th percentile of the BMI-for-age distribution.^27^

**Table 2.** Postintervention Nurse-Family Partnership Effects on Maternal Obesity and Severe Obesity by Offspring Sex^a^

Outcome	No. (%)	Proportion, mean (SD)		ARR (95% CI)^b^	*P* value^c^	MHT *P* value^d^
		Control	Treatment			
**Obesity at age 12 y**						
Overall	563	0.53 (0.50)	0.55 (0.50)	1.003 (0.877-1.148)	.96	.96
Mothers of females	280 (49.8)	0.58 (0.49)	0.54 (0.50)	0.978 (0.812-1.179)	.82	.94
Mothers of males	282 (50.2)	0.49 (0.50)	0.57 (0.50)	1.017 (0.839-1.234)	.86	.96
**Obesity at age 18 y**						
Overall	598	0.60 (0.49)	0.58 (0.49)	0.950 (0.836-1.080)	.43	.67
Mothers of females	296 (49.6)	0.62 (0.49)	0.57 (0.50)	0.939 (0.778-1.133)	.51	.76
Mothers of males	301 (50.4)	0.59 (0.49)	0.60 (0.49)	0.929 (0.778-1.110)	.41	.65
**Obesity at age 12 and 18 y** ^e^						
Overall	525	0.48 (0.50)	0.51 (0.50)	1.030 (0.882-1.202)	.71	.85
Mothers of females	259 (49.4)	0.51 (0.50)	0.50 (0.50)	1.017 (0.807-1.282)	.89	.94
Mothers of males	265 (50.6)	0.46 (0.50)	0.52 (0.50)	1.005 (0.816-1.238)	.96	.96
**Severe obesity at age 12 y**						
Overall	563	0.32 (0.47)	0.35 (0.48)	0.993 (0.815-1.210)	.94	.99
Mothers of females	280 (49.8)	0.33 (0.47)	0.36 (0.48)	1.111 (0.834-1.479)	.48	.75
Mothers of males	282 (50.2)	0.32 (0.47)	0.34 (0.48)	0.850 (0.646-1.118)	.23	.43
**Severe obesity at age 18 y**						
Overall	598	0.34 (0.48)	0.37 (0.48)	1.000 (0.828-1.207)	>.99	>.99
Mothers of females	297 (49.7)	0.38 (0.49)	0.36 (0.48)	0.941 (0.707-1.252)	.67	.84
Mothers of males	301 (50.3)	0.31 (0.47)	0.37 (0.49)	1.006 (0.788-1.283)	.96	>.99
**Severe obesity at age 12 and 18 y** ^f^						
Overall	525	0.27 (0.45)	0.31 (0.46)	1.055 (0.839-1.327)	.65	.90
Mothers of females	260 (49.5)	0.28 (0.45)	0.30 (0.46)	1.060 (0.752-1.494)	.74	.84
Mothers of males	265 (50.5)	0.26 (0.44)	0.31 (0.47)	1.001 (0.741-1.352)	>.99	>.99

Abbreviations: ARR, adjusted relative risk; MHT, multiple hypothesis testing.

^a^ One observation was dropped from some of the female-specific analyses because of missing values of a control variable selected by the lasso routine.

^b^ By logistic regression.

^c^ Two-sided asymptotic *P* value for the null hypothesis that the treatment effect is 0.

^d^ Two-sided MHT *P* value using the stepdown methodology of Romano and Wolf.^30^ For MHT, we grouped outcomes by severity (obesity and severe obesity separately) and by gender (overall, females, and males separately) for a total of 6 blocks, with each block including 3 outcomes (age 12 years, age 18 years, and both age 12 and 18 years).

^e^ Offspring obesity at age 12 and 18 years is defined as a standardized body mass index (BMI) at or above the 95th percentile of the BMI-for-age distribution.^27^

^f^ Offspring severe obesity at age 12 and 18 years is defined as a standardized BMI at or above the 99th percentile of the BMI-for-age distribution.^27^

**Table 3.** Postintervention Nurse-Family Partnership Effects on Offspring Stage 1 and Stage 2 Hypertension by Offspring Sex^a^

Outcome^b^	No. (%)	Proportion, mean (SD)		ARR (95% CI)^c^	*P* value^d^	MHT *P* value^e^
		Control	Treatment			
**Stage 1 hypertension at age 12 y**						
Overall	568	0.13 (0.33)	0.10 (0.30)	0.775 (0.457-1.316)	.32	.65
Females	284 (50.0)	0.11 (0.31)	0.07 (0.25)	0.676 (0.279-1.635)	.34	.60
Males	284 (50.0)	0.14 (0.35)	0.12 (0.33)	0.856 (0.443-1.651)	.63	.94
**Stage 1 hypertension at age 18 y**						
Overall	596	0.40 (0.49)	0.39 (0.49)	0.978 (0.788-1.214)	.84	.83
Females	305 (51.3)	0.42 (0.50)	0.39 (0.49)	0.927 (0.687-1.251)	.61	.60
Males	290 (48.7)	0.38 (0.49)	0.39 (0.49)	1.038 (0.761-1.416)	.82	.94
**Stage 1 hypertension at age 12 and 18 y**						
Overall	527	0.06 (0.24)	0.04 (0.20)	0.676 (0.288-1.590)	.33	.65
Females	264 (50.2)	0.07 (0.26)	0.04 (0.19)	0.539 (0.157-1.852)	.26	.60
Males	262 (49.8)	0.06 (0.23)	0.05 (0.22)	0.798 (0.220-2.898)	.72	.94
**Stage 2 hypertension at age 12 y**						
Overall	568	0.05 (0.22)	0.06 (0.24)	1.249 (0.609-2.561)	.56	.78
Females	284 (50.0)	0.06 (0.23)	0.06 (0.23)	1.170 (0.429-3.192)	.77	.89
Males	284 (50.0)	0.05 (0.21)	0.07 (0.25)	1.426 (0.513-3.962)	.52	.74
**Stage 2 hypertension at age 18 y**						
Overall	596	0.11 (0.32)	0.10 (0.30)	0.904 (0.551-1.480)	.68	.78
Females	306 (51.3)	0.13 (0.34)	0.12 (0.32)	0.873 (0.462-1.650)	.67	.89
Males	290 (48.7)	0.10 (0.29)	0.09 (0.29)	0.930 (0.422-2.047)	.85	.86

Abbreviations: ARR, adjusted relative risk; MHT, multiple hypothesis testing.

^a^ One observation was dropped from some of the female-specific analyses because of missing values of a control variable selected by the lasso routine.

^b^ The thresholds for determining adolescent hypertension followed US Department of Health and Human Service Guidelines.^28^ There are only 3 patients with stage 2 hypertension at both age 12 and 18 years, all females (2 in the control group and 1 in the treatment group). Given the extremely low number of cases, we chose to exclude this outcome from the analysis.

^c^ By logistic regression.

^d^ Two-sided asymptotic *P* value for the null hypothesis that the treatment effect is 0.

^e^ Two-sided MHT *P* value using the stepdown methodology of Romano and Wolf.^30^ For MHT, we grouped outcomes by stage of hypertension (stage 1 and stage 2 separately) and by gender (overall, females, and males separately) for a total of 6 blocks, with the stage-1 block including 3 outcomes (age 12 years, age 18 years, and both age 12 and 18 years) and the stage-2 block including 2 outcomes (age 12 years and age 18 years).

**Table 4.** Postintervention Nurse-Family Partnership Effects on Maternal Stage 1 and Stage 2 Hypertension by Offspring Sex^a^

Outcome^b^	No. (%)	Proportion, mean (SD)		ARR (95% CI)^c^	*P* value^d^	MHT *P* value^e^
		Control	Treatment			
**Stage 1 hypertension at age 12 y**						
Overall	507	0.59 (0.49)	0.48 (0.50)	0.815 (0.682-0.975)	.01	.03
Mothers of females	253 (50.0)	0.61 (0.49)	0.43 (0.50)	0.695 (0.523-0.925)	.01	.00
Mothers of males	253 (50.0)	0.56 (0.50)	0.52 (0.50)	0.914 (0.716-1.166)	.46	.73
**Stage 1 hypertension at age 18 y**						
Overall	592	0.81 (0.39)	0.75 (0.43)	0.927 (0.845-1.017)	.10	.07
Mothers of females	293 (49.1)	0.81 (0.39)	0.67 (0.47)	0.793 (0.674-0.934)	.003	.002
Mothers of males	298 (50.9)	0.82 (0.39)	0.83 (0.38)	1.035 (0.934-1.146)	.52	.75
**Stage 1 hypertension at age 12 and 18 y**						
Overall	486	0.54 (0.50)	0.43 (0.50)	0.791 (0.648-0.966)	.01	.03
Mothers of females	238 (49.8)	0.57 (0.50)	0.36 (0.48)	0.613 (0.440-0.855)	.001	.002
Mothers of males	247 (50.9)	0.52 (0.50)	0.49 (0.50)	0.946 (0.730-1.226)	.67	.75
**Stage 2 hypertension at age 12 y**						
Overall	507	0.30 (0.46)	0.19 (0.39)	0.634 (0.459-0.876)	.002	.01
Mothers of females	252 (49.8)	0.33 (0.47)	0.10 (0.31)	0.313 (0.156-0.631)	<.001	.002
Mothers of males	254 (50.2)	0.27 (0.45)	0.27 (0.45)	0.935 (0.636-1.374)	.73	.90
**Stage 2 hypertension at age 18 y**						
Overall	592	0.50 (0.50)	0.51 (0.50)	1.017 (0.865-1.195)	.84	.84
Mothers of females	293 (49.6)	0.51 (0.50)	0.46 (0.50)	0.864 (0.662-1.129)	.27	.25
Mothers of males	298 (50.4)	0.50 (0.50)	0.56 (0.50)	1.160 (0.938-1.435)	.18	.37
**Stage 2 hypertension at age 12 and 18 y**						
Overall	480	0.23 (0.42)	0.14 (0.35)	0.605 (0.400-0.914)	.01	.02
Mothers of females	232 (48.4)	0.27 (0.44)	0.05 (0.23)	0.217 (0.081-0.582)	<.001	.002
Mothers of males	247 (51.6)	0.20 (0.40)	0.21 (0.41)	1.076 (0.647-1.789)	.78	.90

Abbreviations: ARR, adjusted relative risk; MHT, multiple hypothesis testing.

^a^ One observation was dropped from some of the female-specific analyses because of missing values of a control variable selected by the lasso routine.

^b^ Maternal stage 1 hypertension is defined as systolic blood pressure of 130 mm Hg or greater or diastolic blood pressure 80 mm Hg or greater. Maternal stage 2 hypertension is defined as systolic blood pressure 140 mm Hg or greater or diastolic blood pressure 90 mm Hg or greater.^29^

^c^ By logistic regression.

^d^ Two-sided asymptotic *P* value for the null hypothesis that the treatment effect is 0.

^e^ Two-sided MHT *P* value using the stepdown methodology of Romano and Wolf.^30^ For MHT, we grouped outcomes by stage of hypertension (stage 1 and stage 2 separately) and by gender (overall, females, and males separately) for a total of 6 blocks, with each block including 3 outcomes (age 12 years, age 18 years, and both age 12 and 18 years).

**Figure.**
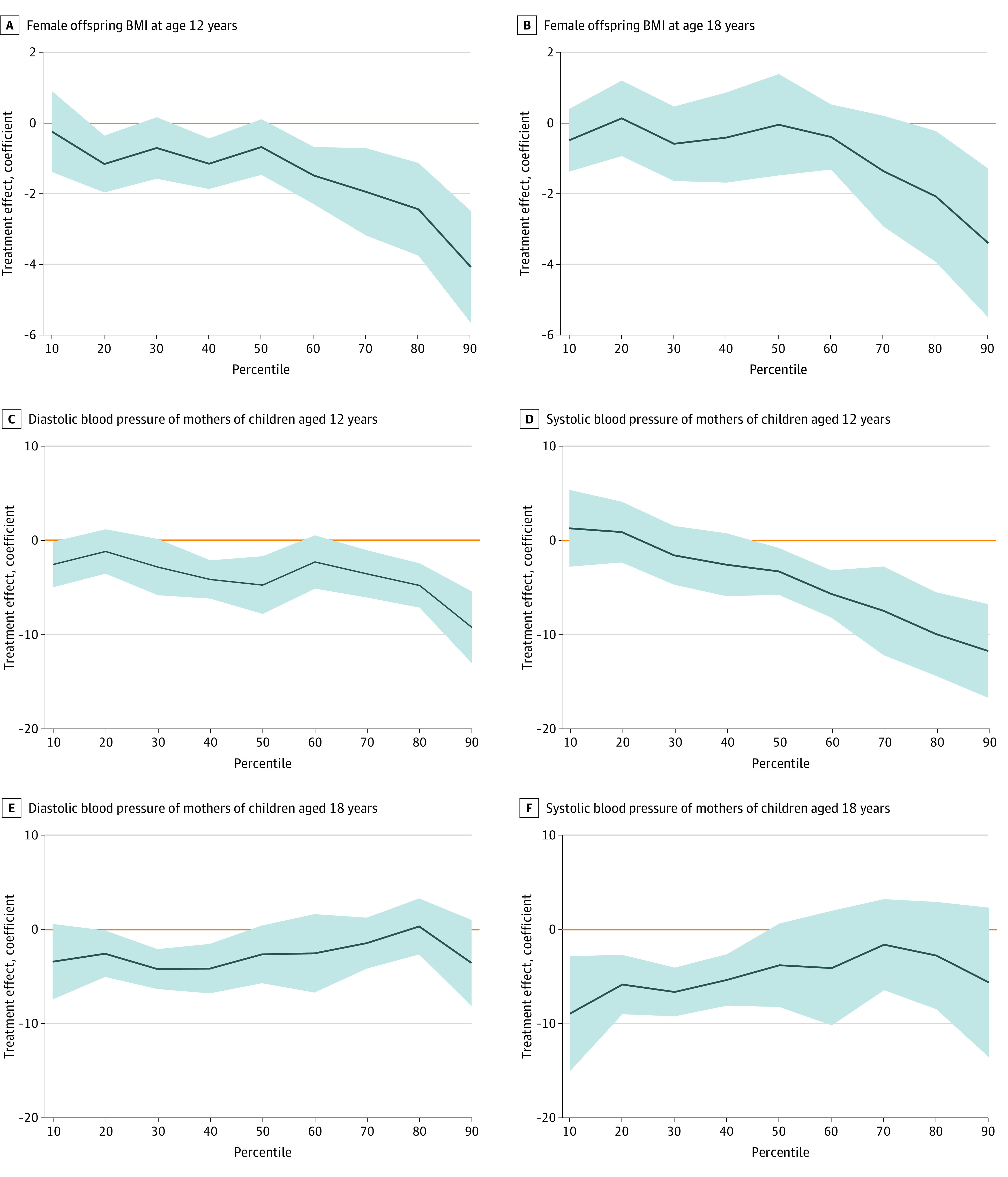
Distribution of Nurse-Family Partnership Treatment Effects on Main Outcomes, Female Offspring, and Mothers of Females Solid lines indicate coefficients and shaded areas, the 90% CIs from quantile regressions (ie, treatment effects) at different deciles of the outcome distribution. Note that the 0 values shown on the y-axes represent the points on the distributions where there are no treatment-control differences. We controlled for the variables used in the randomization protocol (race and ethnicity, age, gestational age at intake, head of household employed, and region of residence) (eTable 2 in Supplement 2, top) and additional baseline covariates selected from a set of 130 using the post–double selection lasso procedure.^31^ The quantiles were estimated simultaneously by performing 1000 bootstrap replications.

### Statistical Analysis

Given evidence of the role of sex in moderating the association of early ACEs with cardiometabolic risk,^3,4^ we analyzed NFP outcomes by child sex for both offspring and their mothers. We implemented state-of-the-art statistical methods to tackle common issues when examining long-term effects of early interventions.^32^ First, given that sex was not hypothesized as a moderator of NFP effects in the original study protocol, we examined NFP-control differences for a variety of baseline covariates. The balancing tests (eTable 2 in Supplement 2) showed few baseline differences. To account for these differences and to increase the precision of our estimates, we used the post–double selection lasso method.^31^ This method involves data-driven selection of control variables that allowed us to correct estimates for baseline imbalances, which could occur even in a randomized clinical trial. Second, given that obesity and hypertension were not included as primary or secondary outcomes in the original protocol and numerous outcomes were analyzed, we accounted for multiplicity of tests within outcome categories using a stepdown procedure known as the Romano-Wolf correction, which controls the familywise error rate.^30^ In addition, ex-post power calculations (eTable 3 in Supplement 2) showed that we had at least 80% power (α = .05) for the majority of outcomes. Third, we performed a robustness check that accounted for sample loss at follow-up (which was low and not associated with treatment status [eTables 1 and 4 in Supplement 2]) by using inverse probability weighting (eTable 9 in Supplement 2).^33,34^ For mediation analyses, we used a method developed by Gelbach^35^ that accounts for the correlation between a given set of variables hypothesized to explain treatment effects. Details of the statistical methods are reported the eMethods in Supplement 2.

The data analysis was performed between July 1, 2021, and October 31, 2023, using Stata, version 18.0 statistical software (StataCorp LLC). A 2-sided *P* < .05 was set as the threshold for significance by logistic regression and multiple hypothesis testing.

## Results

Of the 742 participants randomized (mean [SD] age, 18.1 [3.2] years), we completed interviews with 594 mothers (NFP, 187 participants; control, 407 participants) and 578 offspring (NFP, 180 participants; control, 398 participants) at child age 12 years and 618 mothers (NFP, 192 participants; control, 426 participants) and 629 offspring (NFP, 194 participants; control, 435 participants) at child age 18 years. Of the mothers interviewed at child age 12 years, 3 (0.5%) self-reported their race as Aleut, Eskimo, or American Indian; 0 as Asian or Pacific Islander; 559 (94.1%) as Black; and 32 (5.4%) as White. All 618 reported non-Hispanic ethnicity. Of the mothers interviewed at child age 18 years, 5 (0.8%) self-reported their race as Aleut, Eskimo, or American Indian; 0 as Asian or Pacific Islander; 578 (93.5%) as Black; and 35 (5.7%) as White. One mother (0.2%) reported Hispanic ethnicity, and 617 mothers (98.8%) reported non-Hispanic ethnicity. The numbers of completed physical assessments are presented in Tables 1-4 and eTable 1 in Supplement 2. We first present findings for obesity and hypertension outcomes at child ages 12 and 18 years.

### Obesity

Obesity was assessed for 576 offspring at age 12 years and 605 at age 18 years and for 563 and 598 mothers at child ages 12 and 18 years, respectively. Table 1 presents control and NFP group differences with regard to offspring obesity and severe obesity stratified by sex and child ages 12 and 18 years. Nurse-visited female offspring were significantly less likely to have obesity and severe obesity at both ages when considering each time point in isolation and together. For example, in the control group, a mean (SD) proportion of 0.18 (0.39) at age 12 and 18 years had severe obesity, and the intervention reduced the risk of obesity by more than half at age 12 years (adjusted relative risk [ARR], 0.396; 95% CI, 0.169-0.931; *P *=* *.007) and more than 40% at age 18 years (ARR, 0.561; 95% CI, 0.293-1.075; *P *=* *.05). In contrast, among female offspring who completed interviews at both ages, a mean (SD) proportion of 0.25 (0.43) had obesity and 0.12 (0.33) had severe obesity at both ages, with NFP reducing the female offspring risk of obesity by 55% compared with the control group (ARR, 0.449; 95% CI, 0.234-0.858; *P* = .003) and the risk of severe obesity by 81% (ARR, 0.185; 95% CI, 0.046-0.748; *P* < .001). These effects were robust to accounting for multiple hypothesis testing (in the last column), attrition (eTable 9 in Supplement 2), controlling directly for imbalanced baseline covariates (eTable 10 in Supplement 2) and removing self-reported heights and weights (eTables 11 and 12 in Supplement 2). These results were confirmed by quantile regression analysis, which showed that the effects of NFP on female body mass index were most pronounced at the upper deciles of the outcome distribution (Figure, A and B). Section C of the eMethods in Supplement 2 discusses the quantile regression method used in this analysis. eTable 9 in Supplement 2 (second to last column) shows that female offspring in the control group had higher rates of obesity than male offspring at ages 12 and 18 years. For example, the mean (SD) proportion of females and males with obesity at age 12 years was 0.32 (0.47) and 0.21 (0.41), respectively. Moreover, the interactions between sex and treatment were significant or near significance for one-half of the offspring obesity outcomes (eTable 9 in Supplement 2, last column). Table 1 shows that NFP had no effect on male offspring obesity, and Table 2 shows no NFP effects on obesity or severe obesity among mothers of either male or female offspring.

### Hypertension

Blood pressure was assessed for 568 offspring at age 12 years and 596 at age 18 years (Table 3) and for 507 and 592 mothers at child ages 12 and 18 years, respectively (Table 4). Table 3 presents results for control and NFP group differences with regard to offspring stage 1 and stage 2 hypertension stratified by sex and child age 12 and 18 years. No differences were significant. Table 4 shows the corresponding outcomes for mothers. Nurse-visited mothers were significantly less likely to have stage 1 and stage 2 hypertension at child ages 12 and 18 years combined, with differences limited to mothers of females. For example, of mothers of female offspring in the control group, a mean (SD) proportion of 0.57 (0.50) had stage 1 hypertension and 0.27 (0.44) had stage 2 hypertension, with NFP reducing the risk for stage 1 hypertension by 39% (ARR, 0.613; 95% CI, 0.440-0.855; *P* = .001) and the risk for stage 2 hypertension by 79% (ARR, 0.217; 95% CI, 0.081-0.582; *P* < .001). These effects were robust to accounting for multiple hypothesis testing (in the last column) and attrition (eTable 9 in Supplement 2) and controlling for imbalanced baseline covariates (eTable 10 in Supplement 2). These results were also confirmed by quantile regression analysis, which showed that the effects of NFP on blood pressure for mothers of female offspring were most pronounced in the upper deciles of the distribution at child age 12 years (Figure, C and D) and more pronounced in the lower and middle range of the distribution at child age 18 years (Figure, E and F). There were no NFP-control group differences in hypertension among mothers of males or among male offspring.

### Other Offspring and Maternal Outcomes

#### Length of Gestation and Child Weight

Nurse-visited male offspring had longer mean gestations than control group male offspring (least squares mean difference [LSMD], 0.76 weeks; 95% CI, 0.150-1.359 weeks; *P* = .02), as well as higher birth weights (LSMD, 191.37 g; 95% CI, 55.01-327.75 g; *P* = .006). Moreover, the prevalence of preterm birth was higher among controls than the NFP group (ARR, 0.663; 95% CI, 0.455-0.965; *P* = .02), a difference more pronounced among males (eTable 5 in Supplement 2); however, this difference was not robust to accounting for multiple hypothesis testing.

#### Pregnancy-Induced Hypertension, Blood Pressure at Birth, and Maternal Health

Nurse-visited mothers had a lower mean arterial blood pressure at birth than control group mothers (LSMD, −2.226 mm Hg; 95% CI, −4.091 to −0.362; *P* = .02) and lower prevalence of any health condition at child age 18 years (12% reduction; ARR, 0.879; 95% CI, 0.775-0.997; *P* = .04) (eTable 6 in Supplement 2). These differences were concentrated in mothers of females (mean arterial blood pressure: LSMD, −4.064 mm Hg [95% CI, −6.727 to −1.401; *P* = .003]; any health condition: ARR, 0.721 [95% CI, 0.588-0.884; *P* = .001]). eTable 6 in Supplement 2 also shows that nurse-visited mothers of females reported fewer counts of health conditions at offspring age 18 years than their control group counterparts (LSMD, −0.553; 95% CI, −0.966 to −0.140; *P* = .009). No intervention effect was detected for any of these outcomes among mothers of males.

### Offspring Growth in the First Year of Life

eFigure 1 in Supplement 2 shows estimated offspring weight for age (measured during well-child visits) in the first year of life based on a growth model of the treatment effect on developmental trajectory (coefficients shown in eTable 13 in Supplement 2). Control female offspring had a significantly steeper growth profile for weight in the first year of life than nurse-visited females, peaking at age 4 through 6 months (eFigure 1A in Supplement 2). Nurse-visited female offspring showed a flatter weight pattern, centered at the median of the reference population.^27^ No treatment effects were detected among male offspring (eFigure 1B in Supplement 2) or for height for age (eFigure 2 in Supplement 2).

### Mediation Analyses

A simple mediation analysis (eTable 7 in Supplement 2) suggested that 27.3% of the NFP effect for stage 1 hypertension in mothers of females at child age 18 years was mediated by a reduction in stage 1 hypertension at child age 12 years; this increased to 30.3% when we also included maternal obesity at child age 12 years. Additionally, 75.4% of the NFP effect on female offspring obesity at age 18 years was mediated by a reduction in obesity at age 12 years; this increased to 82.6% when we also included overweight at 2 years of age. We found similar results for female offspring with severe obesity.

### Intergenerational Mobility

Simple analyses of intergenerational persistence in health are presented in eTable 8 in Supplement 2, which shows estimated regression coefficients of female offspring outcomes (eg, offspring obesity at age 18 years) on the same maternal outcome at the corresponding age (eg, maternal obesity among mothers of females at child age 18 years). The results show that NFP broke the link between mother and offspring stage 2 hypertension among female offspring and mothers of females at age 18 years, while in the control group, having a mother with severe hypertension was associated with a higher chance (11.3 correlation coefficient) of having a female child with severe hypertension. This association was absent in the treated group, with a significant 0.188 treatment difference in the intergenerational correlation coefficients.

## Discussion

In this randomized clinical trial, we found that NFP-visited female offspring compared with their control group counterparts had significantly lower rates of obesity and severe obesity at ages 12 and 18 years and that NFP-visited mothers of female offspring had significantly lower rates of stage 1 and 2 hypertension at child ages 12 and 18 years. Nurse-visited mothers overall also had fewer self-reported health conditions at child age 18 years. Of note, the program effect on stage 2 hypertension observed among mothers at child age 12 years was not significant at child age 18 years, which may be due to research staff advising mothers with elevated blood pressure at child age 12 years to seek treatment. We also found that health improvements at child age 12 years were significant mediators of the sustained treatment effects at child age 18 years.

Health benefits for female offspring in this study were already evident during the first 2 years of life, with more normal patterns of postnatal weight gain in NFP-visited female offspring, consistent with the large body of evidence showing that rapid postnatal growth may increase the risk of childhood obesity.^36^ In addition, health benefits for NFP-visited mothers of girls were already present during pregnancy, with lower rates of pregnancy-induced hypertension and lower blood pressure during labor and delivery, consistent with evidence finding an increased risk of cardiovascular disease for women who experienced hypertensive disorders in pregnancy.^37,38^ A novel analysis of intergenerational health correlations found that NFP broke the link between persistence in stage 2 hypertension between mothers and daughters. These findings are consistent with evidence on the role of early ACEs and sex differences for developing risks for chronic disease.^3,4^

Sex differences in stress response begin in pregnancy. Male embryos are more vulnerable to fetal loss.^39,40,41^ However, scarring effects of significant stressors are visible in surviving female newborns, with higher rates of preterm delivery,^42^ low birth weight,^43^ and androgen activity than male fetuses.^44^ Some studies have found that pregnant women, especially primiparous, with female fetuses may have a heightened risk for pregnancy-induced hypertension and preeclampsia,^45^ but the role of fetal sex in predicting hypertensive disorders of pregnancy is far from clear.^46,47,48,49^ Hypertensive disorders of pregnancy are unique risks for subsequent cardiovascular disease among mothers^37,38,50,51^ and their offspring.^52^ Obesity in adults, moreover, is more prevalent among females who experienced early social disadvantage.^53,54,55^ The absence of NFP effects on obesity among male offspring may be attributed to their lower rate of obesity compared with control group female offspring in our study.

Intervention effect sizes found in this study compare favorably with those uncovered in other public health early interventions among individuals with economic and social disadvantages.^56,57,58,59,60,61,62^ Follow-up of the current study at offspring age 30 years is under way to assess whether these health effects endure.^63^

### Strengths and Limitations

Our study has several key strengths. First, to our knowledge, this study is the first and longest follow-up of a randomized evaluation of a home visiting program to find persistent health effects for 2 generations and an improvement in intergenerational health mobility. While improvements in child health have been found in some contemporaneous home visiting programs,^61,62^ the extent to which benefits for mothers persist has been mostly unknown. Second, in contrast to work relying on self-reports,^56^ with a few exceptions, we used objective biomarker measurements. Third, in contrast to evaluations with high rates of attrition,^32^ we have high rates of sample retention.

This study also has some limitations. First, program effects on obesity and hypertension and moderation by sex of offspring were not hypothesized. Second, the diagnosis of hypertension was based on measures taken during only 1 visit. Third, we cannot guarantee that participants did not disclose their treatment assignment to those conducting assessments. Fourth, there is no information on medications or contemporaneous health behaviors that could affect obesity and hypertension. Fifth, the precise mechanisms through which these long-term effects were produced are complex and remain to be determined.^64^

## Conclusions

The findings of this randomized clinical trial support the importance of intervening in the earliest days of life and emphasize the importance of examining intervention effects by sex. Considering the large costs of obesity^65^ and hypertension,^66^ these results suggest that NFP can make cost-saving contributions to the physical health of individuals coping with adversity. These potential cost savings could augment those already calculated.^67^

## Notes

Supplement 1.
Trial Protocol

Supplement 2.
eMethods.
**eTable 1.** CONSORT Information for Mothers and Children Enrolled in the Trial Through the 18-Year Follow-Up **eTable 2.** Balancing Tests, Baseline Covariate **eTable 3.** Ex-Post Power Calculations, Main Outcome **eTable 4.** NFP Treatment Effects on Sample Retention **eTable 5.** NFP Effects on Length of Gestation, Child Birthweight, Weight at Age 2 Years **eTable 6.** NFP Effects on Maternal Blood Pressure at Birth, Pregnancy-Induced Hypertension, and Health Conditions at Child Age 18 Years **eTable 7.** Mediation Analysis, Mothers of Girls Hypertension and Obesity of Girls at Age 18 Years **eTable 8.** Intergenerational Correlations in Health **eTable 9.** NFP Postintervention Treatment Effects on Offspring Obesity and Severe Obesity and Maternal Stage 1 and Stage 2 Hypertension—Inverse Probability Weighting and Test for Significance of Sex Differences **eTable 10.** NFP Postintervention Treatment Effects on Child Obesity and Mother Hypertension—Robustness to Inclusion of Covariates Unbalanced at Baseline **eTable 11.** Postintervention NFP Effects on Offspring Obesity and Severe Obesity by Offspring Gender—Robustness to Exclusion of Self-Reported BMI Values **eTable 12.** Postintervention NFP Effects on Maternal Obesity and Severe Obesity by Offspring Gender—Robustness to Exclusion of Self-Reported BMI Values **eTable 13.** NFP Treatment Effects on Weight-for-Age and Height-for-Age in the First Year of Life **eTable 14.** Self-Reported Race and Ethnicity by Treatment Status **eFigure 1.** NFP Treatment Effects on Weight-for-Age in the First Year of Life **eFigure 2.** NFP Treatment Effects on Height-for-Age in the First Year of Life
eReferences

Supplement 3.
Data Sharing Statement
