# Supplementary material for: Early Home Visits and Health Outcomes in Low-Income Mothers and Offspring: 18-Year Follow-Up of a Randomized Clinical Trial
Source: JAMA Netw Open. 2024 Jan 18;7(1):e2351752. doi: 10.1001/jamanetworkopen.2023.51752 (PMC10797459; doi:10.1001/jamanetworkopen.2023.51752)
Supplement: Supplement 1. — Trial Protocol [file jamanetwopen-e2351752-s001.pdf]

# INTRODUCTION

The protocol specified in the following pages was funded by NIH (NIDA) in 2008 to support the 18-year follow-up of participants in a randomized trial of Nurse-Family Partnership conducted in Memphis TN. This trial formed the foundation for a subsequent study of obesity and hypertension among mothers and offspring. These two outcomes were not hypothesized in the original protocol. The focus on obesity and hypertension moves this study into a longitudinal cohort study that exploits the advantages of randomization, that is, reducing intervention-control baseline differences. We have removed our response to earlier reviewers' comments in the protocol that follows.

## A. SPECIFIC AIMS

This study is a longitudinal follow-up of 670 primarily African-American women and their 17-year-old firstborn children enrolled since 1990 in a highly significant randomized controlled trial (RCT) of prenatal and infancy home visiting by nurses. Nurses in this program are charged with improving pregnancy outcomes, child health and development, and maternal economic self-sufficiency. This follow-up examines whether earlier program effects on maternal and child functioning lead to less violent antisocial behavior, psychopathology, substance use and use-disorders, and risk for HIV; whether these effects are greater for those at both genetic and environmental risk; and whether program effects replicate those found with whites in an earlier trial.[5;6] Results to date show that the Memphis program affected women's prenatal health, fertility, partner relations, and use of welfare; children's injuries, cognition, language, achievement, conduct, depression/anxiety, and use of substances through child age 12. Program effects on maternal life-course were concentrated among mothers with higher psychological resources (better intellectual functioning, mental health, and sense of mastery), probably because higher-resource mothers could envision their success in the world of work, leading to better pregnancy planning and employment. Program effects on children were greater for those born to mothers with low psychological resources, because without help, low-resource mothers are especially challenged in the care of their children and their children function less well. Given the damaging effects of early stressors on developing neural circuitry, and given that many early neural developmental insults do not become fully evident until synaptic pruning is complete in late adolescence and early adulthood, there is reason to expect this early intervention will have enduring effects.

**Hypotheses**

We have specified hypotheses based upon the pattern of results found to date and have separated them into primary and secondary hypotheses. Compared to control-group counterparts:

**Maternal Outcomes**

1. **(Primary)** The program will continue to improve maternal life-course (fewer short inter-birth intervals, less use of welfare, more stable partner relations), especially for mothers with higher psychological resources.
2. **(Secondary)** The program will reduce maternal substance use disorders (SUDs) and depression, effects that will be more pronounced for a) mothers with low psychological resources, and b) those living in the most disadvantaged neighborhoods at registration.

**Child Outcomes**

3. **(Primary)** The program will improve the health and development of firstborn children who will exhibit: a) superior cognitive, language, and academic functioning, and executive cognitive functioning (ECF); b) less depression and anxiety; c) fewer failed conduct grades and school disciplinary actions, d) less violent behavior and gang membership, and fewer arrests, juvenile detentions, and convictions - especially for crimes involving interpersonal violence.
4. **(Primary)** The program will reduce children's risk for HIV infection, including a) use of substances and SUDs; b) risky sexual behaviors; c) sexually transmitted infections (STIs) and d) pregnancies.
5. **(Primary)** Program effects on children will be more pronounced for a) males, b) those born to low-resource mothers, and c) those living in the most disadvantaged neighborhoods at registration.

**Maternal and Child Outcomes**

6. **(Secondary)** Program effects on mothers and children, in preliminary analyses, will be more pronounced for those with genetic vulnerabilities:
   a. Effects on youth depression and anxiety will be greater for those with low-activity genotypes (S/S, $L_G/L_G$, S/$L_G$) of the serotonin transporter gene (*SLC6A4*) promoter polymorphism, 5-HTTLPR, compared to those with high-activity genotypes ($L_A/L_A$); effects on these outcomes will be of intermediate magnitude for those with intermediate activity-level genotypes (S/$L_A$, $L_A/L_G$).
   b. Effects on youth violent antisocial behavior, SUDs, and risky sexual behavior will be more pronounced among males with the MAOA-LPR low activity alleles compared to males with MAOA-LPR high activity alleles, and among both males and females with 2 copies of the high-activity Val allele of the COMT Val158Met polymorphism compared to those with 2 copies of the low-activity met allele or heterozygotes.
   c. Effects on maternal SUDs will be concentrated among mothers with 2 copies of the Val158 alleles.
   d. Effects on child outcomes will be more pronounced among children born to mothers with either 1) the S/S, S/$L_G$ and "$L_G/L_G$" (low-activity) genotypes of 5-HTTLPR (conferring risk for depression under adversity) or 2) 2 copies of the high activity COMT Val158 allele (conferring risk compromised ECF and SUDs).
7. **(Secondary)** Program effects on adolescent functioning will be explained by its improvement in prenatal health, early care of the child, maternal life-course, and earlier child academic and behavioral functioning.

## B. BACKGROUND AND SIGNIFICANCE

The Nurse-Family Partnership (NFP), the program examined in this trial, is different from most substance-abuse prevention efforts examined to date in that it focuses on improving early neuro-developmental, cognitive, and behavioral functioning of the child by improving prenatal health, reducing child abuse and neglect, and enhancing family economic self-sufficiency in the first two years of the child's life. These early alterations in biology, behavior, and family context are expected to shift the life-course trajectories of children living in highly disadvantaged families and neighborhoods away from psychopathology, SUDs, and risky sexual behaviors—all risks for HIV infection. Most substance-abuse preventive interventions have focused on school-age children in the pre-adolescent or adolescent age range, but Fishbein suggests that neuro-cognitive and socio-emotional risks rooted in early experience and biology can undermine the extent to which minority youth make use of conventional substance-use prevention efforts.[7]

Noting that adolescent SUDs are associated with childhood psychopathology and that treatment of childhood psychopathology can reduce subsequent SUDs, Kendall and Kessler[8] have recommended earlier treatment of childhood mental disorders. They question the value of preventive interventions on the grounds that many who need such interventions fail to participate because they have no sense of vulnerability to motivate participation. Low-income pregnant women bearing first babies, the target population served by the NFP, have profound senses of vulnerability during this specific time in their lives, however, that probably contributes to the high rates of participation in this prevention program.[9] Moreover, the program is now being replicated in hundreds of communities throughout the US.[10] Much of the policy impetus for the program, however, has been generated by the results of the Elmira trial (with a primarily white sample) on adolescent functioning through child age 15.[11] Replication of the Elmira findings, especially with different populations, is crucial. It also is important to gain a deeper understanding of its impact on adolescent SUDs, risk for HIV, psychopathology, and violence, and an understanding of those groups for whom the program does and does not work. In evaluating this proposal, it is important to appreciate the program's conceptual foundations.[9;12;13]

### Epidemiologic and Developmental Foundations

***Focus on Low-Income, Unmarried, and Teen Parents.*** The NFP registers low-income women having first births, and thus enrolls large portions of unmarried and adolescent mothers. These populations have higher rates of the problems the program was designed originally to address (e.g., poor birth outcomes, child abuse and neglect, and diminished parental economic self-sufficiency).[14;15] Women bearing first children are particularly receptive to this service, and to the extent that they improve their prenatal health, care of their firstborns, and life-course they are likely to apply those skills to subsequent children they choose to have.[9;16]

***Program Content.*** Figure 1 shows the general conceptual model that has guided the nurses' work and how the program is thought to affect outcomes and moderate genetic vulnerabilities in the presence of stress. It is important to note that we assume that the program is operating in the context of high rates of neighborhood disadvantage and family poverty and stress, which are not shown explicitly in this figure. Moreover, given low rates of prenatal tobacco use in the Memphis sample. functional polymorphisms in GSTTI and DAT are not discussed in this proposal, even though they have been shown to intact with prenatal tobacco

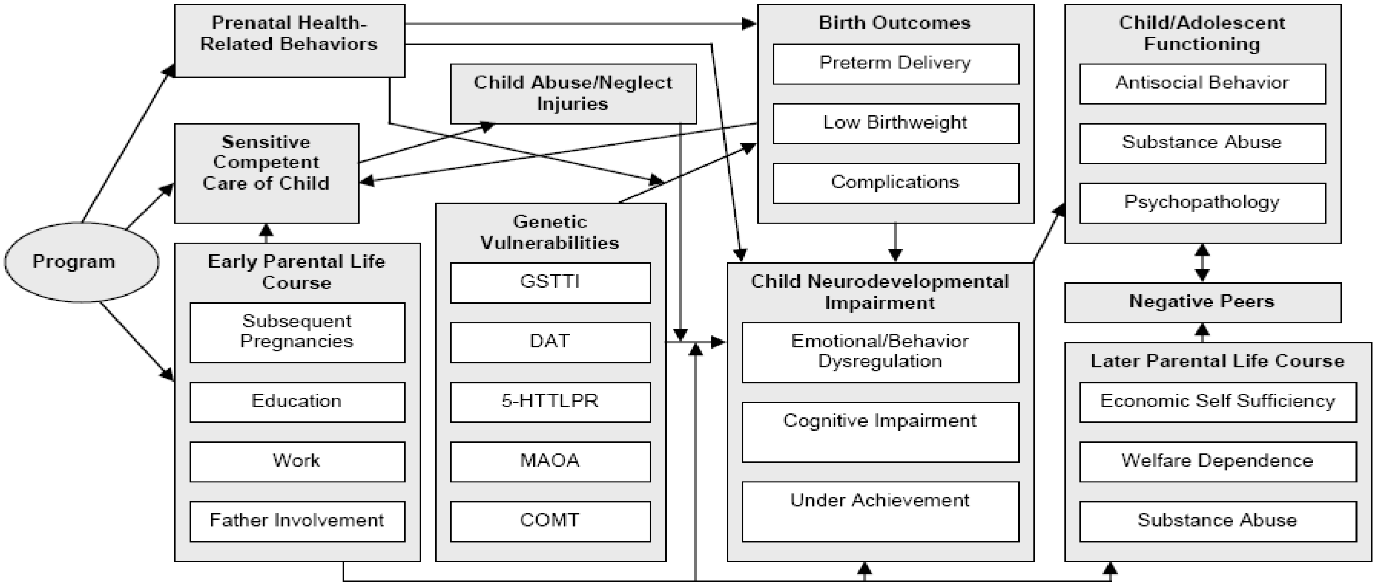


**Figure 1. General Conceptual Model of Program Influences on Maternal and Child Health and Development**

exposure to increase the risk for low birthweight and dysregulated child behavior.[17;18] Reduced exposures to prenatal toxicants, child abuse and neglect, dysregulated parenting, and untoward family environments are expected to shift the child's health and development toward greater behavioral regulation and interpersonal and cognitive competence, including reduced engagement with antisocial peers.

Evidence is accumulating that fetal and postnatal adversity, including prenatal exposures to alcohol and tobacco, produce sustained effects on cellular function and physiology (perinatal programming) which increase the risk for fetal growth restriction and subsequent behavioral and metabolic adaptations that, while increasing the likelihood that individuals will reach reproductive age, have maladaptive consequences for long-term behavioral health and chronic illness, including cardiovascular disease and diabetes[19-22] The effects of these adversities on health and development are hypothesized to be mediated by their direct and indirect effects on the hypothalamic-pituitary-adrenal (HPA) axis: maternal adversity is thought to affect fetal growth through adrenal glucocorticoids; environmental adversity is thought to alter maternal physiology and behavior, which in turn programs HPA activity in the offspring. Thus, the perinatal period and the earliest years of the child's life are likely to be particularly important for the long-term health of humans and an opportune time for preventive intervention.

Nurse-Family Partnership nurses are charged with improving a) pregnancy outcomes by improving women's prenatal health behaviors, b) the child's subsequent health and development by improving parents' early care of the child (especially reducing child maltreatment), and c) families' economic self-sufficiency by helping parents plan subsequent pregnancies and make informed choices about work, education, and partner relations. Their clinical strategies are informed by theories of human-ecology, attachment, and self-efficacy.[9]

***Prenatal Health Behaviors.*** Prenatal tobacco and alcohol exposure increase the risk for fetal growth restriction,[23] preterm birth,[23] and neurodevelopmental impairment (e.g., attention-deficit disorder, cognitive and language delays).[24-31] Children born with subtle neurological perturbations resulting from prenatal exposure to substances are more likely to be irritable and inconsolable,[31-33] making it more difficult for parents to care for them effectively. Improved prenatal health thus also helps parents become competent caregivers. The impacts of tobacco and alcohol exposure on birthweight and dysregulated behavior are moderated by genetic vulnerabilities.[17;18;34] Prenatal tobacco and alcohol use in the Memphis sample, however, were very low, although nurse-visited women, as hypothesized, did have lower rates of pregnancy-induced hypertension. Children exposed to hypertensive disorders of pregnancy and other obstetrical complications are at increased risk for violent aggression during adolescence.[35]

***Sensitive, Competent Care of the Child*.** Parents who empathize with and respond sensitively to their infants' cues are more likely to understand their competencies, leading to less maltreatment and unintentional injuries.[36;37] Competent early parenting is associated with better child behavioral regulation, language, and cognition.[38] Prolonged maltreatment can lead to chronically elevated stress hormones and low child serotonin levels,[39;40] which perturb this system and are implicated in stress-induced delays in neurodevelopment,[41] cognitive dysfunction,[42] dysregulated emotions, and impulsive violence.[43] Early maltreatment is particularly damaging.[44] Later demanding, responsive, and positive parenting can provide some protection from the damaging effects of stressful environments and negative peer influences[45;46] on externalizing symptoms and substance use.[47-51] As outline below, the Memphis program has produced a range of effects which strongly suggest that the nurses prevented child maltreatment and dysregulated caregiving in the first 2 years of life.

***Early Parental Life Course.*** Closely spaced subsequent births undermine unmarried women's education and employment,[52] and limit their time and resources to nurture and protect their children. Married couples are more likely to achieve economic self-sufficiency, and their children are at lower risk for a host of problems.[53] Nurses promote fathers' involvement and help women make appropriate choices about the kinds of men they allow into their lives. The impact of father and partner involvement on child health depends upon the degree to which partners are antisocial.[54;55] Poverty early in life predicts compromised child educational achievement.[56] As noted below, the program has produced earlier effects on maternal life-course, which we expect will endure in the next phase of follow-up, will affect women's susceptibility to depression and SUDs, and will contribute to enduring program effects on the child.[57;58]

***Modifiable Risks for Early-Onset Antisocial Behavior, Substance-Use Disorders, and Depression***. Many of the prenatal and infancy risks addressed by this program are risks for early-onset antisocial behavior, substance use, and depression.[5;6;9;59-61] Prenatal tobacco and alcohol exposures, for example, are putative influences on children's disinhibited behaviors.[62] Children with early-onset conduct problems are more likely to have subtle neurodevelopmental deficits (such as problems with attention, impulsivity, and language)[6;27;31;35] that may contribute to, be caused by, or exacerbated by abusive and rejecting care early in life.[63;64] Moreover, childhood sexual abuse and physical trauma increase the likelihood of

early substance use and risky sexual behaviors which increase the risk for HIV infection.[65;66] Frequent activation of the HPA system resulting from the chronic stress of abuse or neglect can produce high and prolonged glucocorticoid levels, which in turn can damage the developing hippocampus and other brain structures and impair attention, learning, and memory.[67] In studies with rodents, qualities of early care have been found to produce life-long effects on individuals' stress-reactivity independent of genetic background.[22] Thus, adolescents' impulse control, aggression, success in school, substance abuse, and risky sexual behavior are likely to be affected in part by the degree to which they were exposed to neurotoxic substances during pregnancy, or abused or neglected in their earliest years of life, even though these behaviors likely reflect, to some degree, genetic factors shared with their parents.[68]

In humans, aggressive and disinhibited behaviors that emerge prior to puberty are risks for adolescent SUDs,[69;70] antisocial behavior, and risky sexual behavior. Early onset antisocial behavior leads to more serious and violent offending that is distinguished from normative acting out in adolescence.[71;72] Children who develop Major Depressive Disorder (MDD) in childhood, compared to those who develop MDD later, are more likely to have perinatal insults, motor skill deficits, caretaker instability, and criminality, psychopathology and behavioral and socioemotional problems in their family of origin.[55] Youth who use substances perform poorly on neuropsychological tests measuring ECF, especially inattention, impulsivity, risky decision making, and verbal recognition memory.[73] There are indications from the literature on schizophrenia that errors in neuronal wiring during the earliest phases of brain development, while often manifest in early social, cognitive, and motor deficits, may not become fully evident in disabling disorder until synaptic pruning is complete – during late adolescence to young adulthood.[74] Early neuro-protective interventions may thus contribute to a range of functional benefits that are not fully evident until later stages of development.

Both conduct disorder (CD) and early substance use increase the risk for later SUDs and chronic antisocial behavior,[61;63;64;70;75-77]perhaps to some degree because these behaviors lead children into deviant peer groups and social contexts that reinforce their dysregulated behaviors. Moreover, children who begin using cannabis in adolescence (<17 years) are at greater risk for developing SUDs.[77] Adolescent substance use also is implicated in the development of adult antisocial behavior[78] and depression. Mood and anxiety disorders lead to adolescent substance-use problems to a greater extent in females than males.[79]

Integrity of ECF and its modulation of emotional responses to social stimuli may reflect key regulatory processes involved in drug abuse and related psychopathology. Impaired ECF compromises interpretation of social cues and undermines socially adaptive responses to stress.[80] The prefrontal cortex (PFC) is the primary neural regulatory mechanism sub-serving ECF;[81-83] its connections to the limbic system (e.g., the amygdala) modulate emotional responses to environmental and social stimuli.[7;81;82;84-87] Neural mechanisms underlying emotion contribute to impulse control and decision-making that may be modulated by the PFC and limbic system. These functions develop with distinct cognitive and emotional skills maturing at different times[88] that coincide with growth in the frontal lobe.[89-93] Because this neural circuitry is exquisitely sensitive to environmental influences, psychosocial stress due to factors such as abuse and neglect can impair the development of ECF and emotional regulation.[94-99] Prolonged stress exposures, as noted above, can cause chronically elevated stress hormones and perturbations in neurotransmitter activities that may delay the development of this circuitry and compromise cognitive, emotional, and behavioral regulation.[100;101] Given vulnerability of the PFC to stress, neurocognitive deficits may be more prevalent in low-income, high-crime neighborhoods, and families with significant dysfunction. Thus, developmentally relevant dimensions of ECF and emotional perception will be examined at the proposed follow-up, both as outcomes and mediators of outcomes, such as violence and SUDs.[102] Examining these outcomes is important given earlier program effects (discussed below) on hypertensive disorders of pregnancy, outcomes indicative of abuse and neglect (e.g., injury and death for preventable causes) and on children's cognition, achievement, and behavior.

***Substance Use in Memphis Adolescents.*** In the Memphis City Schools in 2003, 30-day rates of cannabis use were 25.5% and 30-day rates of alcohol use were 34.4% among 11$^{th}$ graders.[103] Among 17-year-old substance users, 17% are estimated to have an SUD.[104] This means that there is substantial room for improvement and detection of possible program effects on substance use. The rate of SUDs should be about 6% in the current trial at child age 17; given reductions in early starting substance use at child age 12, there is a strong possibility that program effects will also emerge in reduced SUDs at the next phase of this trial.

***Genetic Vulnerability to Compromised Mothering under Stress.*** Maternal behavior is a highly conserved set of capacities that is crucial for reproductive success.[105] Gene knockout studies and intervention trials with rodents show that perinatal experience is crucial in programming aspects of later maternal behavior;[105] this programming also appears to influence aspects of learning and memory. Many of the brain regions implicated in experimental interventions with rodents are the same as those implicated in mediating

aspects of maternal behavior.[105] Findings with rodents suggest that maternal experience and behavior in the days following birth serves to "program" subsequent maternal behavior in addition to establishing the offspring's level of HPA responsiveness to stress.[106-108] Extreme forms of maternal deprivation have been shown to have profoundly negative effects on the development of maternal behavior in adult non-human primates.[109] Rhesus monkey mothers who had been separated from their mothers at birth had lower levels of adequate caregiving among first-borns than they did among subsequent offspring.[110] The focus of the current intervention, which begins during the pregnancies of mothers with no previous live births, thus coincides with a period in maternal development in which caregiving is most vulnerable to intergenerational risks, current stress, and lack of support, and that may set the stage for future maternal behavior with subsequent offspring. As discussed below, polymorphisms in 5-HTTLPR and COMT val158met, given their putative role in the individual's regulation of stress[(e.g., 111)112;113] and responsiveness to support (5-HTTLPR),[22] may play a role in moderating mothers' abilities to care competently for themselves and their children under conditions of extreme poverty, and teen- single-parenthood like those found in this sample, and may play a role in accounting for the intergenerational transmission of compromised parenting.[81;114-117]

***Child Maltreatment, Adversity, and Genetic Vulnerability to Internalizing Problems, Behavioral Disinhibition, and Substance Use Disorders.*** Child abuse and neglect and early life adversity are non-specific factors that increase the risk for a host of later internalizing and externalizing problems (depression, post traumatic stress disorder (PTSD), CD, antisocial personality disorder, as well as alcohol and substance use disorders)[118-121] but there are substantial differences in the degree to which individuals develop disorders in the context of these early adversities. Polymorphic variations in the 5-HTT and MAOA genes affect the individual's response to adversity, and thus may account for individual differences in the development of psychopathology in the context of prolonged and heightened adversity. Individuals with the short allele of the 5-HTTLPR polymorphism in the *SLC6A4* gene who experience maltreatment and life stress are at heightened risk for major depression, impulsivity, and substance use disorders in humans[111;122-124] and impulsivity and alcohol consumption in primates.[125;126] The literature does reveal inconsistencies, however, some of which may be explained by the age at which individuals experienced stress.[127;128] Social support may moderate the interaction of the low-expression variant of 5HTTLPR with the experience of child maltreatment in predicting depression.[129] While not entirely consistent,[130;131], the balance of evidence indicates that males with low-activity MAOA-LPR alleles and who experience child maltreatment are at greater risk for a variety of mental health problems, including attention deficit/hyperactivity and severe antisocial behavior and than are males with the high-activity MAOA-LPR alleles even if they experience child maltreatment. These gene x environment interactions are likely to increase individuals' risk for SUDs, unprotected sex during alcohol consumption, and HIV infection.[54;132;133]

Given other genetic and environmental moderating influences on development, the story is likely to be more complicated than revealed simply by polymorphisms in these two candidate genes and their interplay with environments.[134] COMT, for example, plays an important role in stress response, but the effect of COMT on the release of adrenocorticotrophin hormone (ACTH) may depend upon the presence of the low-expression variant of MAOA in the same individual.[135;136] Similarly, a recent study of the MAOA-LPR polymorphism and stress in females found that the interaction of stress with MAOA-LPR was supported by analyses that examined corresponding haplotypes as well as specific alleles, reinforcing the importance of the MAOA-system and these vulnerability alleles.[137] The evidence for the role of MAOA and 5-HTT in moderating stress reactivity is thus sufficiently compelling to warrant a disciplined examination of their possible role in accounting for individual differences in the impact of the NFP intervention.

***The Role of 5-HTT in Moderating Stress Reactivity and Risk for Depression.*** The serotonin transporter gene (5-HTT) is involved in the reuptake of serotonin in brain synapses. A functional promoter polymorphism, 5-HTTLPR, consists of a varying number of copies of a 20-23 repeat sequence. Individuals with the short allele "S" (14 repeats) have less efficient transcription, which leads to deficient serotonin reuptake compared to those with the long allele (L) (16 repeats).[111] The lower activity 5-HTTLPR S allele has a significant but quantitatively small role in anxiety/dysphoria[138] and alcoholism;[139] 5-HTT has been identified as a stress resiliency gene, a role confirmed and expanded by neuroimaging studies. The S allele has been associated with greater activation of the amygdala in response to fearful stimuli[140;141] as well as uncoupling of the feedback circuit between the amygdala and the perigenual cingulate that is responsible for the extinction of negative affect. This circuit accounts for 30% of the variance in anxious temperament.[142] Conversely, S allele carriers show greater coupling between the amygdala and ventromedial prefrontal cortex,[141] a fronto-limbic circuit that influences stress responses by the HPA Axis[143] and implicated in mood dysregulation and major depression. It has now been shown that 5-HTTLPR is functionally triallelic.[144] As noted below, at 12 years of

age, nurse-visited children reported fewer internalizing disorders than did those in the control group. We will examine the extent to which these earlier program effects and those hypothesized depression and anxiety at age 17 are concentrated in those who are particularly vulnerable to stress as a results of their carrying the 5-HTTLPR low activity alleles,

***The Role of MAOA in Moderating Stress Reactivity and Risk for Impulsivity, Violence, and SUDs.*** MAOA is on the X-chromosome and encodes the enzyme Monoamine Oxidase A (MAOA; EC 1.4.3.4), which metabolizes monoamines, including norepinephrine (NE), dopamine (DA), and serotonin (5-HT). A common functional variable number tandem repeat (VNTR) polymorphism in the promoter region of the monoamine oxidase A (MAOA) gene has been found to have five alleles containing 2, 3, 3.5, 4, and 5 copies of a 30-base pair (bp) tandem repeat that influences transcription. Enzyme expression is 2-10 times higher for the 3.5 and 4 repeats than for the 3 repeat.[145] The low activity variant has been of particular interest in explaining the high rates of aggression, delinquency, and substance use disorders found in males,[145;146] especially in the presence of early adversity.[132] Given that the MAOA gene is located on the X chromosome, there is greater statistical power to examine the relationship between early life adversity and the low-activity MAOA-LPR variant among males. There is less consistency in this relationship among females, perhaps because of lower statistical power due to the lower prevalence of the low-activity genotype among females, or perhaps because sex hormones such as testosterone interact with this MAOA genotype.[147] On the other hand, the MAOA gene appears to escape X-inactivation in females.[148] This conceivably results in higher brain MAOA expression for female high activity homozygotes than male high activity hemizygotes. Indeed, a recent study among American Indian females found that MAOA-LPR variant in combination with childhood sexual abuse increased the risk for alcohol use disorders (AUD) and especially AUD in combination with antisocial personality disorders.[137] In one study, the low-activity MAOA-LPR variant was associated with children's experience of abuse and neglect,[149] which emphasizes the importance of examining possible GE correlations in these types of studies. In the current study, as shown below, there are indications that males in the intervention group are showing the greatest benefits in conduct at school and academic achievement. We will examine the extent to which these benefits for males are particularly pronounced for those with the low activity MAOA-LPR alleles.

***COMT Polymorphism and Response to Stress.*** Catechol-O-methyltransferase (COMT) plays an important role in the metabolism of CNS dopamine and norepinephrine in the prefrontal cortex. To date, there is no evidence to suggest a gene x environment interaction for COMT, but there is reason to believe that such interactions might exist. A common COMT polymorphism is responsible for a 3 to 4 fold variation in enzyme activity.[150] The frequency of the lower activity Met158 allele (COMT-L) is 0.22–0.38 in African-Americans and 0.44–0.53 in Caucasians. The higher activity Val158 allele (COMT-H) is related to compromised ECF,[112;151-153] drug abuse[154-156] and adult psychosis in adolescent cannabis users.[113] Moreover, recent evidence indicates that children with ADHD are more likely to develop childhood-onset CD if they have both the val/val genotype and have experienced prenatal adversity (reflected by low birthweight).[157] The Met158 allele, on the other hand, is linked with anxious temperaments, increased emotionality, and decreased pain threshold.[158-160] Thus, COMT genotype appears to play an important role in the balance between emotional resilience and vulnerability to stress. The putative increased vulnerability of Met158 allele carriers to stress may be more apparent in women because of sexually dimorphic effects in the COMT-anxiety association.[158;159] The limited number of women likely to carry the Met158 allele in this sample, however, prevents us from examining this subgroup as a moderator of treatment impact. As noted below, we have seen program effects on outcomes such as early starting substance use and compromised academic performance among children. We also see a trend for nurse-visited women to report lower use of substances at child age 9. We wish to see whether these effects are more pronounced among those who carry 2 copies of the Val158 allele, and whether those with this genotype benefit the most from the intervention with respect to maternal and child SUDs at child age 17.

***Adolescent Substance Use, Risky Sexual Behavior, and Risk for HIV.*** Neurobehavioral disinhibition reflects a latent trait characterized by behavioral under-control, affect dysregulation, and compromised ECF that increases youths' early-starting substance use and risk for SUD.[69;161] Elements of this trait, such as sensation seeking, increase individuals' tendencies to engage frequently in risky sexual behavior.[162] It is thus relevant that 49% of pregnant mothers enrolled in the current trial were <18 at registration. It is likely that compromised ECF mediates the relationship between the Val/Val genotype and risky sexual behavior, given that Val/Val undermines ECF and increases risk for polysubstance abuse.[156] Adolescent alcohol use increases risk for sexually transmitted diseases (STDs).[163;164]

While infrequently occurring (48 per 100,000), HIV infection is increased among low-income, African-American women living in the South, especially among substance users and those with STDs.[165;166] There is increased efficiency of transmission of HIV in the presence of co-infection with bacterial STDs or

trichomoniasis.[167-169] Among sexually active 14–18-year-old females in Birmingham AL, 28.7% had at least one STD (either *N gonorrhoeae, C. trachomatis, or T vaginalis)* and 5.5% had two or more and infrequent parental monitoring predicted a 2-fold increase in infection.[170] In 2003, 55% of African-American 11th graders in Memphis City Schools had had sexual intercourse within 3 months of the interview; 73% had ever had intercourse, while 31% reported four or more sexual partners by the 11th grade.[103]

Given the health problems to be examined in the next phase of follow-up in this trial, it is important to emphasize that the NFP program reduced many of the prenatal, infant, and childhood factors discussed above that increase risk for violent antisocial behavior, psychopathology, SUDs and HIV infection.

## C. PRELIMINARY STUDIES

Tested with a primarily white sample in the Elmira trial, the NFP program produced effects consistent with the program model, that on average tended to be greater for families at greater social disadvantage and where mothers were more psychologically vulnerable.[9] These effects were translated into cost savings.[171;172] The Memphis trial was designed to test the effects of the program with a large sample of very low-income African-Americans living in a major urban area, when the program was administered through a local health department, and the program developers had limited involvement in its implementation. In this trial, 1,139 low-income pregnant women (98% unmarried, 67% <19 years old, 92% African-American) were randomly assigned to experimental or comparison services; 743 were followed after delivery. The sample has resided in extraordinarily stressful neighborhoods and has endured extreme poverty. At registration, the mean level of neighborhood disorganization (assessed by census tract data at the block group and using the Lauritsen scale[173]) was 3.43 SD above the national mean, i.e., the average level of adversity in the sample neighborhoods was among the worst in the nation (in the top 1000th). 85% of the sample had incomes below the federal poverty guidelines. Design details are provided below and in the published articles.

### Memphis Results

Given results in Elmira, we hypothesized that program effects on parenting and child outcomes would be greater for the group defined by mothers having fewer psychological resources (poorer mental health, intellectual functioning, and self-efficacy/mastery). We found support for this hypothesis and that program effects on maternal life-course, especially in planning subsequent pregnancies, were concentrated among women with initially higher levels of psychological resources. We interpreted this as a reflection of nurse-visited higher-resource women's ability to secure employment and manage care of their children simultaneously, providing high-resource mothers with motivation to plan future pregnancies; nurse-visited low-resource mothers had difficulty balancing these tasks and envisioning success in the world of work, so they had fewer reasons to plan future pregnancies, and instead focused their resources on care of their children.

### Maternal and Infant Outcomes

***Prenatal Health, Care-Giving, and Injuries.*** Nurse-visited mothers, compared to control-group counterparts, exhibited superior prenatal health behaviors, fewer obstetric complications and infections, and better care of their children; their children had fewer health-care encounters for injuries/ingestions (Table 1). Program effects on care-giving and injuries were greater for mothers with low psychological resources. Figure 2 shows regressions of number of days children were hospitalized for injuries or ingestions from birth to age 2 on maternal psychological resources, fitted separately by treatment assignment. This figure emphasizes that the program effects on injuries and other child outcomes were concentrated on children born to mothers at greatest risk for compromised care-giving because of their limited psychological resources. Program impact on days hospitalized for injuries/ ingestions was limited to children with mothers in the lower half of the maternal psychological

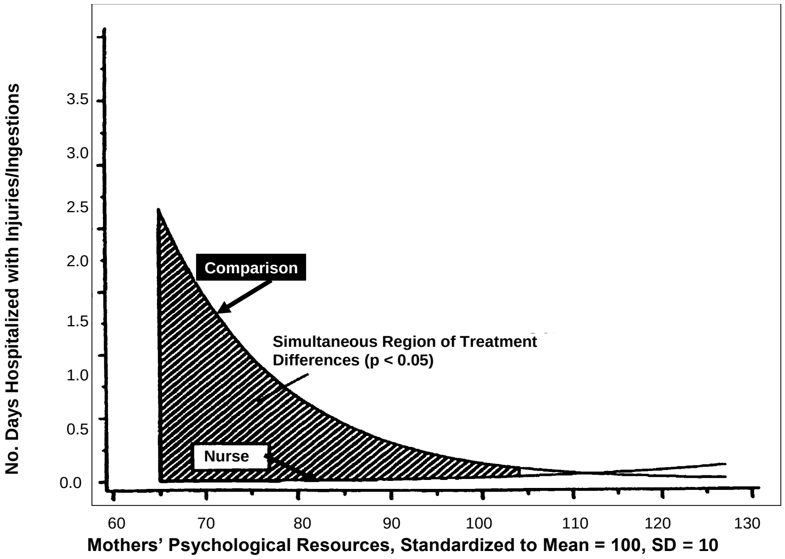


**Figure 2. Number days children hospitalized for injuries/ingestions (0-2 yr) by maternal psychological resources and treatment condition - Memphis**

**Table 1. Program effects on selected prenatal health, care-giving, injuries, maternal life-course, and child outcomes**

| Variable | Sample | Comparison | Nurse | Comp vs. Nurse | |
|---|---|---|---|---|---|
| **Prenatal Health after Enrollment** | | **Mean/Rate** | **Mean/Rate** | **p-value** | **Effect size*** |
| Pregnancy induced hypertension, % | Whole | 20.0 | 13.0 | .009 | OR=0.65 |
| Incidence of yeast infections after randomization | Whole | 0.19 | 0.14 | .05 | IR=0.74 |
| **Sensitive, Competent Care of Child** | | | | | |
| Beliefs associated with child abuse,<br>Bavolek total score, 6-24 mo | Whole<br>Low-Resource | 100.5<br>102.5 | 98.7<br>100.2 | .003<br>≤.01 | ES=-0.23<br>ES=-0.29 |
| Emotional/cognitive stimulation (Home total score) 12 & 24 mo | Whole<br>Low-Resource | 30.9<br>30.3 | 32.3<br>31.5 | .003<br>≤.05 | ES=0.24<br>ES=0.21 |
| Child responsiveness, NCAST child total score, 6-24 mo | Whole<br>Low-Resource | 17.4<br>17.2 | 17.7<br>17.9 | NS<br>≤.05 | ES=-0.09<br>ES=-0.19 |
| **Injuries/Ingestions in Medical Record** | | | | | |
| Incidence of encounters (all types)—<br>Injuries/ingestions, 0-24 mo | Whole<br>Low-Resource | 0.56<br>0.67 | 0.43<br>0.41 | .05<br>≤.01 | IR=0.77<br>IR=0.61 |
| Incidence of hospitalizations—Injuries/ingestions, 0-24 mo | Whole<br>Low-Resource | 0.03<br>0.04 | 0.01<br>0.01 | .01<br><.10 | IR=0.33<br>IR=0.25 |
| Incidence of days hospitalized—Injuries/ingestions, 0-24 mo | Whole<br>Low-Resource | 0.18<br>0.26 | 0.04<br>0.02 | .0003<br>≤.01 | IR=0.22<br>IR=0.08 |
| **Maternal Life-Course** | | | | | |
| Count of Substances Used – child age 9 | Whole | 0.17 | 0.10 | .075 | IR=0.62 |
| Count of closely spaced subsequent births – age 12 | Whole | 0.51 | 0.34 | .019 | IR=0.67 |
| No. of Months with Partner, 6-12 years | Whole | 57.89 | 68.11 | .010 | ES=-0.20 |
| Food stamps use (avg. mos./yr), 0-12 years | Whole | 7.19 | 6.54 | .022 | ES=0.13 |
| AFDC/TANF use (avg. mos./yr), 0-12 years | Whole | 5.30 | 4.69 | .030 | ES=0.12 |
| **Cognition, Language, & Achievement** | | | | | |
| Mental Processing Composite (KABC) - age 6 | Whole<br>Low-Resource | 90.24<br>87.64 | 92.34<br>90.49 | .03<br>.03 | ES=0.18<br>ES=0.25 |
| Receptive vocabulary (PPVT) - age 6 | Whole<br>Low-Resource | 82.13<br>79.08 | 84.32<br>81.75 | .04<br>.07 | ES=0.17<br>ES=0.21 |
| Academic Achievement (reading + math) – age 6 | Whole<br>Low-Resource | 91.17<br>88.69 | 91.65<br>91.07 | .630<br>.084 | ES=0.04<br>ES=0.19 |
| Academic Achievement (reading + math) – age 12 | Whole<br>Low-Resource | 87.94<br>85.66 | 89.23<br>88.77 | .140<br>.009 | ES=0.12<br>ES=0.29 |
| **Emotional/Behavioral Regulation, Mental Health, Substance Use, Death – First Born** | | | | | |
| Dysregulated Aggression- MSSB - age 6 | Whole<br>Low-Resource | 100.26<br>101.10 | 99.24<br>98.58 | .26<br>.04 | ES=-0.10<br>ES=-0.25 |
| Incoherent Stories- MSSB - age 6 | Whole<br>Low-Resource | 25.22<br>29.84 | 21.15<br>20.90 | .07<br>.006 | ES=-0.16<br>ES=-0.34 |
| % Total Problems - Borderline/Clinical - CLCB - age 6 | Whole | 5.4 | 1.8 | .04 | OR=0.32 |
| Count of Failed Conduct – males (grades 1-6) | Whole | 0.10 | 0.06 | .044 | IR=0.56 |
| Infant/Childhood Death 0-9 years | Whole | 20.08/1000 | 4.5/1000 | .080 | OR=0.22 |
| % used alcohol, cigarettes, or cannabis - age 12 | Whole | 5.2 | 1.6 | .024 | OR=0.29 |
| No. days used substances-last 30 days - age 12 | Whole | 0.18 | 0.03 | <.001 | IR=0.17 |
| Ever Sent to Juvenile Detention - age 12 | Whole | 9.4 | 7.2 | .080 | OR=0.52 |
| % Internalizing Disorders (borderline/clinical – age12 | Whole | 31 | 22 | .044 | OR=0.63 |

*Note: OR = Odds Ratio; IR= Incidence Ratio; ES= Effect Size (mean difference/standard deviation)

resource distribution (standardized to a mean of 100 and a standard deviation of 10). Table 1 shows that program effects on most child outcomes were greater for those born to the most vulnerable mothers. Discerning program impact on child abuse and neglect must be inferred from several sources of data (e.g., treatment-control differences in children's injuries revealed in their medical records, observations of infants' responses to their parents in laboratory observation paradigms), as Tennessee child protection and child welfare records are not available prior to 2001, given inadequacies in their record keeping system. Moreover, such records under-estimate abuse[174] and are subject to surveillance bias.[175]

**Figure 3. Intervention effect sizes in academic achievement at 6 and 12 years of age-- children born to low-resource mothers**

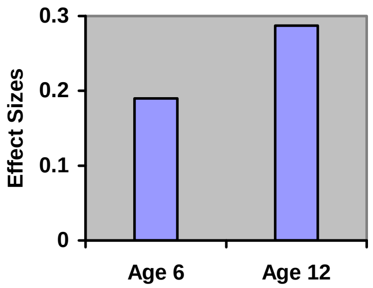


***Maternal Life-Course.*** The program also produced consistent effects on maternal life-course (e.g., increased stability of partner relationships; reduced fertility, use of food stamps, and welfare). Effects on fertility outcomes like closely spaced subsequent births (<2 years) were more pronounced

for higher-resource mothers. At child age 9, nurse-visited mothers reported (p=.075) using fewer different types of substances.

**Child Functioning in Elementary/Middle School**

As shown in Table 1, the program improved child functioning from age 6 to 12. Effects were greater for children born to low-resource mothers, given the particularly poor functioning of those in the control-group.

***Cognition, Language, and Achievement.*** The program improved children's cognition and language at age 6. Between age 6 and 12, the program continued to improve children's academic achievement test scores, an effect limited to children born to mothers with low psychological resources. Importantly, program impact on achievement among children born to low-resource mothers increased in effect sizes from 6 to 12 years (see Table 1 and Figure 3) as cognitive demands of the tests increased. These effects were particularly strong for males and in math achievement and give us reason to expect continued growth in impact as children mature.

***Mental Health, Behavioral Regulation, Substance Use, and Death.*** Beginning at child age 6, program effects began to emerge on first born children's emotional and behavioral regulation. Nurse-visited children born to low-resource mothers revealed less dysregulated aggression and incoherence in response to the MacArthur Story Stems.[176] The program effect on coherence was particularly strong in the presence of high emotional arousal,[177] a pattern consistent with greater PFC moderation of limbic system reactions to stress.[158] Over children's first 6 years of elementary school, as predicted, nurse-visited males (but not females) had lower rates of failed conduct than did males in the control group (Figure 4), effects that may be more pronounced for males with the low activity alleles of the MAOA-LRP polymorphism.

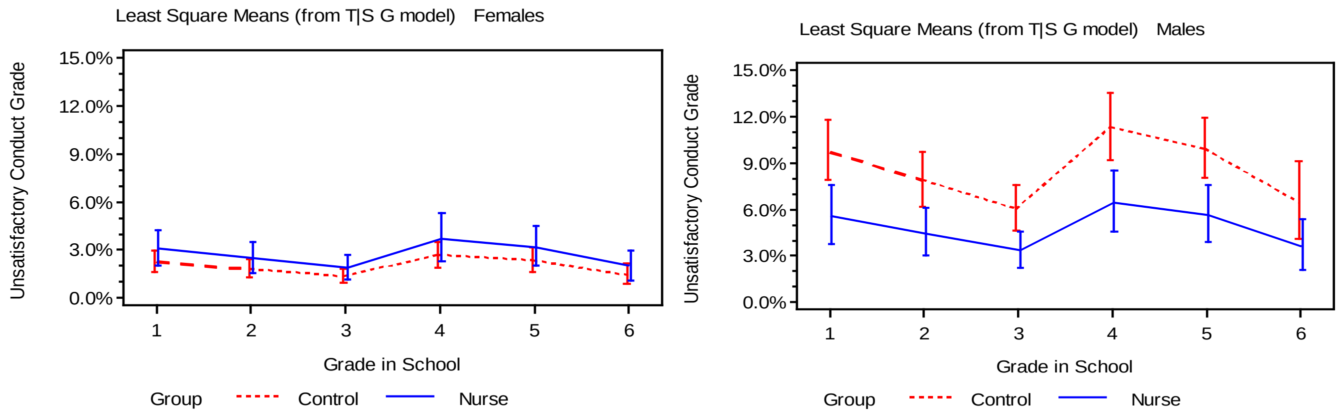


**OR$_{females}$= 1.47; p=.399 OR$_{males}$= 0.55; p=.054**

**Figure 4. Rates of failed (unsatisfactory) conduct in the first 6 years of elementary school by child gender and treatment**

By age 12, nurse-visited children reported fewer internalizing disorders (borderline/clinical range) on the CBCL (Figure 5). By age 12, nurse-visited children also reported substantially lower rates of 30-day tobacco, alcohol and marijuana use; fewer days of using the 3 substances, and using fewer different types of substances. Figure 6 shows program impacts on whether children had used substances in the 30-day interval prior to the interview. The program effect on early starting substance use was even greater when the counts of days of using substances or the counts of types of substances (indicators of greater severity) were used as the outcomes. We expect to find corresponding effects for SUDs for the 17-year-old youth at the next phase of follow-up.

**Figure 5. Percent Child Internalizing Disorders – Memphis – Child Age 12**

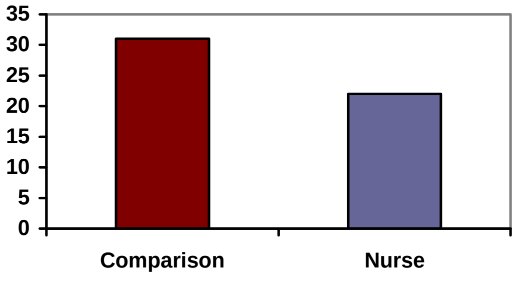


P = .044, OR = 0.63

Of particular note, there was a trend (p=.08) for nurse-visited firstborn children to die less frequently in the first 9 years of the child's life (10/498 vs. 1/222 live births); 9 of the 10 control-group deaths were due to preventable causes (preterm delivery, Sudden Infant Death Syndrome, or injury); one death in the control group and the only death in the nurse-visited group were due to non-preventable causes (chromosomal anomalies, multiple congenital anomalies).[178] Two of the injury deaths in the control group were by firearm. By age 12, another nurse-visited child had died due to a brain tumor (not preventable). When viewed in light of earlier program effects on childhood injuries, these mortality

data suggest that nurses helped parents reduce their children's exposure to serious, life-threatening adversities.

Earlier program impacts on maternal mastery, partner stability, inter-birth intervals, and welfare use all helped mediate program effects on the socio-emotional health of the 6-year-olds, a pattern of results consistent with the theory shown in Figure 1. These findings need to be understood in the context of data on the life-course development of antisocial behavior. It is generally accepted that children who begin expressing antisocial behavior early in life (typically before puberty) are at heightened risk for violent, life-course persistent offending, and antisocial personality disorder.[63] Moreover, early dysregulated behavior is best predicted by interactions between neurodevelopmental problems and child maltreatment.[63] The more prevalent, but less serious adolescent-limited form of antisocial behavior[63;179] is thus less amenable to this intervention. This program is thus most likely to reduce early-onset, violent, persistent antisocial behavior.

**Figure 6. Percent Child Used Substances – Memphis – Child Age 12**

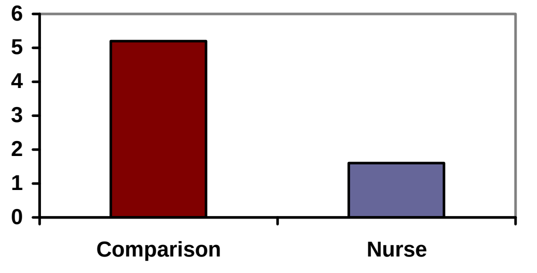


P = .024, OR = 0.29

The program effect on early substance use is particularly important, as early starters are at much greater risk for SUDs.[59] We conducted analyses in the control group that compared those who used any substance (n=20) by age 12 to the rest of the control group (also highly disadvantaged), and found that those who did had much poorer early functioning than those who abstained; space limitations prevent elaboration. The program reduced many of the earlier stressors and adverse outcomes associated with starting early. We also compared those in the control group who by age 12 had self-reported internalizing disorders (n=126) to those who did not and found that those with internalizing disorders also were at significantly greater risk than their counterparts without internalizing disorders (n=268). As with early starting substance use, the program affected many of the earlier stressors and child outcomes associated with age-12 internalizing disorders.

***Implications for Later Psychopathology and SUDs.*** Program effects on children's academic, mental health, and behavioral functioning, including emergent use of substances through child age 12, lead us to hypothesize that the program will continue to affect serious antisocial behavior in mid-adolescence, when risk-taking and antisocial behaviors reach their peak. Given that program impact on academic achievement is particularly strong for boys born to low-resource mothers and that boys born to low-resource mothers in the control group are declining in academic achievement over time, we expect that 12-year-old control-group children who used substances are at much greater risk for developing SUDs and becoming more violent and antisocial as they reach mid-adolescence. We expect that children's substance use and abuse will increase by age 17, but especially among control-group males, since they will have fewer attachments to school and conventional life prospects due in part to increasing problems in school. These findings lead us to expect greater differentiation of program effects by child gender at age 17. For boys, we expect that the program will reduce violent offending, involvement in the criminal justice system, and SUDs as these problems become more prevalent. Among females, we expect to see increasing program effects on depression/anxiety and SUDs.

***Hypothesized Greater Effect on Mothers Who Experience Stress and Are Genetically Susceptible***

The degree of stress and support experienced by mothers in the period surrounding birth may have long-lasting effects on stress-reactivity in the offspring as well as long-lasting effects on maternal care-giving.[106-108;114] We therefore have hypothesized that genetically-based individual differences in maternal stress reactivity may affect the degree to which care is compromised in the presence of environmental adversity and the degree to which the program is beneficial to particular mothers (Figure 7). The greater effect of the program on qualities of care-giving and children born to mothers with low psychological resources may reflect, at lest in part, moderation of genetic vulnerabilities to stress (low activity alleles of 5-HTTLPR) and compromised ECF (COMT high activity alleles) under the significant levels of family- and neighborhood poverty found in the participants in the Memphis trial. Mothers with 2 copies of the low-activity variants of the 5-HTTLPR polymorphism giving birth for the first time, for example, may be particularly sensitive to developing internalizing disorders and compromised caregiving in response to environmental stressors, which may make this sub-group more responsive to the beneficial effects of the intervention. Mothers with two copies of the high-activity alleles of the COMT polymorphism, on the other hand, may be more susceptible to compromised

ECF in the presence of stress, which in turn may make them more susceptible to impulsive behavior and substance abuse under conditions of stress, which creates a separate pathway through which maternal care may be compromised in environments characterized by stress, disorganization, and pressures to engage in substance use and other antisocial behaviors.

### *Hypothesized Program Impact on Genetically Susceptible Children*

Program effects on children observed to date tend to be greater among those born to mothers who at registration lacked the resources to manage adversity in their lives (low psychological resources). Figure 7 shows more precisely how the program is hypothesized to moderate the interaction of stressful environmental conditions with the low activity variants of 5-HTTLPR and MAOA-LPR in the children. The nurses are thought to reduce stress on the developing child in two primary ways. The first involves the nurses' directly reducing child abuse and neglect. The second involves the nurses' activation of parents' instincts to protect their children from threats such as abusive boyfriends or gang activities in the neighborhood. We have only indirect evidence to support these hypothesized mechanisms centered on parental care, but the evidence summarized above (e.g., reduced injuries and deaths for preventable causes, reductions in parents' beliefs associated with child maltreatment) supports this characterization of program impact. By reducing child maltreatment and protecting their children from threatening environments, nurse-visited mothers are hypothesized to mitigate stressful environments that in the presence of the low activity MAOA-LPR variant in male children increase risk for impulsivity and violence[132] (cf. figure 4 above) and that in the presence of the low activity variants of 5-HTTLPR increase risk for depression[111] (cf. figure 5 above).

**Figure 7. Hypothesized program moderation of gene-environment interactions that increase behavioral and emotional dysregulation**

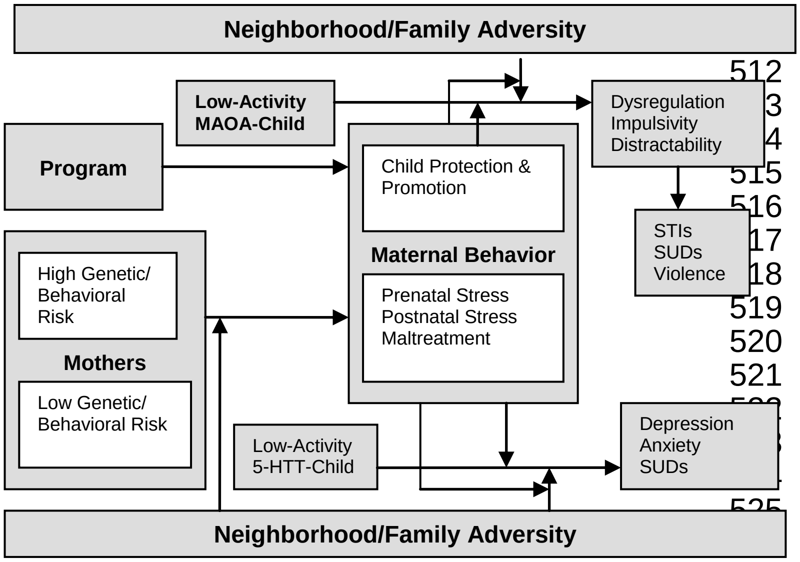


With improved behavioral regulation, language, and cognitive skills throughout their first 12 years of life, nurse-visited children experience greater success in school, which increases their motivation to find socially acceptable roles and avoid life styles that increase their susceptibility to SUDs, risky sexual behavior, pregnancy, HIV exposure, gang membership, and violence, effects that we hypothesize are more frequently occurring among children who are both genetically vulnerable and exposed to highly stressful environments, especially early in development. Moreover, to the extent that mothers have fewer closely spaced subsequent births, they have time and resources to protect those children they do have and to improve their economic conditions. While we expect program effects to be evident at both the main effect level and for those born to low-resource mothers, we think that the hypothesized genetic factors will further elucidate those at risk and those who benefit from the intervention.

### **Focus-Group and Survey Aimed at Evaluating Physical Health Assessments for 17-Year Follow-Up**

We will include a physical health assessment of the adolescents as part of the 17-year follow-up. We will measure weight, height, and blood pressure; assay urine to detect STDs and substance use, and saliva to collect DNA. We conducted focus groups among 20 15-year old male and female participants in our pilot sample and their mothers (before planning to collect DNA). The 20 mothers and youth were provided detailed information on the procedures, and the adolescents went through the health assessment procedures except providing saliva for DNA. For mothers, we assessed their belief in the importance of screening for substances and STDs, their willingness to have their children screened for these health risks, their comfort with not knowing the results of the screens, and their thoughts about improving our plans for assessment. For the 15-year olds, we assessed their comfort with this assessment, belief in the importance of conducting these assessments, belief that results would be kept confidential, and recommendations for improving procedures.

Mothers were eager to have their children tested. They unanimously recognized the importance of STD and drug screening and were supportive of the tests being performed, even if they would not learn the test results. Mothers wanted the tests to be conducted, given the prevalence of these conditions and the inclusion

of treatment and counseling following positive screens. While they would prefer to know the results, they understood the need to keep the information confidential to gain children's consent. Both boys and girls found the testing acceptable but did not trust the investigators to keep the results of the tests confidential. Having written documentation that test results would not be shared with their parents would reduce that distrust. These insights will be used in framing main-study procedures. We will conduct a full-fledged pilot of our procedures in order to develop a final data gathering plan in the 17-year follow-up.

Given that we had not planned to gather DNA at the time of the original proposal, we subsequently conducted a survey of 32 mothers and their 17-year-old children to assess the feasibility and acceptability of our data gathering plan, including gathering saliva for assessing DNA. Mothers and children either had been part of our original pilot sample for this trial or were main study participants. As part of this interview, we asked both mothers and youth whether they would provide DNA for the purposes of this study. There were 2 choices for the mothers and 1 for the youth: would mothers provide their own DNA and would they provide consent for their children to provide the DNA; and would the youth provide consent to provide their own DNA? 91% of the mothers (n=29) agreed to provide their own DNA and to provide consent for their children to provide DNA. 100% of the youth (n=32) agreed to provide DNA. These high rates of agreement likely reflect the long-lasting, trusting relationship established between study participants and the Memphis data-gathering team.

**Why Conduct a Follow-Up of the Memphis Sample at Child Age 17?**

***1) It is of considerable importance to know whether a public health intervention that improves prenatal health, reduces indicators of child abuse and neglect and closely spaced subsequent pregnancies, and improves stable partner relations, economic well-being, and child health and development early in life can reduce youth SUDs, psychopathology, violent crime, gang membership, and risk for HIV infection.*** Most prevention programs are school-based and focus on proximal risks for substance use when children are pre-adolescents. This program targets prenatal and infant risks through child age 2. Given that this program affects a large number of important outcomes at earlier phases of development, it reduces risks for adolescent health problems not addressed in family- or school-based interventions that begin later. This innovative study thus provides an unprecedented opportunity to learn whether a very early intervention can reduce the development of significant health problems during adolescence.

***2) African-American youth living in the south are at high risk for sexually transmitted diseases and SUDs, behaviors that increase their risk for HIV.*** Knowing whether this highly regarded early intervention can reduce risk for HIV infection is of considerable scientific and public health importance.

***3) It is important to learn whether the effects of this program are particularly pronounced among individuals who are genetically susceptible for psychopathology and SUDs under conditions of extreme poverty and unusually high-risk neighborhood adversity.*** We know of no other preventive intervention tested in an RCT that has been examined from the standpoint of its possible attenuation of environmental risks thought to interact with genetic vulnerabilities.

***4) We know very little about the interplay of genetic vulnerability and environmental stress as contributors to behavioral problems among African-Americans, irrespective of intervention effects.***

***5) The age at which we are proposing to conduct this follow-up is near the peak for large upswings in violence, delinquency, and the initiation of SUDs.*** By examining the impact of the intervention at this age, we will be able to discern its impact at a critical transition in development that will provide insight into how behaviors in adolescence contribute to long-term disorder.

***6) The proposed follow-up presents a unique opportunity to assess adolescents on whom detailed data are available on the course of pregnancy, infant health and development, and earlier stages of child development with a sample that is quite homogeneous from the standpoint of its racial background and experience of extreme poverty.*** In spite of surface-level homogeneity, the courses of development and adaptation for individual youth and their families in this impoverished sample are quite heterogeneous. This study thus provides a unique opportunity to examine the extent to which the NFP intervention promotes the health of youth living in extreme poverty and links prenatal and infancy health and development with later life success.

## D. RESEARCH DESIGN AND METHODS

**Sampling Design**

Descriptions of the research and program can be found in our published reports.[177;180;181]

***Original Sample Characteristics.*** We invited 1,290 women from the obstetrical clinic of the Regional Medical Center in Memphis to participate. We recruited women <29 weeks pregnant if they had no previous

live births, no specific chronic illnesses thought to contribute to fetal growth retardation or preterm delivery, and at least 2 sociodemographic risks: a) unmarried, b) less than 12 years of education, c) unemployed. Eighty-eight percent (1139/1290) completed informed consent and were randomized to 1 of 4 treatment conditions described below. All low-income women in Memphis registered for prenatal care at this clinic, which meant that we enrolled nearly the entire population during the 1990–1991 enrollment period. The study was designed to follow 743 families in two treatment conditions after delivery. 92% of the women were African-American, 98% were unmarried, 67% were <19 years of age at registration, 85% came from households with incomes < federal poverty guidelines, and 9% smoked cigarettes. At randomization, there were no treatment differences in women's background characteristics.

***Randomization.*** After the completion of informed consent and baseline interviews, identifying information on participants was entered into a computer program that randomized women to treatment conditions. The randomization methods were extensions of Soares and Wu.[182] The model on which we based the randomization included 5 classification factors: maternal race, chronological age (4 levels), gestational age at enrollment, employment status of head of household, and geographic region of residence (4 regions).

***Statistical Power in the Original Design.*** We conducted power calculations to determine the number and proportion of subjects to assign to treatment and control conditions while minimizing costs in the initial study period (prenatal through child age 2). This allowed disproportionate assignment of women to treatment and control conditions and a reduction in number of families followed into the postnatal phase of the trial. Given high rates of sample retention, the original power calculations hold (see "Overview of Primary Statistical Methods).

**Treatment Conditions**

***Treatment 1 - Transportation During Pregnancy.*** The 166 families in this treatment condition received free round-trip taxicab transportation for scheduled prenatal care appointments. This group did not receive any postpartum services or assessments.

***Treatment 2 - Transportation During Pregnancy and Screening During Infancy.*** The 515 families in this group received: 1) free transportation for scheduled prenatal care; and 2) developmental screening and referral services for the child at the 6th, 12th, and 24th months of the child's life.

***Treatment 3 - Transportation and Nurse-Visitation during Pregnancy Only.*** The 230 families in this treatment condition received: 1) free transportation for scheduled prenatal care; and 2) intensive nurse home-visitation services during pregnancy and one postpartum visit in the hospital before discharge and one postpartum visit in the home. This group did not receive any postpartum services or assessments.

***Treatment 4 - Transportation and Nurse-Visitation during Pregnancy and Infancy.*** The 228 families in this condition received: 1) free transportation for scheduled prenatal care; 2) intensive nurse home-visitation services during pregnancy and through the child's second birthday; and 3) developmental screening and referral services for the child at the 6th, 12th, and 24th months of the child's life.

**Program Plan and Implementation**

The home-visitation program was carried out by the Memphis/Shelby County Health Department during a nursing shortage, which contributed to staff turnover during the conduct of the trial. Program designers were minimally involved in program implementation. These features of program implementation, along with registration of nearly the entire population, make this nearly an effectiveness trial on the efficacy/effectiveness spectrum.

**Possible Attrition Bias during Earlier Phases of the Study**

During the postnatal phase of the trial, we have followed only families assigned to treatments 2 (n=515) and 4 (n=228). We have been remarkably successful in retaining the sample through the most recent follow-up (Table 2). At child age 12, we completed assessments on 593 women and 577 children, for completion rates of 80% and 78% of those originally randomized and 85% and 83% of those where the mother and child were alive and the child not adopted. These assessments do not include an additional 19 cases where the biological mother was not the custodial parent and an interview was conducted with the child's guardian.

**Research Activities for the Current Phase of Follow Up**

The proposed study consists of a longitudinal follow-up of 670 women and firstborn children who had not dropped out of the study by the child's 12th year. We have assumed for purposes of calculating statistical power that we will conduct assessments on 600 mothers and firstborn children; this is slightly less than the

number of maternal and other-custody interviews conducted at child age 12. We actually expect to be more successful in completing assessments at the next phase of follow-up than we were at child age 12 because of our offering greater honoraria for data gathering than we have in the past.

**Table 2. Sample Recruitment, randomization, attrition, and completed assessments at previous phases of the trial**

| **Eligible Subjects Invited to Participate** | 1290 | | | | |
|---|---|---|---|---|---|
| Number Refused | 151 | | | | |
| Number Randomized | 1139 | | | | |
| **Treatment Group Assignment** | **1** | **2** | **3** | **4** | **Total** |
| Number Allocated to Each Treatment | 166 | 515 | 230 | 228 | 1139 |
| Miscarriages | 6 | 19 | 6 | 8 | 39 |
| Stillbirths | 0 | 5 | 3 | 2 | 10 |
| Infant and childhood deaths (0-12 years) | NA | 10 | NA | 2 | 12 |
| Maternal Deaths (though child age 12) | | 8 | | 2 | 10 |
| Post-randomization Refusals/drops (through age 12) | 1 | 11 | 4 | 6 | 22 |
| No. (%) of randomized available for 17-yr follow-up | | 462 (90%) | | 208 (91%) | 670 (90%) |
| **Completed 12-yr Assessments (no. (%) of randomized)** | | | | | |
| Maternal interviews | | 403 (78%) | | 186 (82%) | 593 (80%) |
| Other custody interviews (no maternal interview) | | 10 (2%) | | 9 (4%) | 19 (3%) |
| Child interview | | 395 (77%) | | 180 (79%) | 577 (78%) |
| Teacher reports (firstborn child) | | 368 (71%) | | 167 (73%) | 535 (72%) |

## Tracing the Sample

This is an unusually stable sample. At the 12-year assessment, of those available for follow-up (no previous deaths or refusals), 95% lived within a 2-hour drive of Memphis. The remaining 5% lived at distances that made driving to the study offices impractical. For individuals who moved since their last assessment, we will rely upon their Medicaid numbers, TANF records, social security numbers, and other archival data in addition to "contact people" to trace them. We have budgeted airfare and per diem for staff to fly to assess the 5% who are not within driving distance.

## Assessment of Outcomes

In choosing outcome measures for this phase of the trial, we have strived to achieve a balance between those that have clear clinical and public health relevance, such as SUDs, versus neuropsychological functions and behaviors presumed to reflect more closely underlying neurobiological substrates.[183;184] For the 17-year follow-up, we will rely upon maternal interview, child interview, child testing, teacher report, and reviews of state and school administrative records. We will assess children's STDs and substance use with urine assays and collect saliva for DNA. We expect the entire assessment to be completed within 3.0 hours. For both mothers and children, we will use audio computer-assisted self-interview (ACASI) methods for sensitive portions of the interview; other sections will be completed by direct interview. A copy of the instrument employed at the year-12 follow-up, which will serve as the basis for the year-17 interview, is included in Appendix B. All assessment procedures will be pre-tested with 20 pilot cases. Almost all of the measures employed in this follow-up are standardized and have acceptable validity and reliability with low-income minorities. We have forgone reporting specific validity and reliability indices to save space. Tables 3 and 4 display major constructs and sources of data in our measurement design.

**TABLE 3. Constructs, Variables, and Source of Data for Assessment of Maternal Life-Course and Health**

| **Outcome Domain** | **In-person Interview** | **Admin Record** | **Biological Assay** |
|---|---|---|---|
| Subsequent pregnancies | X | | |
| Use of welfare services | X | X | |
| Substance Use and SUDs | X | | |
| Relationship with partner | X | | |
| Domestic Violence | X | | |
| Maternal Depression | X | | |
| Vulnerability Genes | | | X |

## Maternal Life-Course and Health

***Subsequent Pregnancy*.** We will assess number, timing, and outcome of subsequent pregnancies, including number of therapeutic abortions, live births, low-birth-weight newborns, and intervals between births. Our primary outcomes will consist of 2 variables: 1) the count of closely spaced subsequent births; and 2) the number of subsequent child days (density of other children in the household) over the 17-year period following the birth of the first child. The second outcome consists of the sum of numbers of days between births of each subsequent child and the first child's 17$^{th}$ birthday.

***Use of Welfare Services*.** We will interview women to assess their use of TANF, food stamps, Medicaid, WIC, supplemental social security income, and subsidized child care, and abstract Tennessee

records of TANF, food stamps, and Medicaid use. Given that over 90% of the women live in Tennessee, Tennessee records will provide reasonable estimates. Letters approving our review of these records are given in Appendix C. Our primary outcomes are total number of days that women were enrolled on food stamps and TANF/AFDC over the 17-year period following birth of the first child.

***Substance Use and SUDs.*** We will diagnose abuse and dependence on alcohol and 9 drugs or drug classes with the *Composite International Diagnostic Interview (CIDI)-Substance Abuse Module (CIDI-SAM)*, a structured, 30-to-60-minute interview designed for trained, lay interviewers. It is a descendent of the NIMH Diagnostic Interview Schedule. CIDI's reliability and validity[185;186] made it the main assessment for DSM-IV Substance Field Trials and for the National Comorbidity Study.[187] A recent study demonstrates its validity in substance-dependent, conduct-disordered adolescents.[188] We have an ACASI version for making DSM-IV Abuse/Dependence diagnoses, which may increase report accuracy and will save data entry time. We will assess women's frequency of using substances with questions from the Drug Use Screening Inventory,[189] which assesses substance use based on measures of quantity and frequency of use of cigarettes, alcohol, and illegal substances. It also includes questions on severity of problems created in 10 domains of functioning.[189] Frequency is defined as the number of times respondents used each drug in the (i) past year and (ii) past month. The quantity used per occasion will be multiplied by the frequency of days used in a given time frame (e.g., past year or past month) to give us a measure of substance exposure. A measure of exposure will be calculated for past year and past month for each substance; these 2 exposure measures will serve as primary outcomes. Our primary disorder outcome will consist of the count of SUDs summing across substances.

***Relationship with Current Partner.*** We will interview mothers regarding their current partners, lengths of their relationships, whether they are married or cohabiting, whether he is the father of index or subsequent children, his employment, education, criminal involvement, and quality of relationships with their children. We also will assess the degree to which mothers are committed to their partners, have effective communication, and experience conflict.[190] The primary outcome is the duration of their relationship counted in months; the quality of the relationship will be indexed by communication, conflict, and commitment.

***Domestic Violence.*** We will administer Straus's Conflict Tactics Scales used in earlier phases of this trial to obtain information on the degree to which mothers experienced violence from any of their partners during the 6-month period preceding the interview.[191]

***Maternal Depression.*** We will administer the Beck Depression Inventory (BDI-II) to measure depression severity.[192] Given that high scores on the BDI can be indicative of adjustment problems instead of depression, we will employ caution in interpreting these scores. Women also will complete the Beck Anxiety Inventory (BAI), a 21-item self-report check-list developed to distinguish anxiety symptoms from depression.[193]

***Parenting Behavior.*** We will employ the Loeber Youth Questionnaire to evaluate youths' perceptions of the quality of their parenting according to dimensions of parental affect and control.[194]

***Vulnerability Genes.*** We will gather saliva using Oragene technology to obtain DNA for genotyping. Our description of gathering and processing DNA and conducting genetic analyses is provided below.

***Other Maternal Measures.*** While not the focus of specific hypotheses, we will gather information on a variety of maternal characteristics that will be useful in interpreting the results of our primary analyses and that are needed for an economic analysis, which we will conduct with support from a separate application. They include measures of maternal employment by interview and review of state department of labor records; the degree to which the father of the child (if not the current partner) is involved in the life of the firstborn child; and mothers' involvement with the criminal justice system. Space limitations preclude our describing these assessments in depth; copies are in Appendix B.

**Child Assessments**

As shown in Table 4, we will evaluate the first-born children using *tests* of intellectual functioning, language, neurocognitive skills, and academic achievement; *interviews*; and *abstractions* of electronic school records and state foster care data. Moreover, for the first time in this trial, we will conduct direct assessments of adolescents' physical health, focusing on the presence of STDs and use of substances through urinalysis. We will gather DNA for genotyping, using Oragene (described below). Gathering these data will be integrated into an overall physical health assessment that includes measures of weight, height, obesity, and blood pressure. Youth will be provided counseling for all health risks identified, and in the case of STDs, they will be asked to return to the study physician to obtain confidential, one-dose treatment for detected pathogens.

***Cognitive and Language Abilities.*** We will conduct tests of intellectual and language ability to trace trajectories in these functions over time and to sort out program impact on ECF from general intelligence and language. We have chosen 2 tests that can be conducted by non-clinicians: the 4th edition of the Peabody

Picture Vocabulary Test–III (PPVT), which we administered to firstborn children at age 6; and the Matrices subtest from the Kaufman Brief Intelligence Test (KBIT–2). The Matrices is a non-verbal, visual problem-solving test with both concrete and abstract tasks. The KBIT is a brief version of the IQ measure used with firstborn children at age 6.[195]

**TABLE 4. Constructs, Variables, and Source of Data for Assessments of Children**

| Outcome Domain | In-Person Interview | Direct Assess | Admin Records | Parent Report | Teacher Report |
|---|---|---|---|---|---|
| Cognitive and Language Abilities | | X | | | |
| Executive Functions (risky decision making, impulsivity, facial recognition, verbal working memory) | | X | | | |
| Academic Achievement, including school drop out | | X | X | | |
| Behavior Problems/Mental Disorders (Depression/Anxiety) | X | | | | X |
| Substance Use and Disorders | X | X | | | |
| Sexual Behavior/Pregnancies | X | X | | | |
| Sexually Transmitted Infections | | X | | | |
| Conduct Grades, Disciplinary Actions, Attendance, and Drop Out | | | X | | |
| Violent Criminality and Gang Membership | X | | | | |
| Delinquent Peers | X | | | | |
| Arrests, Convictions, and Juvenile Detention | X | | | X | |
| Foster Care Placements | | | X | X | |
| Violence Exposure (home, school, and community) | X | | | | |
| Vulnerability Genes (MAOA, 5-HTT, COMT) | | X | | | |

***Risky Decision Making.*** We will administer a version of the *Cambridge Decision Making Task (CDMT)* adapted for adolescents[87] The CDMT was developed to dissect cognitive components of sensitivity to consequences and risk taking[196] and consistently activates the orbital portion of the prefrontal cortex in neuroimaging.[87;196] This region also modulates emotional responses to environmental and social stimuli.[197;198] Subjects are instructed to earn as many points as possible given a choice between various bet amounts associated with specific probabilities of winning. The larger reward is always associated with the least likely outcome, thus capturing the conflict inherent in risk-taking.

***Impulsivity and Distractibility.*** We will administer a computerized Continuous Performance Test (CPT) to measure ability to inhibit a prepotent impulse and shift response patterns (i.e., impulsivity). Neural responses to such tasks are most prominent in the right lateral orbitofrontal cortex as well as right superior temporal gyrus, medial orbitofrontal cortex, cingulate gyrus, and inferior parietal lobule. Subjects with greater impulsivity tend to show greater activation of paralimbic areas during response inhibition, while less impulsive individuals and those with least errors activate higher order association areas.[199] The measures produced by the impulsivity CPT include correct presses, omission errors, commission errors, and reaction time.[200]

***Facial Recognition.*** We will administer the *Facial Recognition Task.*[201] It measures the ability to identify emotional expressions in people's faces, which involves circuitry between the PFC and limbic system (particularly amygdala), and which is vulnerable to stress. The ability to recognize facial expressions properly is impaired in children and adults with externalizing disorders, such as CD, violence, and drug abuse.[202-206]

***Verbal Working Memory.*** Verbal working memory is an important component of executive functioning eroded in children with the Val/Val COMT genotype.[153] We will assess verbal working memory with the forward and backward recall of number sequences from the Wechsler Intelligence Scale for Children cognitive ability battery, which is free of need to pronounce specific words and has age-referenced norms.

***Academic Achievement.*** We will administer the reading and math subtests of the Peabody Individual Achievement Test-R-Norm-Updated (PIAT R-NU), which we administered at age 12. They have age-referenced norms. The Tennessee Comprehensive Assessment Program tests are no longer standardized according to national norms in Tennessee, so we cannot use this test as an alternative source of information on achievement. We have successfully abstracted children's academic records through grade 6 and will continue to abstract children's grade point averages in reading and math for each subsequent grade as an alternative source of information on academic achievement.

***Behavior Problems and Mental Disorders.*** We will administer the Youth Self Report (YSR) of the Child Behavior Checklist 11-18[207], as we have done at ages 2, 6, and 12, using the computerized method. The CBCL generates two broadband syndromes: internalizing problems and externalizing problems, as well as a total-problems scale. The newest version also includes a method of generating a DSM profile. Our 2 primary outcomes are depression and anxiety. The YSR will be augmented with corresponding reports from teachers

and parents. We will create both quantitative scores and dichotomous outcomes that reflect whether children fall into the clinical or borderline range of depression and anxiety. We have not hypothesized program effects on externalizing problems overall, but we do expect program effects on violent behavior (see below).

***Substance Use and SUDs***. We diagnose Abuse and Dependence on alcohol and nine other drugs or drug classes with the *CIDI-SAM,* discussed above. We will assess the frequency of cigarette, alcohol, and illegal drug use (cannabis, cocaine, crack, heroin, amphetamines, hallucinogens) using the Adolescent Version of the Drug Use Screening Inventory, which provides information on both the frequency of drug use during the past 30 days as well as information on the severity of disturbance that goes beyond categorical diagnoses.[189;208;209] We will assay urine samples to screen for cotinine, PCP, benzodiazepines, cocaine, amphetamines, THC, opiates, and barbiturates. We decided against using alcohol metabolites, given their limited sensitivity in sporadic users.[210] We have two primary substance-use outcomes: 1) the count of substance types youth report using during the past 30 days plus the count of substances for which there is a positive urine screen (a substance will get counted only once for each participant by either self-report or urine screen); and 2) a count of SUDs for each of the substances participants report using. Each of these outcomes will be expressed as low-frequency counts.

***Sexual Behaviors***. We will assess sexual behaviors for adolescents who have previously engaged in consensual sexual behaviors. Retrospective recall of sexual behaviors is stable for intervals as long as 3 months.[211;212] Given that many adolescents engage in sexual behavior on infrequent or episodic bases,[213] we have chosen an assessment interval of 3 months to maximize our ability to detect sexual behavior without compromising reliability. Information will be elicited over the past 3 months on: number of different sexual partners, types of different sexual partners (primary or casual), and partner's risk status (i.e., sex partner has other partners). Participants will estimate the number of occasions in which they engaged in protected and unprotected vaginal and anal intercourse using a format developed by St. Lawrence.[214]

***Sexually Transmitted Infections.*** We will obtain urine specimens from both boys and girls to ascertain the presence of the following treatable STDs: *N gonorrhoeae, C. trachomatis, and T vaginalis.* State-of-the-art nucleic acid amplification technology will be used for STD testing. Specifically, Becton-Dickinson's proprietary DNA amplification technology, called Strand Displacement Amplification (SDA), will test for chlamydia and gonorrhea and polymerase chain reaction (PCR) will test for trichomonas. DNA urine tests for chlamydia, gonorrhea, and trichomonas are highly sensitive and specific.[215;216] Use of the SDA/PCR assays are preferable to traditional tissue culture diagnostic methods because they are non-invasive (urine samples versus cervical/urethral swab) and can effectively be collected in non-clinical settings.[217] Dr. Caliendo's laboratory at Emory University is proficient in conducting these assays and will be used to conduct all biotesting.

Adolescents will be provided with urine collection containers and escorted to a private, secure room in which to produce the specimen. Memphis-based staff will be trained by personnel from Dr. Caliendo's laboratory in these procedures. Site study staff will decant the urine specimens to centrifuge tubes labeled with unique, anonymous, subject-sample identifiers and store the specimens in refrigerators. Subject-sample ID linkage will be maintained in confidential logs on site that will be kept in secure, locked storage. The samples will be packed in International Air Transportation Association-approved biospecimen boxes and shipped via Federal Express overnight delivery to Dr. Caliendo's laboratory. The laboratory will process the specimens and send the results to the Rochester data processing center within 1 week.

Youth who test positive for an STD pathogen (chlamydia, gonorrhea, or trichomonas) will be offered directly observed treatment, single session (DOT-SS) therapeutic regimen, unless epidemiology of the pathogens in Memphis indicate other treatment regimens. We will treat infected adolescents at no charge with the following antimicrobials,[218] each of which constitutes a single session therapeutic regimen: N. Gonorrhea – Ciprofloxacin (Cipro™); C. Trachomatis–Azithromcyin (Zithromax™); T. Vaginalis–Metronidazole (Flagyl™). Chlamydia and gonorrhea are reportable infections and will be reported by clinic staff to the TN State Department of Health. Our rationale for basing treatment on these DNA amplification tests is: 1) the test has been extensively evaluated in several research settings and shown to have superior sensitivity and specificity to existing diagnostic methods; 2) it is clinically and ethically contradictory to offer retesting with less sensitive methods; 3) STD experts are increasingly concerned about the importance of trichomonas as an STD, increasing the importance of highly accurate diagnosis.[219-221] An adolescent medicine physician on contract with the study office in Memphis will be designated to administer medication, review symptoms, and provide standard STD counseling. These standard activities treat the index STD. Other types of STD-related care (e.g., HIV counseling/testing; syphilis screening) are not study outcomes and will be conducted at the discretion of the participant's clinician. Despite appropriate medical care, there is high recurrence of STDs (nearly 40%).[222;223]

Study staff cannot request identifying information about the adolescents' sex partners. However, in the Parental Consent and Adolescent Assent, we will explicitly stipulate that per state statutes, we are required to

notify the County Health Department of all reportable STDs. The County Health Department is legally responsible for and has a mechanism in place to initiate partner tracing for reportable STDs and provide treatment to partners. However, as part of this study, we will instruct adolescents treated for STDs to refer their sexual partners for evaluation, testing, and treatment at a local health department, clinic, or physician's office. To facilitate this process, study staff will provide adolescents with a brochure containing the phone numbers and contact information for the local health department or STD clinics.

***Pregnancies.*** We will assess the number and outcomes of pregnancies (miscarriage, therapeutic abortion, still birth, live birth) for both males and females, focusing on the timing and number of pregnancies. 49% of these youths' mothers were pregnant with the target child by age 17. We have estimated that 30% of this sample will have been pregnant by age 17. The primary outcome will be the count of pregnancies.

***Risk for HIV Infection.*** We will examine the degree to which data on SUDs, sexual risk behaviors, STIs, and pregnancies can be aggregated into reliable risk indices that characterize HIV risk behaviors.

***Conduct Grades, Disciplinary Actions, Attendance, and Drop Out.*** The Memphis City School system records conduct grades and disciplinary actions for children. Disciplinary actions include being sent to the principal's office, suspensions, and expulsions. Conduct grades are given for each marking period and provide a more refined, age-sensitive, and objective characterization of the children's adaptive behavior at school than we could obtain if we had to rely only on disciplinary records or teacher reports. We will assess these same outcomes for children who attend school in other districts. The primary outcomes will consist of: 1) the count of failed conduct grades from 1$^{st}$ to their most recently completed grade; 2) the count of suspensions and expulsions. Analyses will adjust for varying years of education completed. A second domain of functioning will consist of the school attendance rates (or truancy) and whether children drop out of school by age 17.

***Violent Criminality and Gang Membership.*** Following the National Youth Survey,[224] youth will be interviewed to assess whether they had engaged in the following categories of criminal behavior during the year prior to the interview: felony assault, felony theft, robbery, fraud, minor assault, minor theft, or vandalism/damaged property. We will examine the data to determine whether they can be aggregated into a scale of interpersonal violent offending based upon the count of specific violent behaviors youth report having engaged in during the 12 months preceding the interview (aggravated assault, sexual assault, gang fights, strong-armed students, strong-armed teachers, strong-armed others, hit teacher, hit parents, hit students).

***Delinquent Peers.*** We will assess the degree to which respondents are involved with deviant peers by asking the youth to answer each question from the National Youth Survey regarding the degree to which their friends engage in the delinquent/criminal behaviors.

***Arrests, Convictions, and Juvenile Detention.*** We will assess by interview the number of times the children were arrested, convicted, and sent to juvenile detention, and the offenses that led to the arrests. Using a life history calendar, youth will report the dates of arrests and the reasons. The primary outcome will consist of the count of arrests for violent offenses. Secondary outcomes will consist of the counts of a) arrests, b) convictions, and c) times sent to juvenile detention.

***Foster Care Placements.*** We have approval to review child welfare records after 2001. Abstraction of records after this date will improve validity (earlier records are incomplete) and reduce surveillance bias on families' involvement with child welfare, as families will have ended program participation years earlier.[225]

***Violence Exposure.*** We will administer the Screen for Adolescent Violence Exposure (SAVE), an instrument developed with inner-city African-American youth to assess 3 types of violence (Traumatic Violence, Indirect Violence, and Physical/Verbal Abuse) experienced in the community, home, and school.[226]

***Vulnerability Genes.*** As with the mothers, we will gather saliva using Oragene technology to obtain DNA. Our description of gathering and processing DNA and conducting genetic analyses is provided below.

**Contextual Factors**

We will assess the children's social contexts in their homes, schools, and neighborhoods.

***School.*** We will employ public school administrative data to assess characteristics of schools that children attend that are likely to affect their academic and behavioral adjustment: 1) percent of children who receive free lunches, 2) average class size, 3) average standardized achievement test scores for students; and 4) percent of student population with suspensions and expulsions.

***Neighborhood.*** We employ block-level Census data (e.g., % poverty, unemployment, female-headed households, households receiving public assistance) to create variables that characterize neighborhood adversity.[227-229] We will code the block-group data from the census tract that corresponds to each of the neighborhoods in which the children live at the time of their 17-year assessment.

**Genotyping MAOA, *5-HTT, and COMT***

Under the direction of Dr. Mary-Anne Enoch, the Laboratory of Neurogenetics at NIAAA will conduct genotyping of the 3 genes hypothesized to interact with child abuse and neglect and life stress. Participant mothers and children will provide saliva, which will be processed using Oragene[230] and sent to NIAAA. At the NIAAA lab, genomic DNA will be extracted and concentrations normalized. Genotyping for 5-HTT will be done by methods described in Hu et al.[144] and MAOA-LPR by methods describe in Ducci et al.[231] MAOA-LPR will be done only for children. Genotyping of the COMT Val158Met polymorphism will be done in conjunction with genotyping for 186 ancestry-informative markers (AIMS) using a large-scale addictions array tool for identifying vulnerability genes in addiction and psychiatric disorders. The addictions array is a tool developed within the Laboratory of Neurogenetics and has been validated in preliminary analyses using four distinct ethnic populations; Finnish Caucasians, African Americans, Han Chinese and Native Americans. Accuracy as determined by duplication was found to be >99.99 for all populations. Using the program Structure2.0[3] the AIMS were able to differentiate all four ethnic groups and to quantify the relative degrees of admixture. Analysis will be performed by the Illumina GoldenGate assay protocols, using a TECAN liquid handling robot, using sentrix 96-sample format arrays. Imaging will be performed using an Illumina 500GX Beadstation and genotype data analyzed using Beadstudio 2.0 software (Illumina). Use of this array allows for determination of the COMT polymorphism and AIMs genotyping at the lowest possible cost per sample.

In the analyses reported below, we will examine AIMS allele frequencies by treatment condition and gender to ensure that the estimation of treatment effect by genotype subclasses are not confounded by differences in population admixtures. If AIMS do vary across treatments or within treatment by subgroup classifications, statistical adjustments will be made for these differences in population stratification following procedures specified by Pritchard and colleagues.[3]

***Promoter Region of the Serotonin Transporter (5-HTTLPR).*** The promoter region of the serotonin transporter gene (*SLC6A4*) will be used to characterize genetic vulnerability to depression and to test whether 5-HTT gene variation moderates the influence of child maltreatment and life stress on depression. The promoter activity of the 5-HTT gene, located on 17q11.2, is modified by sequence elements within the proximal 5' regulatory region, designated the 5-HTT gene-linked polymorphic region (5-HTTLPR). The short ("S") allele in 5-HTTLPR is associated with lower transcriptional efficiency of the promoter compared with the long ("L") allele. An additional functional allele has been discovered in the HTTLPR locus: an A to G substitution in the first of the 2 extra repeats defining the L allele.[144] This polymorphism accounts for more of the inter-individual variation in 5-HTT expression.[144] The 3 alleles are S, $L_A$ and $L_G$; their frequencies are given in Table 5. The $L_G$ and S alleles are functionally equivalent: both cause reduction of expression. On a functional basis, S and $L_G$ can be grouped for genetic analyses, yielding 3 genotypes, low/low, low/high and high/high. Frequencies of these genotypes are approximately 1:2:1 in African-Americans and Caucasians. Those individuals with either S/S, $S/L_G$, or $L_G/L_G$ genotypes will be grouped for analysis to reflect the vulnerable segment of the sample. Details of the methods used for genotyping are given in Hu et al.[144]

**Table 5. Distribution of HTTLPR Genotype and Allele Frequencies in Several Populations**

| Population | N | Frequency of Genotype | | | | | | Freq. of Allele | | |
|---|---|---|---|---|---|---|---|---|---|---|
| | | SS | $SL_A$ | $SL_G$ | $L_AL_A$ | $L_AL_G$ | $L_GL_G$ | S | $L_A$ | $L_G$ |
| U.S. whites group 1 | 297 | .16 | .33 | .09 | .26 | .14 | .03 | .37 | .49 | .14 |
| U.S. whites group 2 | 286 | .12 | .37 | .08 | .22 | .18 | .02 | .35 | .50 | .15 |
| **African-American** | **624** | **.07** | **.25** | **.12** | **.27** | **.23** | **.06** | **.25** | **.51** | **.24** |

**From: Hu, et al., (2006). *American Journal of Human Genetics,78*:819.**

***Monoamine Oxidase A (MAOA).*** A well-characterized variable number tandem repeat (VNTR) polymorphism exists at the promoter of the *MAOA* gene, which affects expression.[145] The polymorphism (MAOA-LPR), located 1.2 kb upstream of the MAOA coding sequence, consists of a 30-bp repeated sequence present in 3, 3.5, 4, or 5 copies. The polymorphism displays significant variations in allele frequencies across ethnic groups. The polymorphism has been shown to affect transcriptional activity of the MAOA gene promoter by gene fusion and transfection experiments involving 3 different cell types. Alleles with 3.5 or 4 copies of the repeat sequence are transcribed 2-10 times more efficiently than those with 3 or 5 copies of the repeat, suggesting an optimal length for the regulatory region. Transcription efficiency is not yet known for the 2 and 5 copy repeats. Primer sequences and PCR conditions are described in Ducci et al.[231] Table 6 shows frequencies for these alleles in African-Americans; note that alleles with 3 (low activity) and 4 (high activity) copies are much more frequently occurring. Alleles will be grouped as either low or high activity for analysis.

**Table 6. Allele frequencies for the MAOA-VNTR among African-American males and females (courtesy of F. Ducci, 2007).**

| | | Allele frequencies |
|---|---|---|

| Population | N | 2 | 3 | 3.5 | 4 | 5 |
|---|---|---|---|---|---|---|
| Males | 620 | 0.05 | 0.50 | 0.001 | 0.45 | 0.003 |
| Females | 340 | 0.07 | 0.47 | 0 | 0.45 | 0.004 |

***Catechol-O-Methyltransferase (COMT).*** Catechol-O-Methyltransferase (COMT) is largely responsible for metabolism of DA and NE in human pre-frontal cortex. A common COMT Val158Met polymorphism is responsible for a 3 to 4 fold variation in enzyme activity.[150] Table 7 shows the allele frequencies and genotypes for the COMT polymorphism among African-American and white control subjects in a population-based study of breast cancer in North Carolina.

**Table 7. COMT Allele frequencies and genotypes for whites and blacks in North Carolina**

| | | Genotype | | | Allele Frequency | |
|---|---|---|---|---|---|---|
| **Population** | N | COMT-H/H | COMT-H/L | COMT-L/L | COMT-H | COMT-L |
| African-Americans | 263 | 0.42 | 0.45 | 0.13 | 0.65 | 0.35 |
| Whites | 379 | 0.22 | 0.50 | 0.28 | 0.47 | 0.53 |

**Statistical Models and Methods of Analysis**

The primary analyses will make use of general linear model methods and their extensions. The focus will be on (1) full model specification to account for all sources of variation, and a full examination of interactions among model factors, including examination of homogeneity of regressions to understand interactions between classification effects and covariates; (2) generalized models to analyze dichotomous outcomes with binomial error distributions and count data assumed to have Poisson or negative binomial distributions (using Poisson models when Poisson deviances are near 1); (3) mixed models (including repeated measures and growth-curve models) to account for correlated data and to examine differences in change over time; (4) latent class analysis[232] and latent transition analysis[233] to examine the assessment of children's transition over time into different patterns of substance abuse and dependence and psychopathology; (5) mixed models to examine contextual effects of neighborhoods and schools; (6) semiparametric methods to model relations between variables without requiring a specific functional form; (7) structural equation models to examine causal processes, including the direct and indirect effects of treatment on outcomes such as SUDs and the mediational role of density of subsequent children. Generalized models will be analyzed in mixed model form when random factors are included. So also will be the structural models to the extent possible. Methods as described in, for example, Searle,[234] Henderson and Henderson,[235] Henderson,[236;237] Laird and Ware,[238] and Searle, Casella, and McCulloch[239] will be used in the analysis of the mixed general linear models; for semiparametric analyses, Hastie and Tibshirani,[240] and Ruppert, Wand, and Carroll;[241] for generalized linear models, McCullough and Nelder[242] and Baker and Nelder[243] and for generalized mixed models Schall[244] and Wolfinger and O'Connell;[245] and for structural models Bolen,[246] Schmidt,[247] and Goldberger[248] and for testing indirect effects and moderation Henderson and Ceci.[249]

***Missing Data.*** While missing data in this trial have been remarkably low and we expect participant attrition to be even lower than we achieved at the year-12 follow-up, we are sensitive to the potential pitfalls of only analyzing cases with complete data. We have complete data on all relevant background characteristics. Our primary strategy is to minimize attrition to greatest extent possible. We will also compare our analyses to the same analyses employing multiple imputation.[250] For the multiple imputations, we will generally utilize a Markov Chain Monte Carlo method to impute enough data to have a monotone missing data pattern. Once we have a monotone missing data pattern, we will employ imputation approaches appropriate for the type of data (continuous, dichotomous, or low frequency count data). We will perform sensitivity analyses to ensure that results are similar under various assumptions. Ultimately, we will determine whether imputation is valid and has sufficient added value with our data to justify the complexity involved in reporting these analyses.

***Data Reduction.*** To simplify our initial analyses, the dependent, intervening, and exogenous variables will be summarized in so far as possible by composites derived from principal components analysis or according to a priori scales. As indicated above, reliability and validity work either has been or will be carried out on these scales. Through the discussion that follows, use of the terms "dependent variable" and "intervening variable" will include these summary scores or latent variables.

***Non-Normal Distributions.*** Full attention will be given to the distributional properties of variables and to regression diagnostics. For low-frequency outcomes that have Poisson deviances around 1, we will employ Poisson error assumptions. Otherwise, we typically will employ negative binomial error assumptions, and make adjustments for outlying data points as necessary.

***Examination of Mediation.*** We will examine mediation in models with simultaneous equations, testing

the significance of direct and indirect effects and their equality. Testing will be by the delta method[249] or bootstrapping. While tests involving indirect effects are of primary interest, we will in some cases carry out tests reflecting Mackinnon's recommendations[251] in which we regress the outcome on treatment and mediator, and the mediator on treatment. The paths from intervention to mediator and from mediator to outcome must each be statistically significant by this criterion, as must the indirect effect. If the path from intervention to outcome approaches zero, full mediation is suggested. Partial mediation is suggested to the extent that intervention still predicts outcome with the mediator simultaneously entered. These first-stage tests also result from the model estimation used for testing the indirect effect. In that model estimation, we use full information methods (e.g., FIML) unless there is clear indication that OLS is adequate. Henderson and Ceci[249] describe delta-test methods for moderation and mediation, which we will use.

***Examination of Hardy-Weinberg Equilibrium (HWE)*** ***Examination of Hardy-Weinberg Equilibrium (HWE ).*** We will calculate allele frequencies for each polymorphism examined in this study (5-HTTLPR, MAOA-LPR, COMT Val158Met) to determine whether the sample is in HWE. Given the relatively small sample sizes involved in these calculations, we will rely upon absolute deviations from HWE in addition to formal statistical significance in making determinations as to whether the polymorphisms are in HWE.[2]

**Overview of Primary Statistical Models**

The primary classification factors for examination in our models, as determined by our hypotheses and earlier work, include treatments (Control vs. Nurse Visited - T), maternal psychological resources (low versus high – P), and sex of child (S). The most likely covariates are indices of household poverty, neighborhood disorganization at the time of registration, and maternal beliefs associated with child abuse. Given that we are adding genetic data to our models, we also will examine AIMs to determine whether the T x P x S terms are confounded by different population admixtures. If such confounding is detected, we will employ statistical adjustments for differences in population stratification.[3]

An important focus of analysis will be on the contextual effects of neighborhoods and their moderation of program effects. We plan to include block groups (a proxy for neighborhoods) as levels of random factors in certain analytic models. In these models, we will examine 3 types of covariates: (1) covariates associated with the usual fixed regressions measured at the level of children or families; for example, family income, maternal locus of control, and other background sociodemographic variables; (2) covariate regressions specific to neighborhoods in addition to the conventional overall fixed regressions; these group regressions, from a sampling standpoint, must have a random distribution because they are associated with the effect for groups, the levels of which are random;[236] (3) covariates that provide information about neighborhoods (e.g., the neighborhood adversity index); these are measured only at the level of neighborhoods and have higher-level regressions.[235;236] By looking at regressions of these 3 types, we will be able to sort out much of the complexity of contextual versus individual effects. The homogeneity of each of these types of regressions will be tested for treatments and the other fixed factors in our core models.

In the examination of school outcomes, repeated measures for subject area and for grade will be added to the model. In this model, we can test differences by subject and grade and interactions between them and among other model variables. In models with data over time at a number of assessment points, growth curve models can be used. In these models, we will estimate simultaneous regions of statistical significance to examine ranges of treatment effects. These models may be nonlinear, parametric, or semiparametric.

***Inclusion of Gene Activity Classification Factors.*** Some of our models will include classification factors for functional classifications (activity variants) of the polymorphisms hypothesized to moderate program impact in this trial (5-HTTLPR, MAOA-LPR, and COMT Val158Met); we are not interested in genotypes per se, but rather functional classes that group genotypes by activity level (low – L , high - H or intermediate - I). In the case of 5-HTTLPR, $L_G$ and S alleles are both low-activity variants that lead to low expression; from the analysis standpoint, the low-activity genotypes ($L_G/L_G$, S/S, and $L_G$/S) will be grouped into the low-activity class; the high activity genotypes ($L_A/L_A$,) into a high-activity class; and the remaining genotypes ($L_A/L_G$ and $L_A$/S) grouped into an intermediate class. We will use a similar approach with MAOA-LPR and COMT Val158Met. In the case of the X-linked MAOA-LPR, we are grouping males into high and low expression allele groups and females (in whom there is the likelihood of no X inactivation[137;148]) into low, high and intermediate expression groups for purposes of analysis.

**Table 8. Estimated Subclass Sizes for the Cross-Classifications of Treatments, Child Gender, and *5-HTTLPR* , *MAOA-LPR,* and *COMT* Activity Subgroups**

| Gene Activity Class | Treatment 2 Comparison (N=416) | | Treatment 4–Prenatal & Infancy Home Visitation (N=184) | |
|---|---|---|---|---|
| | Males n=208 | Females n=208 | Males n=92 | Females n=92 |
| 5-HTT - L | n=52 | n=52 | n=24 | n=24 |
| 5-HTT - I | n=100 | n=100 | n=44 | n=44 |
| 5-HTT - H | n=56 | n=56 | n=24 | n=24 |
| MAOA - L | n=104 | n=46 | n=46 | n=20 |
| MAOA - I | - | n=88 | - | n=38 |
| MAOA - H | n=94 | n=42 | n=42 | n=20 |
| COMT - L | n=28 | n=28 | n=12 | n=12 |
| COMT - I | n=94 | n=94 | n=42 | n=42 |
| COMT - H | n=88 | n=88 | n=38 | n=38 |

Note: MAOA 2 and 5 copies are not included because their activity is not known.

Table 8 shows estimates of subclass sizes for the gene activity subgroups within the gender and treatment factors. The shaded rows indicate the vulnerability subgroups for which hypotheses are framed. Given that complete cross-classification of treatments (T), gender (S), and genetic activity groups (G) will produce small subclasses, we will not obtain stable estimates when analyses are conducted within complete cross-classifications of T, S, and G. We thus will have to make choices about how to handle child gender, that is, whether to include all interactions or gender only as a classification factor without interactions. In the discussion of the smallest detectable treatment difference for genetically vulnerable subgroups given in Table 10 below, we have averaged across child gender for estimations of smallest detectable program effects, for the 5-HTTLPR and COMT risk factors. This produces larger subclasses for typical analyses than implied in Table 8.

**Statistical Power/Smallest Detectable Differences for Proposed Follow-Up**

Given that sample size is fixed, we show below calculations of the smallest detectable treatment-control differences for the next phase of follow-up. Calculations for representative outcomes were carried out with the following assumptions: a Type I error of .05, Type II error of .20 (power of .80), 2-tailed tests, 10 percent of the variance accounted for by other terms in the model for normally distributed variables and N=600. Disproportionate sample sizes in Control and Nurse-Visited groups are taken into account.

***Smallest Detectable Program Effects without Considering Genetic Vulnerability.*** Table 9 displays the smallest detectable treatment differences without sub-classification of the sample by genetic vulnerability. The study has sufficient power to detect small to moderate effect sizes for normally distributed variables for tests of treatment main effects (.24 SD) and moderate effect sizes (.34 SD) for tests conditioned by a single factor that is evenly distributed between two levels (e.g., child sex, maternal psychological resources). For outcomes that are dichotomous, our calculations of smallest detectable treatment differences are calculated in the binomial logistic-linear model and shown for a range of base rates likely to be found in our outcomes, such as substance use, SUDs, and STIs. It is important to note that we have detected program effects of this magnitude in earlier phases of the trial and that it is expected that some program effects will emerge even more powerfully as the children enter new periods of risk and development.

**Table 9. Smallest detectable treatment effect for dependent variables for treatment main effects and for subgroups defined by maternal psychological resources or gender**

| Base Rate for OR | Smallest Detectable Treatment Contrasts for ORs | Smallest Detectable Treatment Contrasts for ORs |
|---|---|---|
| | **Whole Sample N=600** | **One Half of Sample at Greater Risk (e.g., Low-Resource Mothers or Boys) N=300** |
| 10% | .33 | .14 |
| 20% | .49 | .33 |
| 30% | .55 | .42 |
| 40% | .59 | .46 |
| | **Smallest Detectable Treatment Contrasts for ESs** | **Smallest Detectable Treatment Contrasts for ESs** |
| | .24 | .33 |

Note OR = $T_4$ odds/$T_2$ odds; ES = ($T_4$ mean *minus* $T_2$ mean)/pooled standard deviation

***Smallest Detectable Program Effects for Genetically Vulnerable Subgroups.*** We conducted separate analyses that estimate a range of program effects that can be detected reliably within gene activity subclasses. Table 10 shows smallest detectable treatment differences in odds ratios and effect sizes (expressed in SD units) for the genetically vulnerable subgroups hypothesized to moderate program impact. For dichotomous outcomes, we show estimates for untreated outcome base rates ranging from .20 - .40; these baseline rates are likely to include outcomes like STIs, pregnancy, depression, and rates of school drop-out. The bottom part of Table 10 shows the smallest detectable effect sizes expressed in SD units for these genetically vulnerable subgroups.

**Table 10. Smallest Detectable Effect (OR or ES) for Treatment Contrasts within Genetic Subgroup**

| Base Rate for OR | Smallest Detectable Treatment Contrasts for ORs within Genetic Subgroups | | |
|---|---|---|---|
| | **5-HTT – L** | **MAOA – L*** | **COMT - H** |
| 20% | .16 | .15 | .28 |
| 30% | .26 | .25 | .37 |
| 40% | .32 | .21 | .42 |
| | **Smallest Detectable Treatment Contrasts for ESs within Genetic Subgroups** | | |
| | .47 | .47 | .37 |

Note OR = $T_4$ odds/$T_2$ odds; ES = $T_4$ mean *minus* $T_2$ mean/pooled standard deviation; * Males only

Estimates for the MAOA vulnerability subgroup are for males only. While the program will have to produce very large effects on low-frequency outcomes, i.e., with base rates at or below 20%, the program has produced Odds Ratios and Effect Sizes nearly this large at earlier phases of the trial for vulnerable subgroups. While treatment effects will need to be moderate to large to be detected, it is possible that the program effects we have observed to date mask effects that are even larger for these genetically vulnerable groups, and will be detectable in these preliminary analyses.

**Tests of Hypotheses**

***Hypothesis 1 (primary): The program will continue to improve maternal life-course, especially for mothers with high psychological resources.*** We will test Hypothesis 1 by examining each of the primary maternal life-course outcomes (count of short (< 2-yr.) inter-birth intervals, use of TANF and food stamps and duration of partner relations) in our primary model, which includes a factor for T and P, the interaction between these classification factors (T x P), and a set of covariates. We will examine the treatment–control difference summing across levels of maternal psychological resources, and will conduct planned contrasts for women in the high-resource group. Most variables will be examined in the general linear model. Use of TANF and food stamps, and duration of partner relations are all essentially normally distributed. They will be measured at 4 or more time points, permitting repeated measures or growth-curve models. We expect that slopes of growth in these outcomes will be different for intervention and control groups and that these differences will be more pronounced for mothers with high psychological resources. The range of significant differences will be estimated by simultaneous regions of significance. For the count of short inter-birth intervals, we will employ a log-linear model, assuming negative binomial error.

***Hypothesis 2 (secondary). The program will reduce maternal SUDs and depression, effects that will be more pronounced in a) low-resource mothers and b) those living in the most disadvantaged neighborhoods at registration.*** We will examine SUDs and depression by examining planned contrasts within the classification structure specified for Hypothesis 1, using generalized linear models, logistic in the case of depression and assuming either negative binomial error for counts of SUDs. We will test treatment–control differences both overall and for low-resource mothers. We will examine the significance of the T x P interaction term, but also examine the size of treatment effects for high and low-resource subgroups to see if the low-resource group consistently has greater treatment–control differences. To examine whether the program effect is intensified among women in the highest risk neighborhoods, we will conduct homogeneity of regression analyses, fitting regressions of these outcomes on the neighborhood adversity variable, specified separately by treatment and psychological resource subgroups, and testing treatment differences in their slopes and intercepts for the sample overall, as well as for mothers in the low-psychological-resource group.

***Hypothesis 3a–d (primary): The program will improve the health and development of children, who will exhibit: a) superior cognitive, language, executive, and academic functioning; b) less depression and anxiety; c) fewer failed conduct grades and school disciplinary actions, and d) less violent behavior and gang membership, and fewer arrests, juvenile detentions, and convictions – especially for crimes involving interpersonal violence.*** We will examine this hypothesis in the model used to test hypotheses 1 and 2, after adding child sex (S) as a classification factor. We will test hypotheses 3a–d by examining each of child outcome in the saturated TxPxS (2x2x2) model, including covariates. In our first-level analysis, we will examine treatment–control differences averaging across levels of maternal psychological resources and child sex. Outcomes that have essentially normal distributions (e.g., cognitive and academic outcomes) will be examined in the general linear model. Those that have low-frequency count distributions (e.g., counts of failed conduct grades) will be examined in the generalized model. For very sparsely distributed outcomes, we may need to drop certain model terms, such as 3-way interactions or nonsignificant covariates to increase model stability. We will retain model terms with highest levels of significance and those central to the hypothesis. Finally, we will examine outcomes that have binomial distributions in the fully specified logistic model. In analysis of executive cognitive functions, we will control statistically for KBIT matrices scores and PPVT to discern the impact of the program on executive functions independent of general intelligence and language. Some outcomes, such as GPAs, are likely to have multiple measures for each child at different grades, and the model may include additional repeated measures factors for grade and subject area.

***Hypothesis 4 a–b (primary): The program will reduce children's risk for HIV exposure, including, reduced: a) use of substances and SUDs, b) risky sexual behaviors, c) STIs, and d) pregnancies.*** We will employ nearly identical analytic methods for testing hypothesis 4 as we employ for testing hypothesis 3. The primary outcome(s) for HIV risk will consist of indices that we will create to quantify HIV risk exposure.

***Hypothesis 5 (primary): Program effects on children will be more pronounced for a) males, b) those born to low-resource mothers, and c) those who lived in the most disadvantaged neighborhoods at registration.*** We will test the hypothesized conditional effects in the core model (TxPxS with covariates). Planned contrasts for children will focus the treatment contrast on children born to low-resource mothers. We will test whether program effects are greater for males by examining treatment–control differences within the male subgroup. We will examine the conditional effects of neighborhood adversity by fitting regressions of dependent variables on neighborhood adversity separately for the groups defined by treatments and psychological resources and conducting tests to determine the homogeneity of regressions. We hypothesize that the slopes of the regressions will be significantly different, with the control group children and especially males succumbing to the adverse effects of neighborhood adversity to a greater degree than those in the nurse-visited groups. These differences will be expressed as significant treatment differences in the slopes of the regressions, with treatment-control differences accentuated in more disadvantaged neighborhoods.

***Hypothesis 6* (secondary)*: Program effects on mothers and children will be more pronounced for those with genetic vulnerabilities:*** *a) Effects on youth depression and anxiety will be greater for those with the low-activity 5-HTTLPR genotypes (S/S, $L_G/L_G$, $S/L_G$) compared to those with the high-activity genotypes ($L_A/L_A$); effects on these outcomes will be of intermediate magnitude for those with intermediate activity genotypes ($S/L_A$).* We will test this hypothesis by adding a 3-level 5-HTT genetic vulnerability factor to the statistical model and examining treatment–control differences within levels of genetic risk. *b) Effects on youth violent antisocial behavior, SUDS, and risky sexual behavior, including STIs and pregnancies, will be more pronounced among males with the MAOA-LPR low-activity alleles and among both males and females with the Val/Val (H/H) COMT genotype.* We will repeat the analysis of child outcomes, substituting first a factor that classifies the sample into groups of MAOA alleles that confer high versus low activity, focusing the treatment contrast on males within the low-MAOA category; we will repeat this analysis for both males and females, substituting the COMT genotype as the genetic vulnerability factor, focusing the treatment contrast on the high-activity subgroup (Val/Val). *c) Effects on maternal SUDs will be concentrated among those mothers with the Val/Val COMT genotype.* In testing this hypothesis, we again will add the COMT genotype (H/H, H/L, LL) to our standard model and repeat the analysis of maternal SUDs, focusing on our planned treatment contrast on the H/H subgroup. *d) Effects on child outcomes will be more pronounced among children born to mothers with either: 1) the low-activity 5-HTTLPR genotypes; or 2) 2 copies of the high-activity COMT Val158 alleles (COMT-H).* In testing this hypothesis, we again will add the 5-HTTLPR genetic risk factor to our standard models and repeat the analysis of child outcomes, with planned contrasts focusing on treatment–control differences within the low-activity 5-HTTLPR subgroup defined by mothers' having either high- low- or intermediate activity classifications. Given limited statistical power to examine treatment contrasts within any one of these subgroups alone, we also will create a maternal genetic risk factor based upon whether mothers possess any one of these risk genotypes and then re-run the analysis of treatment-control differences focusing on planned comparisons within the high genetic risk factor defined by mothers possessing either one of these 2 risk genotypes.

***Hypothesis 7 (Secondary): Program effects on adolescent functioning will be explained by its improvement in prenatal health, early care of the child, maternal life-course, and earlier child functioning.*** Tests of mediation will follow the theoretical model of Figure 1 and use the methods described above. To illustrate, we have hypothesized that nurse visitation will have an indirect effect on adolescent academic performance through its effect on maternal-life course. To test one aspect of this hypothesis, we will regress academic performance on treatment condition and density of subsequent children born to the mother; we will also regress density of subsequent children on intervention condition. If the paths from intervention to density and from density to academic performance are both significant, we will have met the joint significance test criterion. We will next calculate the indirect effect, based on these same two path coefficients, and compare the resultant z' to Mackinnon's table of critical values. If the z' is equal to or higher than the appropriate critical value, we will conclude that density of subsequent children mediates the effect of nurse visitation on academic outcomes.

**Challenges in the Conduct of This Work**

1. **Limited Statistical Power for Detection of Treatment Effects for Genetically Vulnerable Groups in Context of Environmental Stress.** The proposed analyses are exploratory and cost minimal, given that the Laboratory of Neurogenetics at NIAAA is conducting this work for the cost of supplies and processing.
2. **Limitations of Single Polymorphism Candidate Gene Approaches.** The study of complex diseases with single polymorphisms in candidate genes has been questioned. Some have recommended haplotype analyses and genome wide scans as alternative approaches. While those approaches are quite promising, they are not feasible within the kind of randomized controlled trial we are conducting, given limits of sample size. It is worth noting that a recent replication of the Caspi MAOA-LPR polymorphisms x environment interaction result among females was essentially reproduced using not only the MAOA-LPR polymorphism, but with analyses of alleles and haplotypes. Our exploratory analyses thus show promise and are a reasonable approach to adding genetic data to this longitudinal trial.
3. **Multiple Comparisons.** If we were to make Bonferroni-like adjustments to our statistical tests in this trial, the cost of conducting such studies with adequate power would be prohibitive, given the large number of outcomes examined over time.[252] Our approach has been to make specific hypotheses, separate primary and secondary outcomes, and to require replication before giving credence to any one finding.[252] This is a sensible strategy that in the long run minimizes costs. For exploratory analyses of outcomes where this kind of pre-hoc rigor is not applied, we will make Bonferroni-like adjustments for multiplicity.
4. **Increased Difficulty in Tracing Sample.** We have been extraordinarily successful in retaining this sample for all previous follow-ups. Given that some children will have dropped out of school and the mothers and children will increasingly rely upon cell phones rather than land-lines, tracing the sample will be more difficult than it has been in the past. The strong relationship established by our Memphis-based data-gathering team, their relationships with other contact people and the Memphis community, and the increased honorarium will increase their ability to maintain this sample.

**Work Plan and Time Line**

As indicated in Table 11, conducting the interviews, bio-assays, reviews of state and school archived data and processing of data will last 48 months. Within 6 months after the completion of the interviews, we will have all interview data processed. Once interview data are processed, we will have permission to review school and state records. Within 6 months after completing the processing of the interview data, we will have all school and state administrative records abstracted and downloaded. The processing of those records will require another 6 months. Finally, within 6 months of completing the interviewing and testing, we will have the DNA processed and genotypes specified. We will have 15 months to analyze the data and 9 months to prepare reports and publications based upon this source of information. We will have 6 months to analyze the school and state administrative data, and 6 months to prepare publications and reports on data from these sources.

**Table 11. Time Line for Completion of Research**

| | Study Year and Quarter | | | | | | | | | | | | | | | | | | | |
|---|---|---|---|---|---|---|---|---|---|---|---|---|---|---|---|---|---|---|---|---|
| | Year 1 | | | | Year 2 | | | | Year 3 | | | | Year 4 | | | | Year 5 | | | |
| Activities | 01 | 02 | 03 | 04 | 05 | 06 | 07 | 08 | 09 | 10 | 11 | 12 | 13 | 14 | 15 | 16 | 17 | 18 | 19 | 20 |
| Trace Sample | X | X | X | X | X | X | X | X | X | | | | | | | | | | | |
| Conduct interviews and tests | X | X | X | X | X | X | X | X | X | X | | | | | | | | | | |
| Process interview and testing data | | X | X | X | X | X | X | X | X | X | X | X | | | | | | | | |
| Analyze interview and testing data | | | X | X | X | X | X | X | X | X | X | X | X | X | X | X | X | | | |
| Prepare reports on interview / test data | | | | | | | | | | | | | | | | | | X | X | X |
| Process genetics data | | X | X | X | X | X | X | X | X | X | X | X | X | X | | | | | | |
| Analyze genetics data and Tx by g | | | | | | | | | | | | | | X | X | X | X | X | X | X |
| Download school & state data | | | X | X | X | X | X | X | X | X | X | X | X | X | | | | | | |
| Process state and school records | | | | X | X | X | X | X | X | X | X | X | X | X | X | X | | | | |
| Analyze state & school records | | | | | | | | | | | | | | | | | X | X | | |
| Prepare state & school reports | | | | | | | | | | | | | | | | | | X | X | X |

## E. Human Subjects

### Risks to the Subjects

**Human Subjects Involvement and Characteristics.** This study is a longitudinal follow-up of healthy 670 women (ages 25-46 at the proposed follow-up) and their firstborn children (around age 17, n=670) who have participated in a randomized trial of prenatal and infancy home visiting by nurses in Memphis, Tennessee since 1988. The study will be conducted when the firstborn children complete eleventh grade, or around their 17$^{th}$ birthday. It is designed to examine whether highly promising long-term effects found for nurse home visiting with a white sample living in a semi-rural area (Elmira, NY) will be replicated with a primarily black sample living in a major urban area (Memphis, TN). This is a preventive intervention trial focused at earlier phases on pregnant women and young children. The proposed work involves assessing the women and children 17 years after the women delivered their first child.

**Sources of Materials.** Sources of data include interviews and spit samples with the mothers; interviews, urine assays, spit samples, physical exam, and assessments with children; state and federal administrative records identifying women's employment, use of public services (such as welfare and foster care), and involvement in the criminal justice system; and children's school records of their academic and behavioral functioning.

### Potential Risks

The potential risks to the participants involve the breach of confidentiality. To the extent that the data gathered are divulged to those who are not involved in the data gathering and processing, the subjects are at risk for a breach of their confidentiality. The potential for this problem is reduced by the use of identification codes rather than names on the data collection forms, keeping the data in locked file cabinets, and instituting procedures whereby breach of confidentiality by the data gathering and clerical staff is grounds for immediate termination. Individuals with daily access to the research data are the original data-gathering staff member, an on-site clerical staff member who reviews each completed interview to identify missing data or illogically coded responses, data entry staff based in Rochester, NY, and the Colorado and Rochester-based programming and analysis staff.

The participants also are at risk for psychological harm through the process of data gathering: the questions asked may increase their anxiety about their ability to care for their children and their living conditions. These risks are minimized by the interviewing staffs' reminding women at appropriate times that they do not have to answer particular questions or complete particular data-gathering procedures if they make them uncomfortable. We will avoid any reference to the risk status of the original sample. Some adolescents may feel uncomfortable with the explicit questions regarding sexuality. The interviewers will be trained to help participants with topics of sexuality and abuse should they arise. Women or children identified by interviewers at imminent risk because of suspected child abuse or neglect, spousal abuse, or suicidal ideation will be referred to a clinical psychologist on staff in Memphis who will make appropriate referrals to other community resources for assistance and will report to the proper authorities as required by law.

Urine screening for three STDs will also be conducted. This screening is done by assay and is non-invasive. A trained research assistant will instruct subjects in providing the urine specimen. Results will be obtained from the laboratory within 7 days and subjects will be informed. Those study subjects who test positive for an STD pathogen (chlamydia, gonorrhea or trichomonas) will be offered directly observed treatment, single session (DOT-SS) therapeutic regimen. We will treat infected study subjects with the following antimicrobials[218] each of which constitutes a single session therapeutic regimen (at no charge):

N. Gonorrhea - Cefixime (Suprax™)
C. Trachomatis - Azithromycin (Zithromax™)
T. Vaginalis – Metronidazole (Flagyl™)

An adolescent medicine physician at Planned Parenthood of Memphis will coordinate prescription of these oral medications. A nurse at Planned Parenthood will dispense the medication to infected study subjects. Symptomatic individuals will be treated at Planned Parenthood in accordance with their normal clinical protocols. As per state Department of Health guidelines, all Gonorrhea and Chlamydia infections will be reported to the Department of Health in Tennessee. CDC guidelines, Department of Health, and routine clinic procedures encourage infected individuals to inform their partners so that they can be tested and treated. The study team will follow these protocols per legal and ethical obligations. If we are unable to contact the study subject for STD

notification and/or to arrange treatment, we will notify the Department of Health, and they will initiate contact with the study subject and work with the infected individual to accomplish partner notification and contact tracing.

Another potential risk is that associated with adolescents learning that they have an STD. Adolescents may feel upset, uncomfortable or embarrassed when they are informed that they have an STD. We have extensive experience in addressing this sensitive issue. First, adolescents will be informed of their STD status in a confidential manner. Second, adolescents will be asked to go to Planned Parenthood for state-of-the-art treatment. In this study, to minimize invasive treatment procedures and poor adherence to treatment, only orally administered single-dose therapy will be utilized.

In Tennessee, minors may obtain STD treatment without consent of parents; accordingly parents will not be notified of STD results (or any other assessment results). Symptomatic individuals will be treated by Planned Parenthood's clinician in accordance with the site's normal clinical protocols. The clinician will also provide standard-of-care counseling routinely associated with STD treatment. While these procedures are designed to minimize adverse reactions, such reactions may still occur. A trained staff member will provide counseling for adolescents experiencing serious adverse psychological reactions as a result of the diagnosis or treatment for STD.

**Genotyping**

If participants consent, the interviewers will collect a spit sample using the Oragene procedure. DNA will be stored for analysis of specific genotypes that will be immediately tested (5HTTLPR, MAOA-LPR, and COMT val158met) as well as an "addiction panel." Genotypic data will be processed by the National Institute on Alcohol Abuse and Alcoholism. To protect participant confidentiality, all subjects receive code numbers that are stored separately from their personal identifiers. This record is kept in a locked file in the Memphis office and is not accessible to NIAAA personnel. Laboratory personnel enter all genotypic data into a database that contains only the subject's code number and that is accessible only by a password. The results will be stored on a limited-access computer, again labeled only with the code number. We are doing no forensic testing; since we are only testing three genes, the data generated will not and could not be used for patient identification. We will not be looking for paternity or genetic defects (like Huntington's disease, Tay-Sachs disease, or fragile X).

The DNA will be shared only according to the Resource Sharing Plan. Participants will be informed that the DNA may be tested at a later date for additional genes. The data to be collected in this study are experimental, and as such, there are no plans to share the data with subjects. Because we will not be sharing genetic results with participants or their physicians, and participants are not part of a clinical population at high risk for disorders, we do not see a significant risk for stigmatization or loss of insurance benefits if insurance carriers learn that study participants have participated in this study. We will not release genetic information to participants or their doctors even if asked to do so.

**Benefits**

There are no direct benefits to study participants, aside from the treatment of identified sexually transmitted infections in the participating youth, and counseling for adverse health behaviors. The problems addressed by this study are major public health problems. We hope that the intervention will be successful in reducing the rates of these problems in our subject population and think that the clear examination of these questions outweighs the previously mentioned risks. Even if a child and/or parent does not directly benefit from the project, the information we gain may be used to improve the effectiveness of STD education and intervention for other adolescents.

**Adequacy of Protection Against Risks**

**Recruitment Methods.** Letters will be sent to study participants announcing a new phase of the study. Study participants either will call the study office to indicate their desire to participate in the follow-up or the interviewing staff will call the study participants to determine their interest in continued participation. Study participants will be traced through telephone directories and lists of personal contacts given at earlier phases of the research to schedule an interview. If a study participant agrees to take part in the study, the study staff will schedule an appointment to conduct the interview in the study office. Or, if a study participant prefers to be interviewed at home, the study staff will schedule an appointment at a convenient time at the study participant's home.

If we learn that a study participant (either mother or child) is in prison, either from newspaper listings or through her personal contacts, the project coordinator will be responsible for both recruitment and consent

procedures. The project coordinator will call or write to the prison's warden to explain the nature of the current study, outline the study participant's previous involvement in this research, and ask for the warden's permission to request an interview with the study participant. If the warden gives his/her approval, he/she will be asked to communicate the interview request to the study participant. The project coordinator will not attempt to make contact with the study participant until the warden relates the study participant's express agreement.

**Consent Procedures.** During the interview, the interviewer will review the informed consents (one each for interview/assessments, physical health (including urinalysis), and genotyping) with the study participant, obtain consents for both her own and her child's participation, complete the interview, and explain the separate release forms. If the study participant chooses to sign the separate release forms, she will give us permission to abstract her social service records (TANF, Medicaid, Food Stamps, WIC, and child welfare services) and the study child's school records. Study children will sign assent forms agreeing to participate in the study.

If a study participant is in prison, the project coordinator will not attempt to make contact with the study participant until the warden relates the study participant's express agreement. During the interview, the project coordinator will review with the study participant informed consents specifically approved for use with incarcerated subjects. The project coordinator will remind the study participant that participation will not affect consideration for probation or parole in any way, that she is under no obligation to participate in the study, and that she has the right to refuse to answer questions or to terminate the interview at any time. If the study participant agrees to participate in the study, the project coordinator will proceed with the interview and explain the separate release forms. If the study participant chooses to sign the separate release forms, she will give us permission to abstract her social service records (TANF, Medicaid, Food Stamps, WIC, and child welfare services) and the study child's school records.

**Confidentiality of Data and Computer Records.** We will take the following steps to protect the confidentiality of our data and computer records:

- All personnel will sign an Assurance of Confidentiality form. Breach of confidentiality is grounds for immediate termination.
- Employees will not be allowed to abstract data, interview a subject, or process data from a subject whom they know personally.
- The data-collection manager will ensure that survey practices adhere to the provisions of the U.S. Privacy Act of 1974 with regard to surveys of individuals for the Federal government.
- Identification codes rather than names will be used on the data collection forms.
- In most cases, for confidentiality information, serial numbers are assigned to subjects prior to creating a machine-processible record and personal identifiers such as name, address, telephone number, and social security number are not part of the mechanism required for linkage of data files.
- The handling of data generally will be limited to the numerical values and statistical summaries.
- The identifiers linking identification codes with individual names are only available to the interviewers and PI and co-PIs.
- Data collection forms will be stored in a locked file drawer, preferably in a room that is locked when it is unoccupied.
- Data collection forms will be shredded when they are no longer relevant (e.g., after a paper is published).
- Attempts will be made to assign sensitive datasets filenames that do not obviously describe file contents.
- Personal computers will be logged off at the end of each work day to prevent inadvertent access to the network and to data stored on the computer hard drive.
- A screensaver password will be engaged during the day to prevent people from accessing data while individuals are out of the office.
- An access control list through arrangements with network administrators will be used to limit the number of people who have permission to access the network subdirectory where sensitive data is stored.
- Consideration may be given to storing sensitive data only on the network when it is being analyzed. More permanent storage would then be on a personal computer and/or computer disks.
- Data stored on computer disks will be limited to where it is absolutely necessary (e.g., where they are required for back up).

- Computer disks will be stored in a locked safe, preferably in a locked room to which only limited people have access.
- Sending sensitive data via email will be minimized.
- When emailing sensitive data the file will be zipped and assigned a password to protect it.

**Estimated Duration of Study**

The Age 17 follow-up will be conducted in Memphis, Tennessee. The interview and bio-assay portion of the study will take approximately 30 months to complete. Each assessment will take approximately three hours. Once the interview, testing, and assay data are gathered, we expect to have permission to review school and state records (TANF, Medicaid, Food Stamps, WIC, and child welfare services). The abstractions of records are expected to take 33 months to finish.

**Data And Safety Monitoring Plan**

**Monitoring:** We have created an independent Data Safety Monitoring Board consisting of Allison Kempe, M.D., Professor of Pediatrics at the University of Colorado, Zhaoxing Pan, biostatistician from The Children's Hospital Research Institute in Denver, and Catie Deines, a Certified Clinical Research Associate from The Children's Hospital Research Institute. This group will meet as needed to review any reports of untoward events and to monitor their resolution. At the stage of analysis and interpretation of data from the next phase of follow-up, this group will meet with the principal investigators and members of our biostatistical team to review the findings to determine whether there are any negative side effects. Minutes will be kept of the meeting and following DSMB meetings, the results of the meeting concerning possible negative side effects will be documented by Ms. Deines and sent to the PIs and appropriate NIH staff, or other appropriate institutions depending upon the nature of risk identified.

**Management of Untoward Events**

**How an "Untoward Effect" May Be Discovered:**

1. Data analysis uncovers treatment effects with higher rates of adverse events or outcomes in the nurse-visited groups compared to the control groups. While statistically significant differences may not pose a problem for individuals, the possibility of adverse program effects needs to be considered carefully.
2. Staff member discovers that participant personal health information has been revealed to individual or group who does not have permission for this information.
3. Participant verbalizes or displays high level anxiety or fear to the interviewer or other staff member regarding research procedures.

**How an Untoward Effect Will Be Reported:**

1. At the stage of data analysis, data analysts and investigators will keep a log of any test of treatment contrasts that produce statistically significant effects in the unexpected direction, or a pattern of trends in the unexpected direction. The Data Safety Monitoring Board group will meet with the principal investigators and members of our biostatistical team to review the findings to determine whether there are any negative side effects. Following the DSMB meeting, the results of the meeting concerning possible negative side effects will be documented by Ms. Catie Deines (a member of our Data-Safety Monitoring Board) and sent to the PIs, the IRB, and appropriate NIH staff.
2. Any breach of confidentiality is cause for termination. Breaches of confidentiality will be identified and reported to the DSMB and IRB by the PI whenever they occur. The DSMB will report these infractions to the IRB and NIH.
3. Instances of extreme anxiety or fear will be noted in a confidential log and reported to the PI, who in turn will report this information to the DSMB. A record of these problems will be submitted to the IRB.

**Referral of Cases for Further Evaluation and Treatment:** Should an Untoward Event occur, the data gathering staff in Memphis will discuss the untoward event with their supervisor, who will make an appropriate referral for further evaluation and treatment within the health and human service delivery system in Memphis. The Memphis leadership staff members have been with the study for nearly 15 years and are well-positioned to evaluate and make appropriate community referrals, given the types of untoward effects that may occur in this study.

**Follow-up and Referral:** The PI will be informed of all reported adverse outcomes, including who is referred for further evaluation and treatment and the outcomes.

**Confidentiality:** The PI will maintain information on monitoring untoward events, but will do so without specific patient identifiers.

**Implementation of Monitoring Recommendations:** The monitoring board will review cases and make written recommendations to the PI and investigative team regarding safety procedures and the procedures employed in the study to ensure participant safety. These recommendations will be reviewed at special meetings of investigators and research team members to institute any changes in procedures that would improve participant safety and that would ensure that they receive appropriate treatment and follow-up in a timely way.

**Special Consent Issues**

**Prisoners.** We have information that a small number of women who had participated in earlier phases of this longitudinal study are currently in prison. Our decision to recruit these women for the Age 17 follow-up is rooted in our concern for the study's scientific integrity; namely, excluding this segment of the sample would create a potential bias in our trial, given that a central aspect of the study is to ascertain the extent to which the intervention prevents criminal behavior, including arrests, convictions, and incarceration. The same applies to youth involved in the study, in that one of the major outcomes of the study is to ascertain its impact on the rates of criminal offending in the mothers' offspring. To exclude these participants form the follow-up would create a significant impediment to its scientific integrity.

We understand that care must be taken in enrolling prisoners in any research and that special care must be given to vulnerable populations, such as those who are incarcerated. The recruitment and consent procedures we have outlined earlier are designed to protect all incarcerated subjects in our study from being coerced to participate in any way. In addition, we plan to obtain a Certificate of Confidentiality as we have in previous follow-ups of this study to protect our data from forced disclosure.

**Potential Benefits of the Proposed Research to the Subjects and Others**

There are no direct tangible benefits to the individual participants in this study, except that youth will be screened for sexually transmitted infections and given free one-dose treatment for those infections identified. Moreover, all youth will be provided counseling to address those health risks identified during the health examination. Aside from the potential breach of confidentiality, the risks of participating in this research are minimal, however, and the benefits to society are substantial.

**Importance of the Knowledge To Be Gained**

An earlier trial of this program conducted with a primarily white sample showed substantial impact on women's use of welfare, criminal behavior, substance abuse, and child abuse and neglect. The children whose mothers participated in the program showed reductions in running away, emergent use of substances, and arrests and convictions by the children's $15^{th}$ birthday. If these findings are replicated with minorities living in a major urban area, we will have identified a preventive intervention that has the potential to reduce major social and health problems facing low-income parents and children. Given the minimal risks and substantial knowledge to be gained, this study has a high benefit to risk ratio.

**Collaborating Sites**

This study will be conducted in collaboration with the University of Colorado Health Sciences Center (federal-wide assurance number FWA00005070). Other collaborating sites include Emory University (FWA00005792), the National Institute on Alcohol Abuse and Alcoholism (FWA00005897), University of Colorado at Boulder (FWA00003492), Yale University (FWA00002571), and Research Triangle Institute (FWA00003331). In addition, we have consultants from Pennsylvania State University (FWA00001534) and Cornell University (FWA00004513).

**Inclusion of Women**

The proposed study will follow 670 women and their children who participated in earlier phases of this work. The focus of this study is to test the impact of the program on the adaptive functioning and health of low-income women bearing first children and on the health and development of their children. Approximately half of the women's children are male and half are female.

**Inclusion of Minorities**

Ninety two percent of the sample is African-American and 8% are Caucasian. Given that the original test of this intervention was conducted with a primarily white sample, it was necessary to determine whether the benefits observed for whites could be reproduced for African-Americans. This racial composition of the sample is thus necessary given the research questions addressed.

## Targeted/Planned Enrollment Table

**This report format should NOT be used for data collection from study participants.**

**Study Title:** Age-17 Follow-up of Home Visiting Intervention

**Total Planned Enrollment:** 2043

| TARGETED/PLANNED ENROLLMENT: Number of Subjects | | | |
|---|---|---|---|
| **Ethnic Category** | **Sex/Gender** | | |
| | **Females** | **Males** | **Total** |
| Hispanic or Latino | 10 | 5 | 15 |
| Not Hispanic or Latino | 1347 | 681 | 2028 |
| **Ethnic Category: Total of All Subjects *** | 1357 | 686 | 2043 |
| **Racial Categories** | | | |
| American Indian/Alaska Native | 0 | 0 | 0 |
| Asian | 0 | 0 | 0 |
| Native Hawaiian or Other Pacific Islander | 0 | 0 | 0 |
| Black or African-American | 1248 | 631 | 1879 |
| White | 109 | 55 | 164 |
| **Racial Categories: Total of All Subjects *** | 1357 | 686 | 2043 |

* The “Ethnic Category: Total of All Subjects” must be equal to the “Racial Categories: Total of All Subjects.”

**Inclusion of Children**

A major focus of this research is on the prevention of developmental problems in children. We will assess 670 firstborn children.

**F. Vertebrate Animals**

This does not apply to this study.

**G. Select Agent Research**

This does not apply to this study.

**I. Multiple PIs Leadership Plan**

This does not apply to this study.

**J. Consortium/Contractual Arrangements**

**University of Colorado Health Sciences Center (UCHSC)**

We have developed a consortium agreement with the University of Colorado Health Sciences Center for three primary reasons: 1) David Olds, Ph.D., the Principal Investigator on this study currently holds his primary institutional affiliation with the Department of Pediatrics there; 2) Dennis Luckey, Ph.D., Director of Data Operations at the Prevention Research Center for Family and Child Health (in Pediatrics at UCHSC), is responsible for overseeing (with Chuck Henderson) and conducting analyses with the UCHSC co-investigators; and 3) Christian Hopfer, M.D., associate professor of psychiatry, a member of the team examining substance abuse. Dr. Olds was a faculty member at the University of Rochester (and currently has an adjunct appointment) when the Memphis study was first funded. The current proposal has been submitted through the University of Rochester (UR) because most of the staff involved in the conduct of the current phase of this work are UR employees and because this arrangement minimizes indirect costs.

**Emory University**

We have developed a consortium agreement with Emory University for the involvement of Drs. Gina Wingood, and Ralph DiClemente, and Angela Caliendo to serve as co-investigators on this study. Dr. Wingood, who will serve as the Emory PI on this grant, is an internationally recognized expert in the prevention of HIV, and the reduction of risky sexual behavior. Dr. DiClemente is an international leader in AIDS prevention research, with particular expertise in the prevention of AIDS among African-American youth. Dr. Angela Caliendo is the Core Co-Director and their staff are experts in HIV prevention, risky sexual behavior, STD and substance testing through urinalysis. The Emory team will assume responsibility for the analysis of data on STDs, risky sexual behavior, and the reduction of HIV risk, in collaboration with Dr. Luckey from the University of Colorado Prevention Research Center for Family and Child Health.

The Emory Center for AIDS Research is one of 20 Centers for Aids Research across the United States funded by the National Institutes of Health. The Emory Center's scientific leadership and administrative team help to facilitate the domestic and international research of over 100 Emory CFAR investigators, associate investigators, and adjunct investigators who work in one or more of five HIV/AIDS research domains. Units of the CFAR are administratively located in Emory College, the Rollins School of Public Health, the Emory University School of Medicine, and the Emory Vaccine Center.

**Research Triangle Institute (RTI) International**

We will develop a consortium agreement with RTI for the involvement of Dr. Diana Fishbein to serve as a co-investigator on this grant. Dr. Fishbein, director of the Transdisciplinary Behavioral Science Program (TBSP) at RTI, will oversee the neuropsychological testing of the children and the analysis of adolescents' neuropsychological data in collaboration with Drs. Dennis Luckey, John Holmberg, and David Olds at the Prevention Research Center for Family and Child Health at the University of Colorado.

Dr. Fishbein created the TBSP at RTI International in 1999 to integrate individual-level factors (e.g., biologic and psychologic) with contextual factors measured at the environmental, social, and cultural levels to advance an understanding of high risk behaviors and to identify underlying mechanisms in differential responses to interventions. TBSP links a broad range of capabilities across disciplines within the behavioral sciences; integrates diverse areas of expertise to enhance studies on prevention and treatment of high risk behaviors and associated personality characteristics; applies animal and clinical models of substance abuse to treatment and prevention protocols; estimates relative contributions of genetic and social factors in human behavior; examines effects of physical and social environmental inputs on biological systems and gene expressions; identifies measurable changes in brain function resulting from effective intervention programs; identifies protective factors that suppress or buffer the effects of vulnerability factors; predicts individual responses to treatment interventions using integrated data sets; determines the extent to which vulnerability factors are malleable; and applies innovative and cutting edge technologies to the science, treatment, and prevention of risk behaviors.

**University of Colorado at Boulder**

We will develop a consortium agreement with the University of Colorado at Boulder for the involvement of 2 co-investigators: Dr. Terence Thornberry, Director of the Problem Behavior Program at the Institute for Behavioral Science, and Dr. Robin Corley, Senior Research Associate at the Institute for Behavioral Genetics.

***Institute for Behavioral Science.*** Dr. Thornberry, an internationally recognized sociologist, who specializes in adolescent problem behavior will oversee the analysis of youth problem behavior (delinquency, youth involvement in the criminal justice system). The statistical analysis of data will be conducted by Dr.

Dennis Luckey and the data operations team at the Prevention Research Center for Family and Child Health in the Department of Pediatrics at the University of Colorado at Denver and Health Sciences Center.
The Program on Problem Behavior, which Dr. Thornberry directs at the Institute for Behavioral Science at CU Boulder, is an interdisciplinary research effort seeking to understand the nature, course of development, and later life consequences of behaviors that put young people's health and development at risk. Research in the Program has focused on delinquency, illicit drug use, alcohol abuse, cigarette smoking, precocious sexual activity, school failure and drop out, violence and aggression, risky driving, running away, health-compromising behaviors such as insufficient exercise and unhealthy diet, evaluation and dissemination of violence, drug, and delinquency programs, and mental health problems such as depression. The Program on Problem Behavior continues to be recognized as one of the strongest interdisciplinary research efforts on problem behavior in the United States and internationally.

***Institute for Behavioral Genetics.*** Dr. Corley join the team as a co-investigator, focusing on the development of adolescent substance use and disorders. He will join Dr. Hopfer and Dr. Luckey to analyze the data on substance use and abuse. He also will serve as a member of the team led by Dr. Enoch at NIAAA to examine gene x treatment x environment interactions on maternal and adolescent substance use and disorder. Dr. Corley has been a Principal Investigator since 1992 of one component of a multi-component center studying adolescent antisocial substance abuse, and has been involved in both linkage and large-scale (>1000 SNP) association studies of the co-morbid problem behaviors of substance dependence and antisocial personality. The mission of the Institute for Behavioral Genetics, an organized research unit of the University of Colorado at Boulder, is to conduct and facilitate research on the genetic bases of individual differences in behavior. The detection, location, and identification of individual quantitative trait loci, using both linkage and association methods, is a high priority at IBG.

**K. Resource Sharing**

In reviewing this data-sharing plan, it is important to note that we are not drawing blood and creating cell lines for this study, as we believe that doing so in this highly vulnerable population (very poor African-American adolescents and their mothers) will undermine participation in the study, or at very least lead to unacceptable levels of missing data for DNA extraction. We are working with the Laboratory of NeuroGenetics (LNG) at NIAA, which will conduct the genotyping. We do wish to participate in NIDA’s Genetics Consortium, however, and to have the NIDA Center for Genetics Studies manage the data sharing. The following outlines our data-sharing plan:

1. Saliva samples from each subject will be processed with Oragene and sent to LNG at NIAAA. At the NIAAA lab, genomic DNA will be extracted and diluted to 100 ng/µl. Genotyping for 5-HT will be done by methods described in Hu et al.,[144] and MAO-A by methods describe in Ducci et al.[231] Genotyping of the COMT polymorphism will be conducted in conjunction with genotyping for 186 ancestry-informative markers (AIMS) as well as additional candidate genes for substance dependence, depression, and stress-response using a large-scale approach for identifying vulnerability genes in addiction and psychiatric disorders. An Illumina platform will be used to assay a total of 1,350 Tag SNPs, which cover 130 genes (including COMT) and which also generates a set of 186 ancestry informative markers (AIMS) which can be used to control for population stratification.
2. The NIAAA team will complete the genotyping and analysis of gene x environment interactions within 18 months after completion of the primary grant that funds this work. In order to conduct this work, the entire longitudinal data set for this trial, consisting of, among other data domains, participant treatment assignment, baseline maternal and family background characteristics, prenatal health conditions and behaviors, family contextual factors measured from pregnancy through the first child's 17th birthday, histories of child injuries and health conditions, qualities of parental care-giving, and data on participant phenotypes from infancy through age 17, will be made available by Principal Investigator, David Olds, Ph.D. to the NIAAA team. The following dataset, including genetics data, will be made available to NCGS within 18 months after the NIDA grant expires.
3. The data provided for each subject will include:
    a. Subject ID #
    b. Family ID #
    c. Mother ID #
    d. Sex
    e. Death Status

f. Ethnicity of geographic origin of ancestry
g. Age at time of most recent assessment
h. Date of most recent assessment
i. Twin Status
j. DSM-IV diagnoses
k. CIDI diagnoses
l. Instruments used to establish diagnoses
m. Answers to all of the questions in the structured interview.
n. Other descriptive information collected about the drug abuse/addiction phenotypes, such as age of onset, quantity and frequency of peak lifetime use of addictive substance, etc.
o. Genotyping data, including the DNA marker names, description of SNPs, or allele sizes in base pairs and corresponding frequencies, and relative map distances.

4. Data will be verified and provided to the NCGS within 18 months after termination of the grant.
5. Verified genotyping data will be submitted to the NCGS at the time that data will be made available for sharing.
6. The approved informed consents for this study are consistent with this sharing plan. Annual updates and modifications will be sent as approved by the IRB.
7. David Olds, Ph.D., the principal investigator, has read and agrees to the terms set forth in the FAQ documents for the Genetics Work Group.
8. We will make the data available from this trial available for distribution 18 months after termination of the grant period, including extension, or immediately upon first publication of the data, whichever comes first. It is our understanding that the NCGS will distribute data to qualified investigators who have been approved by the NIDA Genetic Data Access Request Committee who have signed the Distribution Agreement.
9. Dr. Olds will request membership in the NIDA Genetics Consortium
10. Dr. Olds will participate in NGC meetings, which are held 2 times per year. A budget supplement will be requested to cover these travel costs.

In addition to sharing data through the NIDA Center for Genetics Studies, we will make non-genomic data from this phase of the trial available to qualified investigators through the University of Michigan's Inter-university Consortium for Political Science and Social Research (ICPSR). We have established a repository for the data from earlier phases of the Memphis trial with ICPSR and will do so for this phase of the study. Once data from the current phase of follow-up have been thoroughly cleaned, documented and the analytical results published for this phase of data gathering, we shall deposit those data, a file contents listing, and codebook with ICPSR.

**L. Consultants**

**Linda Collins, Ph.D.** Professor Collins is Director of the Methodology Center at Penn State, where she has focused her career on the development and evaluation of cutting edge statistical methods for behavioral and biomedical applications. She has developed a method known as Latent Transition Analysis, a method for fitting a wide range of stage-sequential models in longitudinal research. Given our reinvigorated focus on the longitudinal aspects of this study, we expect to rely on Professor Collins for assistance in the longitudinal analyses.

**Charles Henderson.** Henderson is statistician and Senior Research Associate at Cornell University. He has played a central role in the design and analysis of the trials of the Nurse Family Partnership since the first trial was initiated in Elmira, New York in 1977. Mr. Henderson will continue to consult on this phase of the Memphis trial, giving particular attention to mixed models, random regressions, semi-paramentric methods, mediation analyses and developing methods and programs for analysis of complex models as may be required.

We are including letters from these two consultants.

## Reference List


1. Findings from the aspirin component of the ongoing Physicians' Health Study. N Engl J Med 1988 Jan 28;318(4):262-4.

2. Trikalinos TA, Salanti G, Khoury MJ, Ioannidis JP. Impact of violations and deviations in Hardy-Weinberg equilibrium on postulated gene-disease associations. Am J Epidemiol 2006 Feb 15;163(4):300-9.

3. Pritchard JK, Stephens M, Rosenberg NA, Donnelly P. Association mapping in structured populations. Am J Hum Genet 2000 Jul;67(1):170-81.

4. Hopfer CJ, Lessem JM, Hartman CA, Stallings MC, Cherny SS, Corley RP, Hewitt JK, Krauter KS, Mikulich-Gilbertson SK, Rhee SH, et al. A genome-wide scan for loci influencing adolescent cannabis dependence symptoms: evidence for linkage on chromosomes 3 and 9. Drug Alcohol Depend 2007 Jun 15;89(1):34-41.

5. Olds DL, Sadler L, Kitzman H. Programs for parents of infants and toddlers: recent evidence from randomized trials. Journal of Child Psychology and Psychiatry 2007;48(3/4):355-91. **PMID: 17355402. Available free at *Journal of Child Psychology and Psychiatry.***

6. Olds DL, Eckenrode J, Henderson CR Jr, Kitzman H, Powers J, Cole R, Sidora K, Morris P, Pettitt LM, Luckey D. Long-term effects of home visitation on maternal life course and child abuse and neglect. Fifteen-year follow-up of a randomized trial. JAMA 1997 Aug 27;278(8):637-43. **PubMedID: 9272895. Not available for free**.

7. Fishbein DH, Hyde C, Eldreth D, Paschall MJ, Hubal R, Das A, Tarter R, Ialongo N, Hubbard S, Yung B. Neurocognitive skills moderate urban male adolescents' responses to preventive intervention materials. Drug Alcohol Depend 2006 Mar 15;82(1):47-60.

8. Kendall PC, Kessler RC. The impact of childhood psychopathology interventions on subsequent substance abuse: policy implications, comments, and recommendations. J Consult Clin Psychol 2002 Dec;70(6):1303-6.

9. Olds DL. Prenatal and infancy home visiting by nurses: from randomized trials to community replication. Prevention Science 2002 Sep;3(3):153-72. **PubMedID: 12387552. Not available for free.**

10. Olds DL, Hill PL, O'Brien R, Racine D, Moritz P. Taking preventive intervention to scale: the Nurse-Family Partnership. Cognitive and Behavioral Practice 2003;10(4):278-90. **Not available for free.**

11. Olds D, Henderson CR Jr, Cole R, Eckenrode J, Kitzman H, Luckey D, Pettitt L, Sidora K, Morris P, Powers J. Long-term effects of nurse home visitation on children's criminal and antisocial behavior: 15-year follow-up of a randomized controlled trial . JAMA 1998 Oct 14;280(14):1238-44. **PubMedID: 9786373. Available free at *JAMA*.**

12. Kellam, S. G.; Werthamer-Larsson, L. Developmental epidemiology: a basis for prevention. In: Kessler, M.; Goldston, S. E., eds. A decade of progress in primary prevention. Hanover, NH: University Press of New England; 1986. pp. 154-80.

13. Olds DL, Kitzman H, Cole R, Robinson J. Theoretical foundations of a program of home visitation for pregnant women and parents of young children. J Community Psychol

1997;25(1):9-25. **Not available for free.**

14. Elster AB, McAnarney ER. Medical and psychosocial risks of pregnancy and childbearing during adolescence. Pediatr Ann 1980 Mar;9(3):89-94.

15. Overpeck MD, Brenner RA, Trumble AC, Trifiletti LB, Berendes HW. Risk factors for infant homicide in the United States. N Engl J Med 1998 Oct 22;339(17):1211-6.

16. Olds DL. The Nurse-Family Partnership: an evidence-based preventive intervention. Infant Mental Health Journal 2006;27(1):5-25. **Available free at *Infant Mental Health Journal*.**

17. Wang X, Zuckerman B, Pearson C, Kaufman G, Chen C, Wang G, Niu T, Wise PH, Bauchner H, Xu X. Maternal cigarette smoking, metabolic gene polymorphism, and infant birth weight. JAMA 2002 Jan 9;287(2):195-202.

18. Kahn RS, Khoury J, Nichols WC, Lanphear BP. Role of dopamine transporter genotype and maternal prenatal smoking in childhood hyperactive-impulsive, inattentive, and oppositional behaviors. J Pediatr 2003 Jul;143(1):104-10.

19. Meaney MJ, Szyf M, Seckl JR. Epigenetic mechanisms of perinatal programming of hypothalamic-pituitary-adrenal function and health. Trends Mol Med 2007 Jun 1.

20. Barker DJ, Gluckman PD, Godfrey KM, Harding JE, Owens JA, Robinson JS. Fetal nutrition and cardiovascular disease in adult life. Lancet 1993 Apr 10;341(8850):938-41.

21. Gluckman PD, Hanson MA. The developmental origins of the metabolic syndrome. Trends Endocrinol Metab 2004 May-2004 Jun 30;15(4):183-7.

22. Kaffman A, Meaney MJ. Neurodevelopmental sequelae of postnatal maternal care in rodents: clinical and research implications of molecular insights. J Child Psychol Psychiatry 2007 Mar-2007 Apr 30;48(3-4):224-44 .

23. Kramer MS. Intrauterine growth and gestational duration determinants. Pediatrics 1987 Oct;80(4):502-11.

24. Fried PA, Watkinson B, Dillon RF, Dulberg CS. Neonatal neurological status in a low-risk population after prenatal exposure to cigarettes, marijuana, and alcohol. J Dev Behav Pediatr 1987 Dec;8(6):318-26.

25. Sood B, Delaney-Black V, Covington C, Nordstrom-Klee B, Ager J, Templin T, Janisse J, Martier S, Sokol RJ. Prenatal alcohol exposure and childhood behavior at age 6 to 7 years: I. dose-response effect. Pediatrics 2001 Aug;108(2):E34.

26. Mayes LC. Neurobiology of prenatal cocaine exposure: effect on developing monoamine systems. Infant Mental Health Journal 1994;15:121-33.

27. Milberger S, Biederman J, Faraone SV, Chen L, Jones J. Is maternal smoking during pregnancy a risk factor for attention deficit hyperactivity disorder in children? Am J Psychiatry 1996 Sep;153(9):1138-42.

28. Olds DL, Henderson CR Jr, Tatelbaum R. Intellectual impairment in children of women who smoke cigarettes during pregnancy. Pediatrics 1994 Feb;93(2):221-7. **PubMedID:**

**8121734. Available free at *Pediatrics*.**

29. Olds DL, Henderson CR Jr, Tatelbaum R. Prevention of intellectual impairment in children of women who smoke cigarettes during pregnancy. Pediatrics 1994 Feb;93(2):228-33. **PubMedID: 7510063. Available free at *Pediatrics*.**

30. Olds DL. Tobacco exposure and impaired development: a review of the evidence. Mental Retardation and Developmental Disabilities Research Reviews 1997;3:257-69. **Not available for free.**

31. Streissguth, A. P.; Sampson, P. D.; Barr, H. M., et al. The effects of prenatal exposure to alcohol and tobacco: contributions from the Seattle longitudinal prospective study and implications for public policy. In: Needleman, H. L.; Bellinger, D., eds. Prenatal exposure to toxicants: developmental consequences. Baltimore: Johns Hopkins University Press; 1994. pp. 148-83.

32. Clark AS, Soto S, Bergholz TSM. Maternal gestational stress alters adaptive and social behavior in adolescent rhesus monkey offspring. Infant Behav Dev 1996;19:453-63.

33. Saxon DW. The behavior of infants whose mothers smoke in pregnancy. Early Hum Dev 1978;2:363-9.

34. Brookes KJ, Mill J, Guindalini C, Curran S, Xu X, Knight J, Chen CK, Huang YS, Sethna V, Taylor E, et al. A common haplotype of the dopamine transporter gene associated with attention-deficit/hyperactivity disorder and interacting with maternal use of alcohol during pregnancy. Arch Gen Psychiatry 2006 Jan; 63(1):74-81.

35. Arseneault L, Tremblay RE, Boulerice B, Saucier JF. Obstetrical complications and violent delinquency: testing two developmental pathways. Child Dev 2002 Mar-2002 Apr 30;73(2):496-508.

36. Cole R, Henderson CRJ, Kitzman H, Anson E, Eckenrode J, Sidora K. Long-term effects of nurse home visitation on maternal employment. Unpublished Manuscript 2004.

37. Peterson, L.; Gable, S. Holistic injury prevention. In: Lutzker, John R., ed. Handbook of child abuse research and treatment. New York: Plenum Press; 1998. pp. 291-318.

38. Hart, B.; Risley, T. R. Meaningful differences in the everyday experience of young American children. Baltimore, MD: Paul Brookes; 1995.

39. Pine DS. Affective neuroscience and the development of social anxiety disorder. Psychiatr Clin North Am 2001 Dec;24(4):689-705.

40. Pine DS. Developmental psychobiology and response to threats: relevance to trauma in children and adolescents. Biol Psychiatry 2003 May 1;53(9):796-808.

41. Bremner JD, Vermetten E. Neuroanatomical changes associated with pharmacotherapy in posttraumatic stress disorder. Ann N Y Acad Sci 2004 Dec;1032:154-7.

42. Harvey E, Danforth JS, Ulaszek WR, Eberhardt TL. Validity of the parenting scale for parents of children with attention-deficit/hyperactivity disorder. Behav Res Ther 2001 Jun;39(6):731-43.

43. Davidson RJ, Putnam KM, Larson CL. Dysfunction in the neural circuitry of emotion regulation--a possible prelude to violence. Science 2000 Jul 28;289(5479):591-4.

44. Dodge KA, Bates JE, Pettit GS. Mechanisms in the cycle of violence. Science 1990 Dec 21;250(4988):1678-83.

45. Bremner JD. Does stress damage the brain? Biol Psychiatry 1999 Apr 1;45(7):797-805.

46. Field TM, Scafidi F, Pickens J, Prodromidis M, Pelaez-Nogueras M, Torquati J, Wilcox H, Malphurs J, Schanberg S, Kuhn C. Polydrug-using adolescent mothers and their infants receiving early intervention. Adolescence 1998 Spring;33(129):117-43.

47. Biglan A, Duncan TE, Ary DV, Smolkowski K. Peer and parental influences on adolescent tobacco use. J Behav Med 1995 Aug;18 (4):315-30.

48. Baumrind, D. Familial antecedents of adolescent drug use: a developmental perspective. National Institute of Drug Abuse Monograph 56 (DHHS Publication No. ADM 87-1335). Washington, DC: US Government Printing Office; 1987.

49. Cohen E, Navaline H, Metzger D. High-risk behaviors for HIV: a comparison between crack-abusing and opioid-abusing African-American women. J Psychoactive Drugs 1994 Jul-1994 Sep 30;26(3):233-41.

50. Grant KE, O'Koon JH, Davis TH, Roache NA, Poindexter LM, Armstrong ML, Minden JA, McIntosh JM. Protective factors affecting low-income urban African American youth exposed to stress. Journal of Early Adolescence 2000;20:388-417.

51. Johnson V, Pandina RJ. Familial and personal drinking histories and measures of competence in youth. Addict Behav 1991;16(6):453-65.

52. Furstenberg, F. F.; Brooks-Gunn, J.; Morgan, S. P. Adolescent mothers in later life. Cambridge: Cambridge University Press; 1987.

53. McLanahan SS, Carlson MJ. Welfare reform, fertility, and father involvement. Future Child 2002;12(1):146-65.

54. Kim-Cohen J, Caspi A, Taylor A, Williams B, Newcombe R, Craig IW, Moffitt TE. MAOA, maltreatment, and gene-environment interaction predicting children's mental health: new evidence and a meta-analysis. Mol Psychiatry 2006 Oct;11(10):903-13.

55. Jaffee SR, Moffitt TE, Caspi A, Fombonne E, Poulton R, Martin J. Differences in early childhood risk factors for juvenile-onset and adult-onset depression. Arch Gen Psychiatry 2002 Mar;59(3):215-22.

56. Duncan GJ, Brooks-Gunn J, Klebanov PK. Economic deprivation and early childhood development. Child Dev 1994 Apr;65(2 Spec No):296-318.

57. Duncan GJ, Brooks-Gunn J. Family poverty, welfare reform, and child development. Child Dev 2000 Jan-2000 Feb 28;71(1):188-96.

58. Brooks-Gunn, J. ; Duncan, G. J.; Aber, J. L. Neighborhood poverty: context and consequences for children. Vol. I. New York: Russell Sage; 1997.

59. Hawkins JD, Catalano RF, Miller JY. Risk and protective factors for alcohol and other drug problems in adolescence and early adulthood: implications for substance abuse prevention. Psychol Bull 1992 Jul;112(1):64-105.

60. Greene, J. P. High school graduation rates in the United States: revised. New York: Manhattan Institute for Policy Research; 2001.

61. Clark DB, Cornelius J. Childhood psychopathology and adolescent cigarette smoking: a prospective survival analysis in children at high risk for substance use disorders. Addict Behav 2004 Jun;29(4):837-41.

62. Wakschlag LS, Pickett KE, Cook E Jr, Benowitz NL, Leventhal BL. Maternal smoking during pregnancy and severe antisocial behavior in offspring: a review. Am J Public Health 2002 Jun;92(6):966-74.

63. Moffitt TE. Adolescence-limited and life-course-persistent antisocial behavior: a developmental taxonomy. Psychol Rev 1993 Oct;100(4):674-701.

64. Raine A, Brennan P, Mednick SA. Birth complications combined with early maternal rejection at age 1 year predispose to violent crime at age 18 years. Arch Gen Psychiatry 1994 Dec;51(12):984-8.

65. McCauley J, Kern DE, Kolodner K, Dill L, Schroeder AF, DeChant HK, Ryden J, Derogatis LR, Bass EB. Clinical characteristics of women with a history of childhood abuse: unhealed wounds. JAMA 1997 May 7;277 (17):1362-8.

66. Allers CT, Benjack KJ. Connection between childhood abuse and HIV infection . J Couns Dev 1991;70:309-13.

67. Gunnar M, Quevedo K. The neurobiology of stress and development. Annu Rev Psychol 2007;58:145-73.

68. Rutter M, Moffitt TE, Caspi A. Gene-environment interplay and psychopathology: multiple varieties but real effects. J Child Psychol Psychiatry 2006 Mar-2006 Apr 30;47(3-4):226-61.

69. Tarter RE, Kirisci L, Mezzich A, Cornelius JR, Pajer K, Vanyukov M, Gardner W, Blackson T, Clark D. Neurobehavioral disinhibition in childhood predicts early age at onset of substance use disorder. Am J Psychiatry 2003 Jun;160(6):1078-85.

70. Clark DB, Cornelius JR, Kirisci L, Tarter RE. Childhood risk categories for adolescent substance involvement: a general liability typology. Drug Alcohol Depend 2005 Jan 7;77(1):13-21.

71. Loeber R. The stability of antisocial and delinquent child behavior: a review. Child Dev 1982 Dec;53(6):1431-46.

72. Wakschlag LS, Leventhal BL, Pine DS, Pickett KE, Carter AS. Elucidating early mechanisms of developmental psychopathology: the case of prenatal smoking and disruptive behavior. Child Dev 2006;77(4):893-906.

73. Aytaclar S, Tarter RE, Kirisci L, Lu S. Association between hyperactivity and executive cognitive functioning in childhood and substance use in early adolescence. J Am Acad

Child Adolesc Psychiatry 1999 Feb;38(2):172-8.

74. Konradi C, Heckers S. Molecular aspects of glutamate dysregulation: implications for schizophrenia and its treatment. Pharmacol Ther 2003 Feb;97(2):153-79.

75. Boyle MH, Offord DR, Racine YA, Szatmari P, Fleming JE, Links PS. Predicting substance use in late adolescence: results from the Ontario Child Health Study follow-up. Am J Psychiatry 1992 Jun;149(6):761-7.

76. Clark DB, Pollock N, Bukstein OG, Mezzich AC, Bromberger JT, Donovan JE. Gender and comorbid psychopathology in adolescents with alcohol dependence. J Am Acad Child Adolesc Psychiatry 1997 Sep;36(9):1195-203.

77. Lynskey MT, Heath AC, Bucholz KK, Slutske WS, Madden PA, Nelson EC, Statham DJ, Martin NG. Escalation of drug use in early-onset cannabis users vs co-twin controls. JAMA 2003 Jan 22-2003 Jan 29;289(4):427-33.

78. Ridenour TA, Cottler LB, Robins LN, Compton WM, Spitznagel EL, Cunningham-Williams RM. Test of the plausibility of adolescent substance use playing a causal role in developing adulthood antisocial behavior. J Abnorm Psychol 2002 Feb;111(1):144-55.

79. Clark DB, Parker AM, Lynch KG. Psychopathology and substance-related problems during early adolescence: a survival analysis. J Clin Child Psychol 1999 Sep;28(3):333-41.

80. Giancola PR, Zeichner A. Alcohol-related aggression in males and females: effects of blood alcohol concentration, subjective intoxication, personality, and provocation. Alcohol Clin Exp Res 1995 Feb;19(1):130-4.

81. Giancola PR, Zeichner A, Yarnell JE, Dickson KE. Relation between executive cognitive functioning and the adverse consequences of alcohol use in social drinkers. Alcohol Clin Exp Res 1996 Sep;20(6):1094-8.

82. Grant I, Adams KM, Carlin AS, Rennick PM, Judd LL, Schooff K. The Collaborative Neuropsychological Study of Polydrug Users. Arch Gen Psychiatry 1978;35:1063-74.

83. Meek PS, Clark HW, Solana VL. Neurocognitive impairment: the unrecognized component of dual diagnosis in substance abuse treatment. J Psychoactive Drugs 1989 Apr-1989 Jun 30;21(2):153-60.

84. Brutus M, Shaikh MB, Edinger H, Siegel A. Effects of experimental temporal lobe seizures upon hypothalamically elicited aggressive behavior in the cat. Brain Res 1986 Feb 26;366(1-2):53-63.

85. Elliott FA. Violence. The neurologic contribution: an overview. Arch Neurol 1992 Jun ;49(6):595-603.

86. Volavka, J. Neurobiology of violence. Washington, DC: American Psychiatric Press; 1995.

87. Fishbein DH, Eldreth DL, Hyde C, Matochik JA, London ED, Contoreggi C, Kurian V, Kimes AS, Breeden A, Grant S. Risky decision making and the anterior cingulate cortex in abstinent drug abusers and nonusers. Brain Res Cogn Brain Res 2005 Apr;23(1):119-36.

88. Fried PA, Smith AM. A literature review of the consequences of prenatal marihuana exposure. An emerging theme of a deficiency in aspects of executive function. Neurotoxicol Teratol 2001 Jan-2001 Feb 28;23(1):1-11.

89. Bell MA, Fox NA. The relations between frontal brain electrical activity and cognitive development during infancy. Child Dev 1992 Oct;63(5):1142-63.

90. Levin HS, Culhane KA, Hartmann J, Evankovich J, Mattson AJ, Harward H, Ringholz G, Ewing-Cobbs L, Fletcher JM. Developmental changes in performance on tests of purported frontal lobe functioning. Developmental Neuropsychology 1991;7:377-95.

91. Thatcher RW. Maturation of the frontal lobes: physiological evidence for aging. Developmental Neuropsychology 1991;7:397-419.

92. Thatcher RW. Cyclic cortical reorganization during early childhood. Brain Cogn 1992 Sep;20(1):24-50.

93. Welsh MC, Pennington BF, Groisser DB. A normative-developmental study of executive function: a window on prefrontal function in children. Developmental Neuropsychology 1991;7:131-49.

94. Bremner JD, Narayan M, Staib LH, Southwick SM, McGlashan T, Charney DS. Neural correlates of memories of childhood sexual abuse in women with and without posttraumatic stress disorder. Am J Psychiatry 1999 Nov;156(11):1787-95.

95. Bremner, J. D.; Southwick, S. M.; Charney, D. S. The neurobiology of posttraumatic stress disorder: an integration of animal and human research. In: Saigh, P.; Bremner, J. D., eds. Posttraumatic stress disorder: a comprehensive text. New York: Allyn & Bacon; 1999.

96. Bremner JD, Vermetten E, Mazure CM. Development and preliminary psychometric properties of an instrument for the measurement of childhood trauma: the Early Trauma Inventory. Depress Anxiety 2000;12(1):1-12.

97. Critchley HD, Elliot R, Mathias CJ, Dolan RJ. Neural activity relating to generation and representation of galvanic skin conductance responses: a functional magnetic resonance imaging study. J Neurosci 2000;20:3033-40.

98. Fishbein DH. Neuropsychological dysfunction, drug abuse, and violence: conceptual framework, and preliminary findings. Criminal Justice and Behavior 2000;27:139-59.

99. Steckler T, Holsboer F. Corticotropin-releasing hormone receptor subtypes and emotion. Biol Psychiatry 1999 Dec 1;46(11):1480-508.

100. Davidson R. Asymmetric brain function, affective style, and psychopathology: the role of early experience and plasticity. Dev Psychopathol 1994;58:741-58.

101. de Haan, M.; Luciana, M.; Malone, S., et al. Development, plasticity, and risk: commentary on Huttenlocher, Pollitt and Gorman, and Gottesman and Goldsmith. In: Nelson, C. A., ed. Threats to optimal development: integrating biological, psychological, and social risk factors. Hillsdale, NJ: Lawrence Erlbaum; 1994. pp. 161-78.

102. Tarter RE, Blackson T, Brigham J, Moss H, Caprara GV. The association between childhood

irritability and liability to substance use in early adolescence: a 2-year follow-up study of boys at risk for substance abuse. Drug Alcohol Depend 1995 Oct;39(3):253-61.

103. Division of Exceptional Children and Health Services The 2003 Youth Risk Behavior Survey: Memphis City Schools outcomes. Memphis: Memphis City Schools Office of Research and Evaluation; 2004.

104. Dependence, abuse, and treatment [homepage on the Internet]. SAMHSA Office of Applied Studies; c2006 [cited 2007 Jun 21]. Available from: http://www.oas.samhsa.gov/NSDUH/2k5NSDUH/tabs/Sect5peTabs1to82.htm#Tab5.3B.

105. Leckman JF, Herman AE. Maternal behavior and developmental psychopathology. Biol Psychiatry 2002 Jan 1;51(1):27-43.

106. Denenberg VH, Rosenberg KM, Paschke R, Zarrow MX. Mice reared with rat aunts: effects on plasma corticosterone and open field activity. Nature 1969 Jan 4;221(5175):73-4.

107. Francis D, Diorio J, Liu D, Meaney MJ. Nongenomic transmission across generations of maternal behavior and stress responses in the rat. Science 1999 Nov 5;286(5442):1155-8.

108. Levine, S. Psychosocial factors in growth and development. In: Levi, L., ed. Society, Stress, and Disease. London: Oxford University Press; 1975. pp. 43-50.

109. Blume AW, Davis JM, Schmaling KB. Neurocognitive dysfunction in dually-diagnosed patients: a potential roadblock to motivating behavior change. J Psychoactive Drugs 1999 Apr-1999 Jun 30;31(2):111-5.

110. Ruppenthal GC, Arling GL, Harlow HF, Sackett GP, Suomi SJ. A 10-year perspective of motherless-mother monkey behavior. J Abnorm Psychol 1976 Aug;85(4):341-9.

111. Caspi A, Sugden K, Moffitt TE, Taylor A, Craig IW, Harrington H, McClay J, Mill J, Martin J, Braithwaite A, et al. Influence of life stress on depression: moderation by a polymorphism in the 5-HTT gene. Science 2003 Jul 18;301(5631):386-9.

112. Malhotra AK, Kestler LJ, Mazzanti C, Bates JA, Goldberg T, Goldman D. A functional polymorphism in the COMT gene and performance on a test of prefrontal cognition. Am J Psychiatry 2002 Apr;159(4):652-4.

113. Caspi A, Moffitt TE, Cannon M, McClay J, Murray R, Harrington H, Taylor A, Arseneault L, Williams B, Braithwaite A, et al. Moderation of the effect of adolescent-onset cannabis use on adult psychosis by a functional polymorphism in the catechol-O-methyltransferase gene: longitudinal evidence of a gene X environment interaction. Biol Psychiatry 2005 May 15;57 (10):1117-27.

114. Harlow, H. F.; Harlow, C. M. From learning to love. New York: Praeger Pub Text; 1987.

115. Giancola PR, Mezzich AC, Tarter RE. Disruptive, delinquent and aggressive behavior in female adolescents with a psychoactive substance use disorder: relation to executive cognitive functioning. J Stud Alcohol 1998 Sep;59(5):560-7.

116. Moss HB, Talagala SL, Kirisci L . Phosphorus-31 magnetic resonance brain spectroscopy of children at risk for a substance use disorder: preliminary results. Psychiatry Res 1997

Dec 30;76(2-3):101-12.

117. Weinberg NZ. Cognitive and behavioral deficits associated with parental alcohol use. J Am Acad Child Adolesc Psychiatry 1997 Sep;36(9):1177-86.

118. Kessler RC, Sonnega A, Bromet E, Hughes M, Nelson CB. Posttraumatic stress disorder in the National Comorbidity Survey. Arch Gen Psychiatry 1995 Dec;52(12):1048-60.

119. Kendler KS, Bulik CM, Silberg J, Hettema JM, Myers J, Prescott CA. Childhood sexual abuse and adult psychiatric and substance use disorders in women: an epidemiological and cotwin control analysis. Arch Gen Psychiatry 2000 Oct;57(10):953-9.

120. Widom CS. The cycle of violence. Science 1989 Apr 14;244(4901):160-6.

121. Keiley MK, Howe TR, Dodge KA, Bates JE, Petti GS. The timing of child physical maltreatment: a cross-domain growth analysis of impact on adolescent externalizing and internalizing problems. Dev Psychopathol 2001 Fall;13(4):891-912.

122. Wilhelm K, Mitchell PB, Niven H, Finch A, Wedgwood L, Scimone A, Blair IP, Parker G, Schofield PR. Life events, first depression onset and the serotonin transporter gene. Br J Psychiatry 2006 Mar;188:210-5.

123. Taylor SE, Way BM, Welch WT, Hilmert CJ, Lehman BJ, Eisenberger NI. Early family environment, current adversity, the serotonin transporter promoter polymorphism, and depressive symptomatology. Biol Psychiatry 2006 Oct 1;60(7):671-6.

124. Eley TC, Sugden K, Corsico A, Gregory AM, Sham P, McGuffin P, Plomin R, Craig IW. Gene-environment interaction analysis of serotonin system markers with adolescent depression. Mol Psychiatry 2004 Oct;9(10):908-15.

125. Barr CS, Newman TK, Becker ML, Champoux M, Lesch KP, Suomi SJ, Goldman D, Higley JD. Serotonin transporter gene variation is associated with alcohol sensitivity in rhesus macaques exposed to early-life stress. Alcohol Clin Exp Res 2003 May;27(5):812-7.

126. Suomi SJ. Risk, resilience, and gene x environment interactions in rhesus monkeys . Annals of the New York Academy of Science 2006;1094:52-62.

127. Chipman P, Jorm AF, Prior M, Sanson A, Smart D, Tan X, Easteal S. No interaction between the serotonin transporter polymorphism (5-HTTLPR) and childhood adversity or recent stressful life events on symptoms of depression: Results from two community surveys. Am J Med Genet B Neuropsychiatr Genet 2007 Jun 5;144(4):561-5.

128. Chorbov VM, Lobos EA, Todorov AA, Heath AC, Botteron KN, Todd RD. Relationship of 5-HTTLPR genotypes and depression risk in the presence of trauma in a female twin sample . Am J Med Genet Part B 2007 Apr 23;epub.

129. Kaufman J, Yang BZ, Douglas-Palumberi H, Houshyar S, Lipschitz D, Krystal JH, Gelernter J. Social supports and serotonin transporter gene moderate depression in maltreated children. Proc Natl Acad Sci U S A 2004 Dec 7;101(49):17316-21.

130. Huizinga D, Haberstick BC, Smolen A, Menard S, Young SE, Corley RP, Stallings MC, Grotpeter J, Hewitt JK. Childhood maltreatment, subsequent antisocial behavior, and the role of monoamine oxidase a genotype. Biol Psychiatry 2006 Oct 1;60(7):677-83.

131. Young SE, Smolen A, Hewitt JK, Haberstick BC, Stallings MC, Corley RP, Crowley TJ. Interaction between MAO-A genotype and maltreatment in the risk for conduct disorder: failure to confirm in adolescent patients. Am J Psychiatry 2006 Jun;163(6):1019-25.

132. Caspi A, McClay J, Moffitt TE, Mill J, Martin J, Craig IW, Taylor A, Poulton R. Role of genotype in the cycle of violence in maltreated children. Science 2002 Aug 2;297(5582):851-4.

133. Nilsson KW, Sjoberg RL, Wargelius HL, Leppert J, Lindstrom L, Oreland L. The monoamine oxidase A (MAO-A) gene, family function and maltreatment as predictors of destructive behaviour during male adolescent alcohol consumption. Addiction 2007 Mar;102(3):389-98.

134. Rutter M. Gene-environment interdependence. Dev Sci 2007 Jan;10(1):12-8.

135. Jabbi M, Korf J, Kema IP, Hartman C, van der Pompe G, Minderaa RB, Ormel J, den Boer JA. Convergent genetic modulation of the endocrine stress response involves polymorphic variations of 5-HTT, COMT and MAOA. Mol Psychiatry 2007 May;12(5):483-90.

136. Jabbi M, Kema IP, van der Pompe G, te Meerman GJ, Ormel J, den Boer JA. Catechol-o-methyltransferase polymorphism and susceptibility to major depressive disorder modulates psychological stress response. Psychiatr Genet 2007 Jun;17(3):183-93.

137. Ducci F, Enoch MA, Hodgkinson C, Xu K, Catena M, Robin RW, Goldman D. Interaction between a functional MAOA locus and childhood sexual abuse predicts alcoholism and antisocial personality disorder in adult women . Mol Psychiatry in press.

138. Sen S, Burmeister M, Ghosh D. Meta-analysis of the association between a serotonin transporter promoter polymorphism (5-HTTLPR) and anxiety-related personality traits. Am J Med Genet B Neuropsychiatr Genet 2004 May 15;127(1):85-9.

139. Feinn R, Nellissery M, Kranzler HR. Meta-analysis of the association of a functional serotonin transporter promoter polymorphism with alcohol dependence. Am J Med Genet B Neuropsychiatr Genet 2005 Feb 5;133(1):79-84.

140. Hariri AR, Drabant EM, Munoz KE, Kolachana BS, Mattay VS, Egan MF, Weinberger DR. A susceptibility gene for affective disorders and the response of the human amygdala. Arch Gen Psychiatry 2005 Feb;62(2):146-52.

141. Heinz A, Braus DF, Smolka MN, Wrase J, Puls I, Hermann D, Klein S, Grusser SM, Flor H, Schumann G, et al. Amygdala-prefrontal coupling depends on a genetic variation of the serotonin transporter. Nat Neurosci 2005 Jan;8(1):20-1.

142. Pezawas L, Meyer-Lindenberg A, Drabant EM, Verchinski BA, Munoz KE, Kolachana BS, Egan MF, Mattay VS, Hariri AR, Weinberger DR. 5-HTTLPR polymorphism impacts human cingulate-amygdala interactions: a genetic susceptibility mechanism for depression. Nat Neurosci 2005 Jun;8(6):828-34.

143. Urry HL, van Reekum CM, Johnstone T, Kalin NH, Thurow ME, Schaefer HS, Jackson CA, Frye CJ, Greischar LL, Alexander AL, et al. Amygdala and ventromedial prefrontal cortex are inversely coupled during regulation of negative affect and predict the diurnal pattern of cortisol secretion among older adults. J Neurosci 2006 Apr 19;26(16):4415-25.

144. Hu XZ, Lipsky RH, Zhu G, Akhtar LA, Taubman J, Greenberg BD, Xu K, Arnold PD, Richter MA, Kennedy JL, et al. Serotonin transporter promoter gain-of-function genotypes are linked to obsessive-compulsive disorder. Am J Hum Genet 2006 May;78(5):815-26.

145. Sabol SZ, Hu S, Hamer D. A functional polymorphism in the monoamine oxidase A gene promoter. Hum Genet 1998 Sep;103(3):273-9.

146. Shih JC, Chen K, Ridd MJ. Monoamine oxidase: from genes to behavior. Annu Rev Neurosci 1999;22:197-217.

147. Sjoberg RL, Ducci F, Barr CS, Newman TK, Dell'osso L, Virkkunen M, Goldman D. A Non-Additive Interaction of a Functional MAO-A VNTR and Testosterone Predicts Antisocial Behavior. Neuropsychopharmacology 2007 Apr 11.

148. Carrel L, Willard HF. X-inactivation profile reveals extensive variability in X-linked gene expression in females. Nature 2005 Mar 17;434(7031):400-4.

149. Huang YY, Cate SP, Battistuzzi C, Oquendo MA, Brent D, Mann JJ. An association between a functional polymorphism in the monoamine oxidase a gene promoter, impulsive traits and early abuse experiences. Neuropsychopharmacology 2004 Aug;29(8):1498-505.

150. Weinshilboum RM, Otterness DM, Szumlanski CL. Methylation pharmacogenetics: catechol O-methyltransferase, thiopurine methyltransferase, and histamine N-methyltransferase. Annu Rev Pharmacol Toxicol 1999;39 :19-52.

151. de Frias CM, Annerbrink K, Westberg L, Eriksson E, Adolfsson R, Nilsson LG. Catechol O-methyltransferase Val158Met polymorphism is associated with cognitive performance in nondemented adults. J Cogn Neurosci 2005 Jul;17(7):1018-25.

152. Rosa A, Peralta V, Cuesta MJ, Zarzuela A, Serrano F, Martinez-Larrea A, Fananas L. New evidence of association between COMT gene and prefrontal neurocognitive function in healthy individuals from sibling pairs discordant for psychosis. Am J Psychiatry 2004 Jun;161(6):1110-2.

153. Diamond A, Briand L, Fossella J, Gehlbach L. Genetic and neurochemical modulation of prefrontal cognitive functions in children. Am J Psychiatry 2004 Jan;161(1):125-32.

154. Horowitz R, Kotler M, Shufman E, Aharoni S, Kremer I, Cohen H, Ebstein RP. Confirmation of an excess of the high enzyme activity COMT val allele in heroin addicts in a family-based haplotype relative risk study. Am J Med Genet 2000 Oct 9;96(5):599-603.

155. Li T, Chen CK, Hu X, Ball D, Lin SK, Chen W, Sham PC, Loh el-W, Murray RM, Collier DA. Association analysis of the DRD4 and COMT genes in methamphetamine abuse. Am J Med Genet B Neuropsychiatr Genet 2004 Aug 15;129(1):120-4.

156. Vandenbergh DJ, Rodriguez LA, Miller IT, Uhl GR, Lachman HM. High-activity catechol-O-methyltransferase allele is more prevalent in polysubstance abusers. Am J Med Genet 1997 Jul 25;74(4):439-42.

157. Thapar A, Langley K, Fowler T, Rice F, Turic D, Whittinger N, Aggleton J, Van den Bree M, Owen M, O'Donovan M. Catechol O-methyltransferase gene variant and birth weight predict early-onset antisocial behavior in children with attention-deficit/hyperactivity disorder. Arch Gen Psychiatry 2005 Nov;62(11):1275-8.

158. Enoch MA, Xu K, Ferro E, Harris CR, Goldman D. Genetic origins of anxiety in women: a role for a functional catechol-O-methyltransferase polymorphism. Psychiatr Genet 2003 Mar;13(1):33-41.

159. Olsson CA, Anney RJ, Lotfi-Miri M, Byrnes GB, Williamson R, Patton GC. Association between the COMT Val158Met polymorphism and propensity to anxiety in an Australian population-based longitudinal study of adolescent health. Psychiatr Genet 2005 Jun;15(2):109-15.

160. Zubieta JK, Heitzeg MM, Smith YR, Bueller JA, Xu K, Xu Y, Koeppe RA, Stohler CS, Goldman D. COMT val158met genotype affects mu-opioid neurotransmitter responses to a pain stressor. Science 2003 Feb 21;299(5610):1240-3.

161. Tarter RE, Kirisci L, Habeych M, Reynolds M, Vanyukov M. Neurobehavior disinhibition in childhood predisposes boys to substance use disorder by young adulthood: direct and mediated etiologic pathways. Drug Alcohol Depend 2004 Feb 7;73(2):121-32.

162. Desrichard O, Denarie V. Sensation seeking and negative affectivity as predictors of risky behaviors: a distinction between occasional versus frequent risk-taking. Addict Behav 2005 Aug;30(7):1449-53.

163. Cook RL, Clark DB. Is there an association between alcohol consumption and sexually transmitted diseases? A systematic review. Sex Transm Dis 2005 Mar;32(3):156-64.

164. Cook RL, Pollock NK, Rao AK, Clark DB. Increased prevalence of herpes simplex virus type 2 among adolescent women with alcohol use disorders. J Adolesc Health 2002;30(3):169-74.

165. Wasserheit JN. Effect of changes in human ecology and behavior on patterns of sexually transmitted diseases, including human immunodeficiency virus infection. Proc Natl Acad Sci U S A 1994 Mar 29;91(7):2430-5.

166. Eng, T.; Butler, W. The hidden epidemic: confronting sexually transmitted diseases. Washington, DC: National Academy Press; 1997.

167. Greenblatt RM, Bacchetti P, Barkan S, Augenbraun M, Silver S, Delapenha R, Garcia P, Mathur U, Miotti P, Burns D. Lower genital tract infections among HIV-infected and high-risk uninfected women: findings of the Women's Interagency HIV Study (WIHS). Sex Transm Dis 1999 Mar;26(3):143-51.

168. Parazzini F, D'Oro LC, Naldi L, Bianchi C, Chatenoud L, Ricci E, Cainelli T, Pansera B, Mezzanotte C, Tessari GP, et al. Sexually transmitted diseases and risk of HIV infection. Acta Derm Venereol 1996 Mar;76(2):147-9.

169. Rotchford K, Strum AW, Wilkinson D. Effect of coinfection with STDs and of STD treatment on HIV shedding in genital-tract secretions: systematic review and data synthesis. Sex Transm Dis 2000 May;27(5):243-8.

170. Crosby R, DiClemente RJ, Wingood GM, Harrington K, Davies SL, Hook EW 3rd, Oh MK. Predictors of infection with Trichomonas vaginalis: a prospective study of low income African-American adolescent females. Sex Transm Infect 2002 Oct;78(5):360-4.

171. Karoly, L. A.; Greenwood, P. W.; Everingham, S. S.; Hoube, J.; Kilburn, M. R.; Rydell, C. P.;

Sanders, M.; Chiesa, J. Investing in our children: what we know and don't know about the costs and benefits of early childhood interventions. Santa Monica, CA: RAND; 1998.

172. Olds DL, Henderson CR Jr, Phelps C, Kitzman H, Hanks C. Effect of prenatal and infancy nurse home visitation on government spending. Med Care 1993 Feb;31(2):155-74. **PubMedID: 8433578. Not available for free.**

173. Lauritsen JL. The social ecology of violent victimization: individual and contextual effects in the NCVS. Journal of Quantitative Criminology 2001;17(1):3-32.

174. Theodore AD, Chang JJ, Runyan DK, Hunter WM, Bangdiwala SI, Agans R. Epidemiologic features of the physical and sexual maltreatment of children in the Carolinas. Pediatrics 2005 Mar;115(3):e331-7.

175. Olds D, Henderson CR Jr, Kitzman H, Cole R. Effects of prenatal and infancy nurse home visitation on surveillance of child maltreatment . Pediatrics 1995 Mar;95(3):365-72. **PubMedID: 7862474. Available free at *Pediatrics*.**

176. Buchsbaum HK, Toth SL, Clyman RB, Cicchetti D, Emde RN. The use of a narrative story stem technique with maltreated children: implications for theory and practice. Dev Psychopathol 1992;4(603-625).

177. Olds DL, Kitzman H, Cole R, Robinson J, Sidora K, Luckey D, Henderson C, Hanks C, Bondy J, Holmberg J. Effects of nurse home visiting on maternal life-course and child development: age-six follow-up of a randomized trial. Pediatrics 2004;114:1550-9. **PubMedID: 15574614. Available free at *Pediatrics*.**

178. Gooding DC, Matts CW, Rollmann EA. Sustained attention deficits in relation to psychometrically identified schizotypy: evaluating a potential endophenotypic marker. Schizophr Res 2006 Feb 15;82(1):27-37.

179. Raine A, Moffitt TE, Caspi A, Loeber R, Stouthamer-Loeber M, Lynam D. Neurocognitive impairments in boys on the life-course persistent antisocial path. J Abnorm Psychol 2005 Feb;114(1):38-49.

180. Kitzman H, Olds DL, Sidora K, Henderson CR Jr, Hanks C, Cole R, Luckey DW, Bondy J, Cole K, Glazner J. Enduring effects of nurse home visitation on maternal life course: a 3-year follow-up of a randomized trial. JAMA 2000 Apr 19;283(15):1983-9. **PubMedID: 10789666. Available free at *JAMA*.**

181. Kitzman H, Olds DL, Henderson CR Jr, Hanks C, Cole R, Tatelbaum R, McConnochie KM, Sidora K, Luckey DW, Shaver D, et al. Effect of prenatal and infancy home visitation by nurses on pregnancy outcomes, childhood injuries, and repeated childbearing. A randomized controlled trial. JAMA 1997 Aug 27;278(8):644-52. **PubMedID: 9272896. Not available for free**.

182. Soares JF, Wu CF. Some restricted randomization rules in sequential designs. Common Stat Theory Methods 1983;12:2017-34.

183. Caspi A, Moffitt TE. Gene-environment interactions in psychiatry: joining forces with neuroscience. Nat Rev Neurosci 2006 Jul;7(7):583-90.

184. Gottesman II, Gould TD. The endophenotype concept in psychiatry: etymology and strategic

intentions. Am J Psychiatry 2003 Apr;160(4):636-45.

185. Cottler LB, Robins LN, Helzer JE. The reliability of the CIDI-SAM: a comprehensive substance abuse interview. Br J Addict 1989 Jul;84(7):801-14.

186. Robins, L.; Marcus, L.; Reich, W.; Cunningham, R.; Gallagher, T. The Diagnostic Interview Schedule Version IV. St. Louis, MO: Washington University; 1996.

187. Kessler RC, McGonagle KA, Zhao S, Nelson CB, Hughes M, Eshleman S, Wittchen HU, Kendler KS. Lifetime and 12-month prevalence of DSM-III-R psychiatric disorders in the United States. Results from the National Comorbidity Survey. Arch Gen Psychiatry 1994 Jan;51(1):8-19.

188. Crowley TJ, Mikulich SK, Ehlers KM, Whitmore EA, MacDonald MJ. Validity of structured clinical evaluations in adolescents with conduct and substance problems. J Am Acad Child Adolesc Psychiatry 2001 Mar;40(3):265-73.

189. Tarter R, Laird S, Bukstein O, Kaminer Y. Validation of the Drug Use Screening Inventory: preliminary findings. Psychology of Addictive Behaviors 1992;6:233-6.

190. Stanley SM, Markman HJ, Whitton SW. Communication, conflict, and commitment: insights on the foundations of relationship success from a national survey. Fam Process 2002 Winter;41(4):659-75.

191. Straus MA. Measuring intrafamily conflict and violence: the Conflict Tactics (CT) Scales. J Marriage and the Family 1979;41:75-88.

192. Steer, R. A.; Beck, A. T. Beck Depression Inventory (BDI). In: Sederer, L. I.; Dickey, B., eds. Outcome assessment in clinical practice. Baltimore, MD: Williams & Wilkins; 1996.

193. Wilson, K.; de Beurs, E.; Palmer, C., et al. Beck Anxiety Inventory. In: Maruish, M. E., ed. The use of psychological testing for treatment planning and outcomes assessment. Mahwah, NJ: Lawrence Erlbaum; 1999. pp. 971-93.

194. Jacob T, Moser RP, Windle M, Loeber R, Stouthamer-Loeber M. A new measure of parenting practices involving preadolescent- and adolescent-aged children. Behav Modif 2000 Oct;24(5):611-34.

195. Slate JR, Graham LS, Bower J. Relationships of the WISC-R and K-BIT for an adolescent clinical sample. Adolescence 1996 Winter;31(124):777-82.

196. Rogers RD, Owen AM, Middleton HC, Williams EJ, Pickard JD, Sahakian BJ, Robbins TW. Choosing between small, likely rewards and large, unlikely rewards activates inferior and orbital prefrontal cortex. J Neurosci 1999 Oct 15;19(20):9029-38.

197. Bechara A, Damasio H, Damasio AR. Emotion, decision making and the orbitofrontal cortex. Cereb Cortex 2000 Mar;10(3):295-307.

198. Bolla KI, Eldreth DA, London ED, Kiehl KA, Mouratidis M, Contoreggi C, Matochik JA, Kurian V, Cadet JL, Kimes AS, et al. Orbitofrontal cortex dysfunction in abstinent cocaine abusers performing a decision-making task. Neuroimage 2003 Jul;19(3):1085-94.

199. Horn NR, Dolan M, Elliott R, Deakin JF, Woodruff PW. Response inhibition and impulsivity: an

fMRI study. Neuropsychologia 2003;41(14):1959-66.

200. Conners, C. K. Conners' continuous performance test computer program, user's manual. North Tonawanda, NY: Multi-Health Systems; 1995.

201. Ekman, P.; Friesen, W. V. Pictures of facial affect. Palo Alto, CA: Consulting Psychologists Press; 1975.

202. Blair RJ, Cipolotti L. Impaired social response reversal. A case of 'acquired sociopathy'. Brain 2000 Jun;123 ( Pt 6):1122-41.

203. Blair RJ, Colledge E, Murray L, Mitchell DG. A selective impairment in the processing of sad and fearful expressions in children with psychopathic tendencies. J Abnorm Child Psychol 2001 Dec;29(6):491-8.

204. Cadesky EB, Mota VL, Schachar RJ. Beyond words: how do children with ADHD and/or conduct problems process nonverbal information about affect? J Am Acad Child Adolesc Psychiatry 2000 Sep;39(9):1160-7.

205. Kornreich C, Foisy ML, Philippot P, Dan B, Tecco J, Noel X, Hess U, Pelc I, Verbanck P. Impaired emotional facial expression recognition in alcoholics, opiate dependence subjects, methadone maintained subjects and mixed alcohol-opiate antecedents subjects compared with normal controls. Psychiatry Res 2003 Aug 1;119(3):251-60.

206. Kossen DS, Suchy Y, Mayer AR, Libby J. Facial affect recognition in criminal psychopaths. Emotion 2002;2(4):398-411.

207. Achenbach, T. M.; Rescorla, L. A. Manual for ASEBA school-age forms & profiles. Burlington, VT: University of Vermont, Research Center for Children, Youth, & Families; 2001.

208. Kirisci L, Mezzich A, Tarter R. Norms and sensitivity of the adolescent version of the drug use screening inventory. Addict Behav 1995 Mar-1995 Apr 30;20(2):149-57.

209. Tarter R, Mezzich A, Kirisci L, Kaczynski N. Reliability of the Drug Use Screening Inventory among adolescent alcoholics. Journal of Child and Adolescent Substance Abuse 1994;3(1):25-36.

210. Anton RF, Lieber C, Tabakoff B. Carbohydrate-deficient transferrin and gamma-glutamyltransferase for the detection and monitoring of alcohol use: results from a multisite study. Alcohol Clin Exp Res 2002 Aug;26(8):1215-22.

211. Kauth MR, St Lawrence JS, Kelly JA. Reliability of retrospective assessments of sexual HIV risk behavior: a comparison of biweekly, three-month, and twelve-month self-reports. AIDS Educ Prev 1991 Fall;3(3):207-14.

212. St Lawrence JS. African-American adolescents' knowledge, health-related attitudes, sexual behavior, and contraceptive decisions: implications for the prevention of adolescent HIV infection. J Consult Clin Psychol 1993 Feb;61(1):104-12.

213. Leigh BC, Morrison DM, Trocki K, Temple MT. Sexual behavior of American adolescents: results from a U.S. national survey. J Adolesc Health 1994 Mar;15(2):117-25.

214. St Lawrence JS, Brasfield TL, Jefferson KW, Alleyne E, O'Bannon RE 3rd, Shirley A.

Cognitive-behavioral intervention to reduce African American adolescents' risk for HIV infection. J Consult Clin Psychol 1995 Apr;63(2):221-37.

215. Carroll KC, Aldeen WE, Morrison M, Anderson R, Lee D, Mottice S. Evaluation of the Abbott LCx ligase chain reaction assay for detection of Chlamydia trachomatis and Neisseria gonorrhoeae in urine and genital swab specimens from a sexually transmitted disease clinic population. J Clin Microbiol 1998 Jun;36(6):1630-3.

216. Gaydos CA, Howell MR, Quinn TC, Gaydos JC, McKee KT Jr. Use of ligase chain reaction with urine versus cervical culture for detection of Chlamydia trachomatis in an asymptomatic military population of pregnant and nonpregnant females attending Papanicolaou smear clinics. J Clin Microbiol 1998 May;36(5):1300-4.

217. Madico G, Quinn TC, Rompalo A, McKee KT Jr, Gaydos CA. Diagnosis of Trichomonas vaginalis infection by PCR using vaginal swab samples. J Clin Microbiol 1998 Nov;36(11):3205-10.

218. Workowski KA, Berman SM. Sexually transmitted diseases treatment guidelines, 2006. MMWR Recomm Rep 2006 Aug 4;55(RR-11):1-94.

219. Conard, L. A.; Fortenberry, J. D.; Vanderpol, B. Trichomonas vaginalis in adolescent and young adult males: detection by polymerase chain reaction. San Diego, CA: Society for Adolescent Medicine; 2001.

220. Hook EW 3rd. Trichomonas vaginalis--no longer a minor STD. Sex Transm Dis 1999 Aug;26(7):388-9.

221. van Der Schee C, van Belkum A, Zwijgers L, van Der Brugge E, O'neill EL, Luijendijk A, van Rijsoort-Vos T, van Der Meijden WI, Verbrugh H, Sluiters HJ. Improved diagnosis of Trichomonas vaginalis infection by PCR using vaginal swabs and urine specimens compared to diagnosis by wet mount microscopy, culture, and fluorescent staining. J Clin Microbiol 1999 Dec;37(12):4127-30.

222. Sturdevant MS, Kohler CL, Williams LF, Johnson JE. The University of Alabama Teenage Access Project: a model for prevention, referrals, and linkages to testing for high-risk young women. J Adolesc Health 1998 Aug;23(2 Suppl):107-14.

223. Fortenberry JD , Brizendine EJ, Katz BP, Wools KK, Blythe MJ, Orr DP. Subsequent sexually transmitted infections among adolescent women with genital infection due to Chlamydia trachomatis, Neisseria gonorrhoeae, or Trichomonas vaginalis. Sex Transm Dis 1999 Jan;26(1):26-32.

224. Elliott, D. S. ; Huizinga, D.; Menard, S. Multiple problem youth: delinquency, substance use, and mental health problems. New York: Springer-Verlag; 1989.

225. Geene, J. P. High school graduation rates in the United States: revised. New York: Manhattan Institute for Policy Research; 2001.

226. Hastings TL, Kelley ML. Development and validation of the Screen for Adolescent Violence Exposure (SAVE). J Abnorm Child Psychol 1997 Dec;25(6):511-20.

227. Sampson RJ, Raudenbush SW, Earls F. Neighborhoods and violent crime: a multilevel study of collective efficacy. Science 1997 Aug 15;277(5328):918-24.

228. Lauritsen, J. L. How families and communities influence youth victimization. Washington, DC: Office of Justice Programs, U.S. Department of Justice; 2003.

229. Sampson RJ, Morenoff JD, Gannon-Rowley T. Assessing "neighborhood effects": social processes and new directions in research. Annual Review of Sociology 2002;28:443-78.

230. Rylander-Rudqvist T, Hakansson N, Tybring G, Wolk A. Quality and quantity of saliva DNA obtained from the self-administrated oragene method--a pilot study on the cohort of Swedish men. Cancer Epidemiol Biomarkers Prev 2006 Sep;15(9):1742-5.

231. Ducci F, Newman TK, Funt S, Brown GL, Virkkunen M, Goldman D. A functional polymorphism in the MAOA gene promoter (MAOA-LPR) predicts central dopamine function and body mass index. Mol Psychiatry 2006 Sep;11 (9):858-66.

232. Clogg CC, Goodman LA. Latent structure analysis of a set of multidimensional contingency tables. Journal of the American Statistical Association 1984;79:762-71 .

233. Collins LM, Graham JW, Rousculp SS, Fidler PL, Pan J, Hansen WB. Latent transition analysis and how it can address prevention research questions. NIDA Res Monogr 1994;142:81-111.

234. Searle, S. R. Linear models. New York: Wiley; 1971.

235. Henderson CR, Henderson CRJr. Analysis of covariance in mixed models with unequal subclass numbers. Communications in Statistics, Series A 1979;8:751-87.

236. Henderson CR Jr. Analysis of covariance in the mixed model: higher-level, nonhomogeneous, and random regressions. Biometrics 1982 Sep;38(3):623-40.

237. Henderson, C. R. Applications of linear models in animal breeding. Guelph: University of Guelph; 1984.

238. Laird NM, Ware JH. Random-effects models for longitudinal data. Biometrics 1982 Dec;38(4):963-74.

239. Searle, S. R.; Casella, G.; McCulloch, C. E. Variance components. New York: Wiley; 1992.

240. Hastie, T.; Tibshirani, R. Generalized additive models. New York: Chapman and Hall; 1990.

241. Ruppert, D.; Wand, M. P.; Carroll, R. J. Semiparametric regression. New York: Cambridge University Press; 2003.

242. McCullagh, P.; Nelder, J. A. Generalized linear models. London: Chapman and Hall; 1983.

243. Baker, R. I.; Nelder, J. A. The GLIM system--release three: generalized linear interactive modeling. Oxford: Numerical Algorithms Group; 1978.

244. Schall R. Estimation in generalized linear models with random effects. Biometrika 1991;78:719-27.

245. Wolfinger R, O'Connell M. Generalized linear mixed models: a pseudo-likelihood approach. Journal of Statistical and Computational Simulation 1993;48:233-43.

246. Bollen, K. A. Structural equations with latent variables. New York: Wiley; 1989.

247. Schmidt, P. Econometrics. New York: Marcel Dekker; 1976.

248. Goldberger, A. Econometric methods. New York: John Wiley & Sons; 1964.

249. Henderson, C.; Ceci, S. Is it better to be born rich or smart: a bioecological analysis of the contributions of IQ and socioeconomic status to adult income. In: Billingsley, K. R.; Brown, H. U.; Derohanes, E., eds. Scientific Excellence in Supercomputing: The IBM 1990 Contest Prize Papers. Athens, GA: The Baldwin Press, University of Georgia; 1992.

250. Little, R. J. A.; Rubin, D. B. Statistical analysis with missing data, 2nd ed. New York: John Wiley and Sons; 2002.

251. MacKinnon DP, Lockwood CM, Hoffman JM, West SG, Sheets V. A comparison of methods to test mediation and other intervening variable effects. Psychol Methods 2002 Mar;7(1):83-104.

252. Olds DL, Luckey DW, Henderson CRJ. Can the results be believed?: In Reply. Pediatrics 2005;115:1113-4. **Available free at *Pediatrics*.**
