# Supplementary material for: Early Home Visits and Health Outcomes in Low-Income Mothers and Offspring: 18-Year Follow-Up of a Randomized Clinical Trial
Source: JAMA Netw Open. 2024 Jan 18;7(1):e2351752. doi: 10.1001/jamanetworkopen.2023.51752 (PMC10797459; doi:10.1001/jamanetworkopen.2023.51752)
Supplement: Supplement 2. — eMethods. eTable 1. CONSORT Information for Mothers and Children Enrolled in the Trial Through the 18-Year Follow-Up eTable 2. Balancing Tests, Baseline Covariate eTable 3. Ex-Post Power Calculations, Main Outcome eTable 4. NFP Treatment Effects on Sample Retention eTable 5. NFP Effects on Length of Gestation, Child Birthweight, Weight at Age 2 Years eTable 6. NFP Effects on Maternal Blood Pressure at Birth, Pregnancy-Induced Hypertension, and Health Conditions at Child Age 18 Years eTable 7. Mediation Analysis, Mothers of Girls Hypertension and Obesity of Girls at Age 18 Years eTable 8. Intergenerational Correlations in Health eTable 9. NFP Postintervention Treatment Effects on Offspring Obesity and Severe Obesity and Maternal Stage 1 and Stage 2 Hypertension—Inverse Probability Weighting and Test for Significance of Sex Differences eTable 10. NFP Postintervention Treatment Effects on Child Obesity and Mother Hypertension—Robustness to Inclusion of Covariates Unbalanced at Baseline eTable 11. Postintervention NFP Effects on Offspring Obesity and Severe Obesity by Offspring Gender—Robustness to Exclusion of Self-Reported BMI Values eTable 12. Postintervention NFP Effects on Maternal Obesity and Severe Obesity by Offspring Gender—Robustness to Exclusion of Self-Reported BMI Values eTable 13. NFP Treatment Effects on Weight-for-Age and Height-for-Age in the First Year of Life eTable 14. Self-Reported Race and Ethnicity by Treatment Status eFigure 1. NFP Treatment Effects on Weight-for-Age in the First Year of Life eFigure 2. NFP Treatment Effects on Height-for-Age in the First Year of Life eReferences [file jamanetwopen-e2351752-s002.pdf]

Supplemental Online Content

Conti G, Smith J, Anson E, et al. Early home visits and health outcomes in low-income mothers and offspring: 18-year follow-up of a randomized clinical trial. *JAMA Netw Open*. 2024;7(1):e2351752. doi:10.1001/jamanetworkopen.2023.51752

eMethods

eTable 1. CONSORT Information for Mothers and Children Enrolled in the Trial Through the 18-Year Follow-Up

eTable 2. Balancing Tests, Baseline Covariate

eTable 3. Ex-Post Power Calculations, Main Outcome

eTable 4. NFP Treatment Effects on Sample Retention

eTable 5. NFP Effects on Length of Gestation, Child Birthweight, Weight at Age 2 Years

eTable 6. NFP Effects on Maternal Blood Pressure at Birth, Pregnancy-Induced Hypertension, and Health Conditions at Child Age 18 Years

eTable 7. Mediation Analysis, Mothers of Girls Hypertension and Obesity of Girls at Age 18 Years

eTable 8. Intergenerational Correlations in Health

eTable 9. NFP Postintervention Treatment Effects on Offspring Obesity and Severe Obesity and Maternal Stage 1 and Stage 2 Hypertension—Inverse Probability Weighting and Test for Significance of Sex Differences

eTable 10. NFP Postintervention Treatment Effects on Child Obesity and Mother Hypertension—Robustness to Inclusion of Covariates Unbalanced at Baseline

eTable 11. Postintervention NFP Effects on Offspring Obesity and Severe Obesity by Offspring Gender—Robustness to Exclusion of Self-Reported BMI Values

eTable 12. Postintervention NFP Effects on Maternal Obesity and Severe Obesity by Offspring Gender—Robustness to Exclusion of Self-Reported BMI Values

eTable 13. NFP Treatment Effects on Weight-for-Age and Height-for-Age in the First Year of Life

eTable 14. Self-Reported Race and Ethnicity by Treatment Status

eFigure 1. NFP Treatment Effects on Weight-for-Age in the First Year of Life

eFigure 2. NFP Treatment Effects on Height-for-Age in the First Year of Life

eReferences

This supplemental material has been provided by the authors to give readers additional information about their work.

© 2024 Conti G et al. *JAMA Network Open.*

eMethods

We conducted a follow-up of mothers and offspring who had participated in a randomized clinical trial of Nurse-Family Partnership.

A. Description of the Intervention

Nurse-Family Partnership © (NFP) is a program of prenatal and infancy/toddler home visiting by nurses for low-income mothers bearing first children.[1] NFP nurses work with mothers, fathers, and other caregivers to address three overarching goals: 1) to improve pregnancy outcomes by promoting women's prenatal health; 2) to improve children's health and development by promoting parents' competent care of their children; and 3) to enhance parents' health and life- course by guiding women to reduce closely spaced subsequent pregnancies, complete their educations, and find work to sustain their family. Nurses link families with needed services and, when possible, involve other family members (especially children's fathers and grandmothers) in the visits. Nurses deliver this program because they have strong interdisciplinary expertise in addressing the multitude of factors that influence maternal and child health; they are trusted by pregnant women and caregivers; and they are the most widely trusted profession in US society. NFP has been tested in a series of randomized clinical trials in the US and other societies and found to produce replicated effects on maternal and child outcomes of clear public health and clinical importance.

The program's success is thought to be enhanced by its alignment with our shared human drive to protect our children[2] and by nurses' developing caring, respectful relationships with mothers and other caregivers that elicit and support that drive.[1] By working with mothers during pregnancy and early years of the child's life, NFP nurses capitalize on the unique opportunities presented at this critical period in human development – when changes in maternal roles and neuroendocrine systems affect maternal and child health over the life-course.[2] Program content and methods are designed to leverage these opportunities.

NFP nurses have caseloads that range between 20-25 families, and schedule visits with women once a week for the first month following registration (prior to the 28th week of gestation), and then every other week until delivery, then once a week for the first six weeks after delivery, every other week through the child's 4th month of life, every three weeks through the 14th month of life, and then every four weeks until the child turns two. Nurses adapt the actual frequency and content of visits to align with families' needs. They visit more frequently when crises occur and adjust downward the frequency of visits when families have fewer needs. Visits last about 75 minutes.

Critically, nurses are selected on the basis of their personal attributes – their ability to be caring, non-judgmental, and respectful.[3] While the program is grounded in thoroughly developed program content, employing home-visiting nurses who are empathic and non-judgmental is an essential feature of the program. As a part of their employment interviews, nurses are asked to reflect on a series of case scenarios designed to reveal their approach to dealing with situations that might lead some to be judgmental. Nurse selection gives priority to their capacity for empathy and respect. This capacity for developing authentic, trusting, caring relationships is fundamental.

In addition, the program is grounded in developmental epidemiology[4] and theories of human-attachment,[5] human-ecology,[6] and self-efficacy.[7]

Developmental Epidemiologic Foundations

Nurse-Family Partnership has been grounded in an understanding of what segments of the population are at greater risk for poor pregnancy outcomes, compromised child health and development, and diminished economic self-sufficiency on the part of parents, and at what periods in human development we have the greatest opportunity to improve maternal and child health. Reviews of this literature led to a focus on women who were bearing their first children and who were either poor, unmarried, or teenaged, given that women with these characteristics generally are at greater risk for poor maternal and child outcomes. In the Memphis trial, the program was offered to women who had at least two of the following characteristics: unmarried, < 12 years of education, or unemployed given that results of the Elmira trial indicated that those women with overlapping needs benefited the most.[1]

The program focuses on improving those parental behaviors and conditions early in life that the epidemiological literature identifies as modifiable factors that predict pregnancy outcomes, child health and development, and maternal life- course. These factors are the targets for behavioral change during pregnancy and yearly years of the child's life; they serve as the behavioral targets for NFP program content. Critically, nurses focus on helping women improve those health behaviors that evidence indicates affect fetal growth and development, including prenatal diet, excessive weight gain, monitoring blood pressure, and encouraging women to work with their primary care providers to address the signs and symptoms of emerging hypertensive disorders, and genital-urinary tract infections. They conduct physical and developmental exams of the child and encourage parents to address concerns with their primary-care providers. This review of the literature is on-going to ensure that program content during pregnancy and the early years of the child's life aligns with state-of-the-art evidence on predictors of maternal and child health during pregnancy, the first two years of life, and over the life-course.

Theoretical Foundations

Human attachment theory emphasizes that responsive early parental caregiving that is attuned to infants' communicative signaling creates a sense of trust on the part of young children that allows them to explore the world with confidence.[5,8] Nurses guide parents to sensitively read and respond to infant communicative signals, provide growth-promoting care of their children, and help parents effectively regulate their toddlers' challenging behavior.

Contextual factors, however, can either support or undermine parents' care of themselves and their children. The stresses of poverty, including having insufficient income to cover survival needs (food, housing, utilities, etc.), and coping with factors like neighborhood crime, can distract parents from protecting themselves and their children. In addition, informal social systems can either amplify or buffer those adversities. Having family-members or friends who are engaged in disruptive, antisocial behavior can contribute to vulnerable parents' engaging in dysfunctional behavior themselves, while having family members and friends who are protective of the mother and child can reduce those forces that distract parents from their caregiving roles. Nurses help parents address their basic needs by linking them with other health and human services, and in engaging other family members and friends in the program, insofar as possible, to build an informal social support system that promotes maternal and child health.

While attachment theory and human ecology theory provide broad frameworks for organizing the realms of behavior addressed by the program, neither provides guidance about how to support

© 2024 Conti G et al. *JAMA Network Open.*

adaptive behavioral change. For this, Bandura's self-efficacy theory[7] has been centrally involved in the program framework. This well-tested approach to behavioral change provides guidance to nurses about how to promote behaviors that support maternal health, fetal growth, early child health and development, and maternal economic self-sufficiency, focusing on what mothers and fathers are already doing well. The basic idea is that individuals will change their behavior to the extent that they believe change is important and they have confidence in their ability to make needed changes. Nurses guide mothers and fathers to reflect on what is important to them and to identify small, achievable objectives that will help them accomplish their goals.

Implementation Data

Nurses enter data into a program information system that enables them to monitor delivery of the program and invoke continuous quality improvement efforts as well as more substantial research undertakings to improve the program in practice. The information system characterizes the families served, including baseline health, behavior, and social factors that support or undermine parents' abilities to protect themselves and their children.[9] It is also designed to enable monitoring the degree to which the program is delivered with fidelity to the model.

© 2024 Conti G et al. *JAMA Network Open.*

B. Definitions of Outcomes Examined in this Study

For this study, we examined a set of outcomes that were not identified in the original trial design as primary outcomes. For this set of analyses, conducted in the current study, we focused attention on obesity and hypertension measured at offspring ages 12 and 18 as these outcomes were measured directly with standardized instruments, with the exception of obesity, which included a small number of self-reported weights and heights, as specified below. Moreover, obesity and hypertension measured at these ages have a stronger relationship with the development of chronic disease. In addition, we examined a set of additional variables to place the obesity and hypertension outcomes in developmental context. We refer to obesity and hypertension as the primary foci of this study. All variables examined in this study, however, are exploratory.

Outcomes that are Primary Foci of this Study

At child ages 12 and 18 years, study staff assessed maternal and child height and weight with shoes removed using a Healthometer device (Model 402KLS), and blood pressure using an OMRON digital blood pressure monitor (HEM-712C). Blood pressure measurements were repeated after 10 minutes if the initial reading exceeded normal ranges; the last reading was recorded.

- Child obesity at ages 12 and 18 is defined as a standardized BMI at or above the 95th percentile of the BMI-for-age distribution (2000 CDC growth charts)[10].
- Child severe obesity at ages 12 and 18 is defined as a standardized BMI at or above the 99th percentile of the BMI-for-age distribution (2000 CDC growth charts).[10]
- Mother obesity at child ages 12 and 18 is defined as having a BMI at or above 30.[10]
- Mother severe obesity at child ages 12 and 18 is defined as having a BMI at or above 35.[10]
- Child stage 1 hypertension at ages 12 and 18 is defined as having systolic or diastolic blood pressure above the 95th percentile of the gender-specific age-standardized distribution.[11]
- Child stage 2 hypertension at ages 12 and 18 is defined as having systolic or diastolic blood pressure above the 99th percentile of the gender-specific age-standardized distribution.[11]
- Mother stage 1 hypertension is defined as systolic blood pressure ≥ 130 or diastolic blood pressure ≥ 80.[12]
- Mother stage 2 hypertension is defined as systolic blood pressure ≥ 140 or diastolic blood pressure ≥ 90.[12]

Other Outcomes

We examined a set of other outcomes to place the findings related to obesity and hypertension in developmental and contemporaneous context. They are listed below:

- Gestational age and birthweight are extracted from birth records.
- Preterm is gestational age at birth <37 weeks.
- Low birth weight is <2,500 grams.
- High birth weight is >4,000 grams.
- Pregnancy-Induced Hypertension and Mean Arterial Pressure at birth were extracted from the hospital records.

© 2024 Conti G et al. *JAMA Network Open.*

- Weight and height during the first year of life of the child were measured during the developmental screenings; z-scores have been computed using the 2000 CDC Growth Reference Charts.[10] Note that we have computed growth curves for these outcomes in part because of missing data for some time-points for these variables.
- Child overweight at 2 years was reported by the mother (or caregiver) in response to the following question (no. 51) from the 1988 version of the Child Behavior Checklist:[13] For each item that describes the child now or with the past 2 months, please circle the 2 if the item is ***very true*** or ***often true*** of the child. Circle the 1 if the item is ***somewhat or sometimes true*** of the child. If the item is ***not true*** of the child, circle the 0. Please answer all items as well as you can, even if some do not seem to apply to the child:

  0=not true
  1=somewhat or sometimes true
  2=very true or often true

  Items 1 and 2 were combined to create a single yes/no variable

- At the 18-year interview, mothers were asked about whether they had any of the following health conditions in the following format:

Have you ever been told that you have any of the following? Circle yes under the "You" column for those that you have been told you have.

	**You**
Diabetes	Yes.......1
Heart Disease	Yes.......1
High Blood Pressure	Yes....... 1
High Cholesterol	Yes....... 1
Cancer	Yes....... 1
Asthma	Yes....... 1
Cardiovascular Disease (i.e. stroke)	Yes....... 1
Migraines/Headaches	Ye.........1
Respiratory (i.e. emphysema, COPD)	Yes....... 1
Neuromuscular Disorders (i.e. back pain, arthritis)	Yes....... 1
Sleep Apnea	Yes....... 1
Seasonal Allergies	Yes....... 1
Arthritis in hands, knees or hip	Yes....... 1
Thyroid Problems	Yes........1
Kidney Problems	Yes....... 1
Bowel Problems (i.e. constipation/diarrhea)	Yes....... 1
Skin Problems (i.e. acne, dermatitis)	Yes....... 1
Stomach Problems/Acid Reflux (i.e. irritable bowel, ulcers)	Yes....... 1
Bladder Problems	Yes....... 1
Seizure Disorder	Yes....... 1
Chronic Pain	Yes....... 1
Memory Problems	Yes....... 1
Liver Disease	Yes....... 1

© 2024 Conti G et al. *JAMA Network Open.*

C. Statistical Methods

Linear regression models

Consider the following model:

$$y_i = g(t_i, \boldsymbol{r'}_i, \boldsymbol{x'}_i, u_i) \qquad (1)$$

where y_i is an observed outcome of participant i, t is a binary indicator taking value 1 if the woman is allocated to the treatment group (0 otherwise), $\boldsymbol{r'}_i$ is a vector of pre-program variables included in the randomization protocol, $\boldsymbol{x'}_i$ is a vector of baseline controls, and u_i is a disturbance term. The model (1) takes a linear specification for continuous outcomes y_i:

$$y_i = \beta_0 + \beta_1 t_i + \beta_2 \boldsymbol{r'}_i + \beta_3 \boldsymbol{x'}_i + u_i \qquad (2)$$

and a logit specification for binary outcomes $y_i \in \{0,1\}$:

$$\Pr(y_i = 1 | t_i, \boldsymbol{r'}_i, \boldsymbol{x'}_i) = \frac{\exp(\beta_0 + \beta_1 t_i + \beta_2 \boldsymbol{r'}_i + \beta_3 \boldsymbol{x'}_i)}{1 + \exp(\beta_0 + \beta_1 t_i + \beta_2 \boldsymbol{r'}_i + \beta_3 \boldsymbol{x'}_i)} \qquad (3)$$

The objective is to estimate average treatment effects for mothers' and children's outcomes by gender, and to test the null hypothesis of no treatment effects, by exploiting the fact that, because of randomization, counterfactual outcomes are independent of the treatment status t_i. Variables included in the vector $\boldsymbol{r'}_i$ are: race (African-American or other races), age at intake (<17, 17-18, ≥19 years), gestational age at intake (<20 or ≥20 weeks), employment status of the head of the household (employed or unemployed), and geographic region of residence (four regions).

One common issue to deal with in randomized trials is the possibility that the randomization protocol has been compromised, which might induce a spurious correlation between treatment status and outcomes. In Table S2A and S2B, we perform balancing tests by gender of a full set of 74 baseline covariates; we see that, after accounting for multiple hypothesis testing, very few are imbalanced, in particular mothers of males in the treatment group have higher pre-pregnancy BMI, and mothers of females in the treatment group are

© 2024 Conti G et al. *JAMA Network Open.*

more economically disadvantaged. To account for this potential issue, we use the post double selection lasso procedure,[17] so to select the vector of baseline controls $\boldsymbol{x'}_{\boldsymbol{i}}$, among a set of 130 variables: we start from the 74 listed in Tables S2A and S2B, we replace the missing observations with zeros and add binary indicators equal to 1 if the variable is missing, we add the squares of the continuous variables, standardize them, and we drop any pair of collinear variables, following a standard procedure.[11]

Model (2) is then supplemented by the following linear model:

$$t_i = \gamma_0 + \gamma_1 \boldsymbol{r'}_{\boldsymbol{i}} + \gamma_2 \boldsymbol{x'}_{\boldsymbol{i}} + \varepsilon_i \tag{4}$$

while model (3) is supplemented by the following logit model

$$\Pr(t_i = 1 | \boldsymbol{r'}_i, \boldsymbol{x'}_i) = \frac{\exp(\gamma_0 + \gamma_1 t_i + \gamma_2 \boldsymbol{r'}_{\boldsymbol{i}} + \gamma_3 \boldsymbol{x'}_{\boldsymbol{i}})}{1 + \exp\left(\gamma_0 + \gamma_1 t_i + \gamma_2 \boldsymbol{r'}_{\boldsymbol{i}} + \gamma_3 \boldsymbol{x'}_{\boldsymbol{i}}\right)} \tag{5}$$

where ε_i is a disturbance and all the other terms are defined as above.

Lasso-based model selection is then applied to each equation to select the control variables. For the linear models (2) and (4), we partial the variables used in the randomization protocol prior to the lasso estimation. In the logit model case, instead, we simply do not allow the Lasso selection process to drop these variables (i.e., these variables will appear as regressors in the post-selection stage). In both cases, we allow the penalty loadings to vary among the regressors. We can then estimate β_1 by a least squares regression (in the linear model case) or a logistic regression (in the logit model case) of y_i on t_i and the union of the selected controls. Inference on β_1 may then be performed using conventional methods for inference about parameters estimated by least squares. The treatment effect for binary outcomes is then computed as the adjusted relative risk (RR):

$$RR = \frac{\frac{1}{N}\sum_i \Pr(y_i = 1 | t_i = 1, \boldsymbol{r'}_i, \boldsymbol{x'}_i)}{\frac{1}{N}\sum_i \Pr(y_i = 1 | t_i = 0, \boldsymbol{r'}_i, \boldsymbol{x'}_i)}$$

When testing multiple hypotheses simultaneously, standard statistical techniques will lead to over-rejection of null hypotheses unless the multiplicity of the testing framework is explicitly considered. Here we

© 2024 Conti G et al. *JAMA Network Open.*

do so by using the Romano-Wolf procedure,[17] which (asymptotically) controls the familywise error rate (FWER), that is, the probability of rejecting at least one true null hypothesis in a family of hypotheses under test. This correction is considerably more powerful than other multiple testing procedures such as Bonferroni and Holm, given that it takes into account the dependence structure of the test statistics by resampling from the original data. We implement this procedure by using 5,000 bootstrap replications.

Another potential issue when analyzing data from randomized trial is the potential loss at follow-up. Compared with similar long-term follow-ups of early interventions,[18] sample retention is very high for a study of this kind. In Table S4 we present results from least squares regressions of a binary indicator for retention at a particular wave/data collection sweep on a constant and on a binary indicator for treatment status. The results show that sample retention ranges from a minimum of 73.5% (age 12 maternal blood pressure measurement) to a maximum of 99%-100% for the birth sweeps of the female and male samples, respectively. Crucially, treatment does not predict sample retention. Nonetheless, we test the robustness of our results on the outcomes that are the primary foci of this study at ages 12 and 18 (Table S4) to accounting for loss at follow-up by implementing an inverse probability weighting estimator as follows: we first use lasso to select the determinants of retention at each wave/data collection sweep among the set of baseline covariates $\boldsymbol{x'_i}$, we then use a logit model with the selected controls to estimate the probability of retention, and we use the inverse of the predicted probability as weight in the analyses.

Quantile regression models

We estimate quantile regression models[19] to study the effect of the treatment along the outcome distribution for the continuous outcomes of body mass index and blood pressure (Figure 1). While usual OLS and logistic regressions estimate treatment effects on the mean of the outcome, quantile regressions allow us to estimate the treatment effect of FNP on different quantiles of the outcome distribution. Formally, quantile regression estimates the following model:

$$Q_\tau(y_i|\boldsymbol{x'}_i) = \alpha_\tau + \beta\boldsymbol{x'}_i,$$

© 2024 Conti G et al. *JAMA Network Open.*

(6)

where $Q_\tau(y_i|\boldsymbol{x'}_i)$ is the τth quantile of the distribution of y_i conditional on $\boldsymbol{x'}_i$, with $0 < \tau < 1$. For instance, $Q_{0.1}(y_i|\boldsymbol{x'}_i)$ denotes the 10th percentile (or 1st decile) of y_i as a function of $\boldsymbol{x'}_i$.

In our analysis, we first residualize the outcome variables y_i (of the variables used in the randomization protocol and the controls selected with the lasso) and we then estimate simultaneous-quantile regression for each decile of the outcome distribution using the treatment dummy as the only regressor of equation (6). Standard errors are computed using 1000 bootstrap replications to obtain an estimate of the variance-covariance matrix.

Growth Models

For the longitudinal data on child growth, we estimate the following growth curve model, separately for boys and girls:

$$y_{ij}^g = \beta_0^g + \beta_1^g T_j + \beta_2^g age_{ij} + \beta_3^g T_j \times age_{ij} + \beta_4^g age_{ij}^2 + \beta_5^g T_j \times age_{ij}^2 + \beta_6^g age_{ij}^3 + \beta_7^g T_j \times age_{ij}^3 +$$
$$\dots + u_{0j,g} + u_{1j,g} age_{ij} + u_{2j,g} age_{ij}^2 + \epsilon_{ij}^g \qquad (3)$$

where y_{ij}^g is outcome *y* for child *j* of gender *g* (*g*=0 is female and *g*=1 male) and age *i*, age is measured in months, growth is modelled as a cubic and treatment is allowed to impact growth. Here we only control for the strata variables used in the randomization protocol. Hence, by testing *H0:* $\beta_3^g = 0, \beta_5^g = 0$ and $\beta_7^g = 0$ we can check if the average growth rate is significantly different between treatment and control. We model overall mean growth as cubic in age and allow for child-specific random effects: a random intercept $u_{0j,g}$, which represents each child's vertical shift from the overall mean, and a quadratic random slope in age $u_{1j,g}$ and $u_{2j,g}$, which represent each child's deviation from the overall mean quadratic growth rate. These random effects have unrestricted covariance:

$$\Sigma^g = \mathrm{Var}\begin{bmatrix} u_{0j,g} \\ u_{1j,g} \\ u_{2j,g} \end{bmatrix} = \begin{bmatrix} \sigma_{u0,g}^2 & \sigma_{01,g} & \sigma_{02,g} \\ \sigma_{01,g} & \sigma_{u1,g}^2 & \sigma_{12,g} \\ \sigma_{02,g} & \sigma_{12,g} & \sigma_{u2,g}^2 \end{bmatrix}$$

The estimated coefficients are shown in Table S5, while the graphs in Figures S1 and S2 show the corresponding fitted values.

© 2024 Conti G et al. *JAMA Network Open.*

Mediation Analysis

Lastly, we use the conditional decomposition method developed by Gelbach[19] to explain how much various mediators account for the effect of the treatment on the outcome. This method is based on the comparison between β_1 in the model in equation (1) and μ_1 in the following model, augmented with the mediators $\boldsymbol{m'_i}$:

$$y_i = \mu_0 + \mu_1 t_i + \mu_2 \boldsymbol{r'_i} + \mu_3 \boldsymbol{x'_i} + \mu_4 \boldsymbol{m'_i} + u_i \quad (1)$$

We report in Table S6 the results of the mediation analysis (with one or two mediators) for the significant outcomes that are the primary foci of this study.

© 2024 Conti G et al. *JAMA Network Open.*

eTable 1. CONSORT Information for Mothers and Children Enrolled in the Trial Through the 18-Year Follow-Up

**Eligible Subjects Invited to Participate***	1289				
Number Refused	151				
Number Randomized*	1138				
**Treatment Group Assignment**	**1 a**	**2 b**	**3 c**	**4 d**	**Total (TX 2 & 4)**
Number Allocated to Each Treatment [e]	166	**514** [f]	230	**228**	742
Miscarriages (mothers not followed)	6	19	6	8	27
Stillbirths (mothers not followed)	0	5	3	2	7
Mother declined participation after randomly assigned		14		11	25
**12-year follow up**					
**Mothers**					
Child death before age 2 (mothers not followed)		7		1	8
Maternal Deaths (through first born age 12)		8		2	10
**Available mothers for 12-year follow up**		**461**		**204**	**665**
Completed 12-year Maternal interview		407 (79%)		187 (82%)	594 (80%)
Mom pregnant at 12-year interview (data not used)		11		6	17
Completed 12-year BMI assessment (measured)		341		157	498
Completed 12-year BMI assessment (self-report)		44		22	66
12-year BMI measured & self-report)		385		179	564
Completed 12-year BP assessment (measured)		348		160	508
**Child**					
Infant and childhood deaths (through first born age 12)		10		2	12
**Available children for 12 year follow up**		**466**		**205**	**671**
Completed 12-year Child interview		398 (77%)		180 (79%)	578 (78%)
Completed 12-year BMI assessment (measured)		388		175	563
Completed 12-year BMI assessment (self-report)		9		5	14
12-year BMI measured & self-report)		397		180	577
Completed 12-year BP assessment (measured)		392		177	569
**18-year follow up**					
**Mothers**					
Maternal Deaths (between first born age 12 and 18)		5		1	6
**Available mothers for 18-year follow up**		**456**		**203**	**659**
Completed 18-year Maternal interview		426 (83%)		192 (84%)	618 (83%)
Mom pregnant at 18-year interview (data not used)		4		3	7
Completed 18-year BMI assessment (measured)		399		175	574
Completed 18-year BMI assessment (self-report)		17		8	25
18-year BMI measured & self-report)		416		183	599
Completed 18-year BP assessment (measured)		409		184	593
**Child**					
Child declined participation before age 18		1		0	1
**Available children for 18 year follow up**		**465**		**205**	**670**
Completed 18-year Child interview		435 (85%)		194 (85%)	629 (85%)
Child pregnant at 18-year interview (data not used)		10		4	14
Completed 18-year BMI assessment (measured)		404		175	579
Completed 18-year BMI assessment (self-report)		16		11	27
18-year BMI measured & self-report)		420		186	606
Completed 18-year BP assessment (measured)		412		185	597

* The women who accepted participation completed informed consent and were randomized to one of four treatment conditions following a procedure that concealed assignment from individuals involved in gathering participant data[18]. Sample size and assignment ratios were derived from statistical power calculations in the original phase of the trial.
a Treatment 1 - Prenatal transportation
b Treatment 2 - Prenatal transportation + developmental screening and referral
c Treatment 3 - Prenatal transportation + developmental screening and referral + prenatal nurse home visits
d Treatment 4 - Prenatal transportation + developmental screening and referral + prenatal and infant/toddler nurse home visits
e We assigned twice as many participants to the T2 control condition as T4 Nurse-Visited group to minimize costs, given that program costs were paid for with research dollars. Groups 1 and 3 were included in the original phase of the trial to meet statistical power requirements for estimating prenatal effects.
f Note that one mother was enrolled and randomized twice by mistake following a miscarriage. We included her only once with her original assignment to the control group.
NOTE at 12-year, n=17 moms and at 18-year, n=7 moms and n=14 children were currently pregnant; BMI and BP were coded as missing.

© 2024 Conti G et al. *JAMA Network Open.*

eTable 2. Balancing Tests, Baseline Covariate

	Mothers of Girls				Mothers of Boys			
**Baseline**	**MC**	**MT**	**p-v**	**MHT**	**MC**	**MT**	**p-v**	**MHT**
RP: Race (=1 if black)	0.916	0.911	0.877	0.997	0.928	0.890	0.247	0.804
RP: Age 13-16	0.249	0.321	0.153	0.610	0.293	0.309	0.762	0.998
RP: Age 17-18	0.321	0.268	0.309	0.796	0.338	0.300	0.488	0.976
RP: Age 19-34	0.430	0.411	0.736	0.992	0.369	0.391	0.700	0.997
RP: Gestational age <20 weeks	0.687	0.732	0.385	0.824	0.651	0.636	0.795	0.998
RP: Head of household employed	0.605	0.482	**0.030**	0.144	0.518	0.509	0.876	0.998
RP: Region: Hollywood	0.173	0.232	0.185	0.654	0.205	0.200	0.917	0.998
RP: Region: Bisson	0.229	0.179	0.281	0.796	0.185	0.236	0.261	0.833
RP: Region: Cawthon	0.317	0.312	0.928	0.997	0.321	0.291	0.568	0.990
RP: Region: Inner City	0.281	0.277	0.932	0.997	0.289	0.273	0.751	0.998
Income and Poverty								
Number of people in the household	4.3	4.6	0.226	0.915	4.2	4.7	**0.037**	0.293
Number of rooms	4.8	4.7	0.331	0.965	4.8	5.0	0.285	0.957
Housing density (#people/#rooms)	0.951	1.049	0.113	0.697	0.931	0.999	0.223	0.934
Discretionary household income	1.981	-0.162	**0.006**	**0.045**	1.403	0.281	0.147	0.790
Composite HH poverty	98.9	102.4	**0.003**	**0.017**	100.0	101.4	0.219	0.934
HH income Less Than $3000	0.617	0.714	**0.074**	0.519	0.677	0.709	0.552	0.999
HH income $3000 - $6999	0.165	0.187	0.607	0.985	0.145	0.100	0.244	0.941
HH income $7000- $10999	0.109	0.080	0.405	0.974	0.093	0.109	0.632	0.999
HH income Greater Than $11000	0.109	0.018	**0.003**	**0.021**	0.085	0.082	0.928	1.000
% of Census Tract Below Poverty	32.62	36.34	0.124	0.697	36.46	34.15	0.312	0.965
HH income increased a lot last 6mo	0.052	0.045	0.755	0.985	0.053	0.064	0.684	0.999
HH income somewhat increased last 6mo	0.145	0.143	0.954	0.985	0.158	0.118	0.320	0.965
HH income stayed the same last 6mo	0.516	0.536	0.731	0.985	0.565	0.518	0.413	0.978
HH income somewhat decreased last 6mo	0.185	0.152	0.437	0.974	0.098	0.173	**0.044**	0.329
HH income decreased a lot last 6mo	0.101	0.125	0.495	0.974	0.126	0.127	0.974	1.000
No problems in current HH income	0.314	0.339	0.643	0.985	0.329	0.336	0.896	1.000
Minor problems in current HH income	0.593	0.527	0.243	0.923	0.546	0.518	0.625	0.999
Major problems in current HH income	0.093	0.134	0.240	0.923	0.124	0.145	0.589	0.999
Receives Food Stamps	0.145	0.116	0.458	0.974	0.136	0.145	0.823	0.999
Received Medicaid	0.308	0.359	0.360	0.965	0.375	0.385	0.867	0.999
Socioeconomic Status								
Highest Grade Completed	10.4	10.1	0.188	0.570	10.2	10.1	0.584	0.937
In School	0.540	0.598	0.307	0.705	0.635	0.527	**0.056**	0.186
Graduated High School	0.321	0.330	0.865	0.990	0.285	0.300	0.775	0.937
Employed	0.080	0.053	0.364	0.713	0.076	0.082	0.858	0.937
Ever worked	0.637	0.575	0.278	0.701	0.587	0.554	0.583	0.937
Homeowner	0.318	0.306	0.821	0.990	0.298	0.354	0.290	0.736
House moves in the last 12 months	1.14	1.10	0.823	0.990	1.13	1.00	0.390	0.816
Fertility								
Age of First Menstrual Period	12.23	12.30	0.717	0.969	12.39	12.25	0.479	0.938
Age of First Sex	15.05	15.12	0.745	0.969	15.05	15.01	0.864	0.938
Any previous pregnancies	0.212	0.179	0.463	0.927	0.173	0.209	0.423	0.932
Any previous abortion	0.211	0.170	0.360	0.859	0.157	0.200	0.314	0.851
Abortion considered	0.270	0.196	0.134	0.457	0.285	0.218	0.186	0.684
Was pregnancy planned	0.129	0.234	**0.012**	**0.054**	0.109	0.118	0.624	0.938
Wanted a baby sometimes	0.726	0.803	0.115	0.428	0.671	0.727	0.287	0.851
Desired Fertility	1.722	1.786	0.501	0.927	1.847	1.791	0.590	0.938
Belief in Preventive Methods	0.310	0.324	0.795	0.969	0.277	0.245	0.533	0.938

© 2024 Conti G et al. *JAMA Network Open.*

eTable 2. Balancing Tests, Baseline Covariates (Ctd.)

	Mothers of Girls				Mothers of Boys			
**Baseline**	**MC**	**MT**	**p-v**	**MHT**	**MC**	**MT**	**p-v**	**MHT**
Personal Relationships								
Married	0.012	0.009	0.794	0.981	0.024	0.018	0.727	0.987
Has husband or boyfriend	0.778	0.785	0.889	0.981	0.791	0.790	0.979	0.987
Social support - Husband	3.996	3.994	0.987	0.981	4.037	4.070	0.726	0.987
Mother alive	0.943	0.955	0.644	0.950	0.944	0.954	0.675	0.987
Live with Mother	0.594	0.741	**0.007**	**0.011**	0.691	0.664	0.612	0.987
Social support – Mother	3.933	4.076	**0.092**	0.242	4.068	4.098	0.660	0.987
Parents lived apart before 13th birthday	0.692	0.664	0.592	0.950	0.676	0.706	0.572	0.987
Cognitive and mental health								
Intellectual Functioning (Shipley IQ)	96.42	96.37	0.965	0.999	96.12	96.63	0.670	0.932
Mastery	100.3	99.05	0.285	0.741	99.99	99.80	0.877	0.972
Positive Affect Scale	-0.013	-0.057	0.708	0.988	0.066	-0.068	0.262	0.691
Personal Beliefs Scale	0.021	-0.155	0.151	0.454	0.022	0.019	0.978	0.983
Self-Efficacy	100.7	100.6	0.964	0.999	99.48	98.48	0.404	0.840
Anxiety	2.591	2.629	0.667	0.988	2.537	2.618	0.380	0.840
Depression	2.213	2.196	0.839	0.997	2.143	2.217	0.383	0.840
Mental Health	99.78	99.26	0.653	0.987	100.6	99.03	0.193	0.559
High Psychological Resources	0.456	0.464	0.879	0.997	0.514	0.427	0.130	0.439
Physical Health								
Pre pregnancy Height (cm)	164.3	164.3	0.993	1.000	164.5	163.5	**0.094**	0.349
Pre pregnancy Weight (kg)	62.85	61.58	0.419	0.894	61.19	63.77	0.140	0.415
Pre pregnancy BMI	23.30	22.78	0.333	0.787	22.44	23.89	**0.014**	**0.063**
Pre pregnancy Normal Weight	0.705	0.705	0.872	0.997	0.717	0.589	0.735	0.945
Pre pregnancy Overweight	0.270	0.250	0.735	0.996	0.217	0.355	**0.095**	0.349
Pre pregnancy Obese	0.095	0.053	0.999	1.000	0.090	0.140	**0.018**	**0.078**
Weight at Recruitment	67.64	67.00	0.697	0.994	67.24	70.88	**0.006**	**0.027**
Optimal Perceived Weight Gain (kg)	10.60	11.74	0.183	0.622	11.28	11.04	0.161	0.440
Gestational Age at recruitment	16.36	16.26	0.195	0.622	16.74	16.96	0.826	0.945
Age at recruitment	18.60	18.44	0.655	0.994	18.28	18.14	0.674	0.945
Health Behaviours								
Ever smoked regularly	0.141	0.170	0.352	0.551	0.116	0.127	0.871	0.861
Smoked in past 3 days	0.093	0.125	0.484	0.682	0.096	0.091	0.772	0.861
Average no. cigarettes/day	2.056	2.348	0.680	0.840	1.566	1.918	0.556	0.829
Drank alcohol past 14 days	0.032	0.062	0.185	0.358	0.044	0.027	0.447	0.751
Used marijuana past 2 wks	0.012	0.009	0.791	0.840	0.016	0.009	0.604	0.842
Health Conditions								
Any STDS	0.333	0.348	0.783	0.873	0.321	0.409	0.108	0.291
No. of STDs	0.390	0.393	0.969	0.973	0.442	0.491	0.547	0.833
Gardnerella infections	0.069	0.098	0.343	0.676	0.084	0.118	0.314	0.699
Yeast infection	0.085	0.062	0.456	0.678	0.084	0.064	0.501	0.833
UTI	0.069	0.107	0.222	0.589	0.124	0.118	0.867	0.855
Child rearing:								
Child Abuse Attitudes (Bavolek)	99.76	100.9	0.208	0.232	99.71	100.7	0.293	0.283
Raising children scale (factor score)	0.029	-0.072	0.400	0.372	0.035	-0.073	0.369	0.318

Note. MC=mean control; MT=mean treatment; RP=Randomization Protocol; p-v=p-value for the two-sided difference in means between treated and control (conditional on the variables used in the RP); MHT=two-sided multiple hypothesis testing p-value using the step-down methodology of Romano and Wolf (2005) with 1,000 bootstrap replications for blocks of outcomes (blocks headings underlined).

© 2024 Conti G et al. *JAMA Network Open.*

eTable 3. Ex-Post Power Calculations, Main Outcome

Outcome	NC	NT	Mean C	Mean T	SD C	SD T	Power (alpha=0.05)	Power (alpha=0.10)
Female offspring								
Obese at age 12y	199	87	0.317	0.149	0.466	0.359	0.912	0.952
Obese at age 18y	213	94	0.315	0.223	0.465	0.419	0.403	0.527
Obese at age 12y and 18y	187	82	0.251	0.11	0.435	0.315	0.849	0.911
Severe obese at age 12y	200	87	0.18	0.069	0.385	0.255	0.820	0.891
Severe obese at age 18y	214	93	0.182	0.108	0.387	0.311	0.426	0.551
Severe obese at age 12y and 18y	187	82	0.123	0.024	0.329	0.155	0.918	0.956
Mothers of female offspring								
Stage 1 Hypertension at child 12y	174	79	0.609	0.43	0.489	0.498	0.759	0.846
Stage 1 Hypertension at child 18y	203	90	0.813	0.667	0.391	0.474	0.726	0.820
Stage 1 Hypertension at child 12y and 18y	164	74	0.567	0.365	0.497	0.485	0.839	0.904
Stage 2 Hypertension at child 12y	175	78	0.331	0.103	0.472	0.305	0.996	0.998
Stage 2 Hypertension at child 18y	203	90	0.507	0.436	0.501	0.501	0.199	0.297
Stage 2 Hypertension at child 12y and 18y	165	73	0.267	0.055	0.444	0.229	0.998	0.999

Note. This table presents the ex-post power calculations for a two-sample means test, using the sample size and means/standard deviations differences in the trial treatment and control groups, for alpha=0.05.

© 2024 Conti G et al. *JAMA Network Open.*

eTable 4. NFP Treatment Effects on Sample Retention

Outcome: Retention in Sweep	Female Sample		Male Sample	
	**Treatment**	**Control Mean**	**Treatment**	**Control Mean**
Child age 18 interview (height/weight recorded)	-0.0242	0.863***	0.0131	0.823***
	(0.0411)	(0.0218)	(0.0429)	(0.0242)
Child age 18 interview (blood pressure recorded)	-0.00319	0.851***	0.0150	0.803***
	(0.0408)	(0.0226)	(0.0447)	(0.0253)
Mother age 18 interview (height/weight recorded)	-0.0447	0.839***	0.0232	0.831***
	(0.0448)	(0.0233)	(0.0413)	(0.0238)
Mother age 18 interview (blood pressure recorded)	-0.0197	0.823***	0.0353	0.819***
	(0.0448)	(0.0242)	(0.0416)	(0.0245)
Child age 12 interview (height/weight recorded)	-0.0215	0.807***	0.0492	0.787***
	(0.0463)	(0.0251)	(0.0439)	(0.0260)
Child age 12 interview (blood pressure recorded)	-0.0184	0.795***	0.0391	0.779***
	(0.0471)	(0.0256)	(0.0453)	(0.0264)
Mother age 12 interview (height/weight recorded)	-0.0104	0.787***	0.0338	0.739***
	(0.0473)	(0.0260)	(0.0488)	(0.0279)
Mother age 12 interview (blood pressure recorded)	-0.00635	0.783***	0.0733	0.763***
	(0.0474)	(0.0262)	(0.0445)	(0.0270)
Age 2 Interview	-0.00147	0.707***	0.0456	0.691***
	(0.0520)	(0.0289)	(0.0514)	(0.0294)
Child birth outcomes	-0.00628	0.944***	0.00168	0.944***
	(0.0272)	(0.0146)	(0.0262)	(0.0146)
Mother birth outcomes	-0.0447	0.920***	0.0387	0.880***
	(0.0358)	(0.0173)	(0.0334)	(0.0207)

Note: The table shows results from ordinary least squares regressions of each outcome (binary variable of retention in a certain sweep) on the treatment indicator and a constant. The coefficient on the constant term represents the mean for the control group. n/a=there is no loss at that sweep. ***:p<0.01; **:p<0.05; *:p<0.10.

© 2024 Conti G et al. *JAMA Network Open.*

eTable 5. NFP Effects on Length of Gestation, Child Birthweight, Weight at Age 2 Years

**Outcome**	**N (%)**	**Control Mean (SD)**	**Treatment Mean (SD)**	**LSMD/RR (95% CI)**	***p-value***	**MHT *p-value***
Gestational age at birth (weeks)						
Overall sample	736 (100)	38.14 (5.08)	38.48 (4.85)	0.286 (-0.293 - 0.864) ‡	*0.333*	*0.490*
Females	358 (49)	39.11 (2.23)	38.70 (3.88)	-0.354 (-1.054 - 0.346) ‡	*0.322*	*0.545*
Males	359 (49)	38.54 (3.55)	39.33 (2.54)	0.755 (0.150 - 1.359) ‡	***0.015***	***0.039***
Preterm Birth						
Overall sample	736 (100)	0.18 (0.38)	0.11 (0.32)	0.663 (0.455 - 0.965)	***0.018***	*0.095*
Females	356 (48)	0.14 (0.34)	0.10 (0.30)	0.753 (0.402 - 1.412)	*0.348*	*0.545*
Males	359 (49)	0.17 (0.38)	0.09 (0.29)	0.54 (0.280 - 1.042)	***0.028***	*0.166*
Weight at birth (grams)						
Overall sample	717 (100)	3018.74 (659.11)	3064.5 (676.27)	53.13 (-46.31 - 152.58) ‡	*0.295*	*0.490*
Females	357 (50)	3052.25 (553.81)	2934 (698.62)	-104.8 (-243.89 - 34.26) ‡	*0.140*	*0.298*
Males	359 (50)	2997.49 (726.41)	3197 (628.43)	191.37 (55.01 - 327.75) ‡	***0.006***	***0.014***
Low Weight at birth						
Overall sample	717 (100)	0.17 (0.37)	0.12 (0.33)	0.727 (0.487 - 1.085)	*0.094*	*0.352*
Females	356 (50)	0.17 (0.38)	0.14 (0.35)	0.872 (0.519 - 1.465)	*0.595*	*0.603*
Males	359 (50)	0.17 (0.37)	0.10 (0.30)	0.615 (0.341 - 1.11)	*0.070*	*0.225*
High Weight at birth						
Overall sample	717 (100)	0.04 (0.20)	0.02 (0.15)	0.565 (0.217 - 1.472)	*0.183*	*0.490*
Females	718 (50)	0.05 (0.22)	0.00 (0.00)	-	-	-
Males	359 (50)	0.03 (0.18)	0.05 (0.21)	1.38 (0.46 - 4.141)	*0.586*	*0.809*
Overweight at 2y						
Overall sample	662 (100)	0.11 (0.32)	0.08 (0.28)	0.709 (0.424 - 1.186)	*0.160*	*0.479*
Females	327 (49)	0.13 (0.34)	0.06 (0.33)	0.405 (0.170 - 0.966)	***0.013***	*0.111*
Males	334 (50)	0.09 (0.29)	0.11 (0.31)	1.068 (0.546 - 2.089)	*0.849*	*0.842*

Note. The columns present the following information: (i) outcome analyzed; (ii) sample size (N); (iii) mean of the control group; (iv) mean of the treatment group; (v) least squares mean difference (LSMD) for continuous outcomes (superscript ‡), adjusted relative risk for binary variables (estimated through a logistic regression), and 95% confidence interval in parenthesis; (vi) two-sided asymptotic p-value for the null hypothesis that the treatment effect is zero; (vii) two-sided multiple hypothesis testing (MHT) p-value using the step-down methodology of Romano and Wolf (2005).[17] The adjusted relative risk of “high weight at birth” for female offspring cannot be estimated because there are no observations with high birth weight in the treatment group of this subsample.

© 2024 Conti G et al. *JAMA Network Open.*

eTable 6. NFP Effects on Maternal Blood Pressure at Birth, Pregnancy-Induced Hypertension, and Health Conditions at Child Age 18 Years

Outcome	N (%)	Control Mean (SD)	Treatment Mean (SD)	LSMD/RR (95% CI)	*p-value*	MHT *p-value*
Mean Arterial Pressure at birth						
Overall sample	660 (100)	93.36 (12.84)	91.23 (10.81)	-2.226 (-4.091 - -0.362) ‡	***0.019***	*0.053*
Mothers of females	328 (50)	93.55 (13.00)	89.45 (10.30)	-4.064 (-6.727 - -1.401) ‡	***0.003***	***0.007***
Mothers of males	329 (50)	93.3 (12.68)	93.06 (11.06)	-0.442 (-3.130 - 2.247) ‡	*0.748*	*0.936*
Pregnancy-Induced Hypertension						
Overall sample	711 (100)	0.20 (0.40)	0.15 (0.36)	0.754 (0.526 - 1.080)	*0.102*	*0.195*
Mothers of females	354 (50)	0.21 (0.41)	0.14 (0.34)	0.623 (0.368 - 1.055)	*0.052*	*0.054*
Mothers of males	356 (50)	0.19 (0.39)	0.16 (0.37)	0.86 (0.524 - 1.410)	*0.537*	*0.885*
Any Health Condition, child 18y						
Overall sample	616 (100)	0.71 (0.46)	0.63 (0.49)	0.879 (0.775 - 0.997)	***0.038***	*0.071*
Mothers of females	305 (50)	0.73 (0.44)	0.55 (0.5)	0.721 (0.588 - 0.884)	***0.001***	***0.002***
Mothers of males	310 (50)	0.68 (0.47)	0.70 (0.46)	0.997 (0.848 - 1.172)	*0.972*	*0.973*
No. Health Conditions, child 18y						
Overall sample	616 (100)	1.75 (2.01)	0.17 (1.96)	-0.131 (-0.464 - 0.202) ‡	*0.442*	*0.441*
Mothers of females	306 (50)	1.75 (1.82)	1.24 (1.64)	-0.553 (-0.966 - -0.14) ‡	***0.009***	***0.016***
Mothers of males	310 (50)	1.74 (2.20)	2.07 (2.14)	0.215 (-0.296 - 0.726) ‡	*0.409*	*0.835*

Note. The columns present the following information: (i) outcome analyzed; (ii) sample size (N); (iii) mean of the control group; (iv) mean of the treatment group; (v) least squares mean difference (LSMD) for continuous outcomes (superscript ‡), adjusted relative risk for binary variables (estimated through a logistic regression), and 95% confidence interval in parenthesis; (vi) two-sided asymptotic p-value for the null hypothesis that the treatment effect is zero; (vii) two-sided multiple hypothesis testing (MHT) p-value using the step-down methodology of Romano and Wolf (2005).[17]

© 2024 Conti G et al. *JAMA Network Open.*

eTable 7. Mediation Analysis, Mothers of Girls Hypertension and Obesity of Girls at Age 18 Years

Mediators	Mothers of Females Stage 1 Hypertension at child age 18y	
	**Coefficient**	**% explained**
Model 1: one mediator		
Mother Stage 1 Hypertension at child age 12	0.289*** (0.051)	27.3***
Model 2: two mediators		
Mother Stage 1 Hypertension at child age 12	0.267*** (0.050)	Both mediators: 30.3***
+ Mother Obese at child age 12	0.178*** (0.053)	
	**Female Offspring Obesity at age 18y**	
	**Coefficient**	**% explained**
Model 1: one mediator		
Child Obese at age 12	0.619*** (0.051)	75.4***
Model 2: two mediators		
Child Obese at age 12	0.608*** (0.053)	Both mediators: 82.6***
+ Child Overweight at age 2	0.069 (0.069)	
	**Female Offspring Severe Obesity at age 18y**	
	**Coefficient**	**% explained**
Model 1: one mediator		
Child Severely Obese at age 12	0.556*** (0.053)	57.1**
Model 2: two mediators		
Child Severely Obese at age 12	0.522*** (0.055)	Both mediators: 65.2**
+ Child Overweight at age 2	0.103* (0.061)	

Note. This table presents the results of the mediation analysis for three primary outcomes of the NFP intervention. For each outcome within each panel, two models are presented: model 1 has one mediator only, model 2 has two mediators; the mediators are shown in the first column. For each model, two columns of results are presented. The first column (labelled "coefficient") presents the coefficient of that mediator in the mediation model (standard errors in parenthesis); the second column (labelled "% explained") presents the proportion of the treatment effect that the single mediator (for model 1) or both mediators together (for model 2) explain. The mediation analysis is performed using the method developed by Gelbach (2016) [20]. In addition to the treatment indicator and the mediators, the mediation model includes the variables used in the randomization protocol, and the covariates selected by the post double selection lasso for that outcome. ***:p<0.01; **:p<0.05; *:p<0.10.

© 2024 Conti G et al. *JAMA Network Open.*

eTable 8. Intergenerational Correlations in Health

	Outcome	
	Female Offspring Stage 1 Hypertension at age 18y	
	**Control Group**	**Treated Group**
Mothers of Females Stage 1 Hypertension at age 18y	0.117 (0.095)	0.011 (0.114)
	Female Offspring Stage 2 Hypertension at age 18y	
	**Control Group**	**Treated Group**
Mothers of Females Stage 2 Hypertension at age 18y	0.113**+ (0.047)	-0.075 (0.070)
	Female Offspring Obesity at age 18y	
	**Control Group**	**Treated Group**
Mothers of Females Obesity at age 18y	0.148** (0.068)	0.151* (0.088)
	Female Offspring Severe Obesity at age 18y	
	**Control Group**	**Treated Group**
Mothers of Females Severe Obesity at age 18y	0.158*** (0.055)	0.126* (0.073)

Note. This table presents intergenerational (IG) correlations estimated via Ordinary Least Squares between mother and offspring stage-1 and -2 hypertension at child age 18 (first and second panel) and mother and offspring obesity and severe obesity at child age 18 (third and fourth panel). For each of the four outcomes, the child outcome is the dependent variable of the regression, and the corresponding maternal outcome is the independent variable; each cell displays results from a separate regression of a child outcome on the corresponding maternal outcome, separately for the control group (second column) and the treated group (third column). ***:p<0.01; **:p<0.05; *:p<0.10. +: the difference in the IG correlation coefficients between the treated and the control group is significant at 5%.

© 2024 Conti G et al. *JAMA Network Open.*

eTable 9. NFP Postintervention Treatment Effects on Offspring Obesity and Severe Obesity and Maternal Stage 1 and Stage 2 Hypertension—Inverse Probability Weighting and Tests for Significance of Sex Differences

	N	CM	TM	ARR	Asy. p-val	IPW ARR	N	CM	TM	ARR	Asy. p-val	IPW ARR	Sex Diff. in CM *p-value*	Sex Diff. in TE (OR) *p-value*
Child	***Girls***						***Boys***							
Obese at 12y	286	0.32	0.15	0.493	***0.002***	0.517***	288	0.21	0.30	1.454	*0.091*	1.332	***0.018***	***0.020***
Obese at 18y	307	0.31	0.22	0.698	*0.075*	0.702*	297	0.18	0.18	1.030	*0.911*	1.021	***0.002***	*0.562*
Obese at 12y & 18y	269	0.25	0.11	0.449	***0.003***	0.499**	269	0.14	0.16	1.248	*0.489*	1.272	***0.003***	*0.327*
Severely Obese at 12y	287	0.18	0.07	0.396	***0.007***	0.533***	288	0.10	0.16	1.510	*0.229*	0.925	***0.027***	*0.068*
Severely Obese at 18y	307	0.18	0.11	0.561	***0.046***	0.585**	297	0.12	0.11	0.928	*0.831*	0.925	*0.065*	*0.898*
Severely Obese at 12y & 18y	269	0.12	0.02	0.185	***0.000***	0.198***	269	0.06	0.09	1.517	*0.672*	1.536	***0.036***	*0.057*
Mother	***Mothers of Girls***						***Mothers of Boys***							
Stage 1 HTN, child 12y	253	0.61	0.43	0.695	***0.006***	0.737**	253	0.56	0.52	0.914	*0.460*	0.881	*0.406*	*0.251*
Stage 1 HTN, child 18y	293	0.81	0.67	0.793	***0.003***	0.798***	298	0.82	0.83	1.035	*0.516*	1.001	*0.818*	***0.002***
Stage 1 HTN, child 12y & 18y	238	0.57	0.36	0.613	***0.001***	0.629***	247	0.52	0.49	0.946	*0.672*	0.900	*0.408*	***0.004***
Stage 2 HTN, child 12y	252	0.33	0.10	0.313	***0.000***	0.298***	254	0.27	0.27	0.935	*0.728*	0.945	*0.254*	***0.008***
Stage 2 HTN, child 18y	293	0.51	0.46	0.864	*0.268*	0.852	298	0.50	0.56	1.160	*0.180*	1.108	*0.805*	*0.051*
Stage 2 HTN, child 12y & 18y	232	0.27	0.05	0.217	***0.000***	0.201***	247	0.20	0.21	1.076	*0.782*	1.077	*0.145*	***0.006***

Note. The table is composed of two panels: the upper panel presents results for the offspring sample, and the lower panel presents results for their mothers. The columns present the following information: (i) outcome analyzed; (ii,viii) sample size (N); (iii,ix) mean of the control group (CM); (iv-x) mean of the treatment group (TM); (v-xi) adjusted relative risk (ARR) estimated through a logistic regression; (vi-xii) two-sided asymptotic p-value for the null hypothesis that the treatment effect is zero (Asy. p-val); (vii,xiii) adjusted relative risk which accounts for attrition using Inverse Probability Weighting (IPW ARR); (xiv) p-value for the difference in means between the male and female sample in the control group; (xv) p-value for the sex interaction term of the logit log odds ratio (OR) of the treatment effect (i.e., the parameter of the logistic regression on the treatment binary variable interacted with the male binary variable). We control for baseline covariates selected using the post-double selection lasso procedure, from a set of 130 covariates; variables used in the randomization procedure (race, age, gestational age at intake, head of household employed and region of residence, see top panel of Table S2A) are partialled out. The covariates included in the logit model for the probability of retention are selected from the strata variables and the baseline variables in Tables S2A-B. P-values less than or equal to 0.05 are bolded. ***:p<0.01; **:p<0.05; *:p<0.10. Offspring obesity at ages 12 and 18 is defined as a standardized BMI at or above the 95th percentile of the BMI-for-age distribution (2000 CDC growth charts).[10] Offspring severe obesity at ages 12 and 18 is defined as a standardized BMI at or above the 99th percentile of the BMI-for-age distribution (2000 CDC growth charts).[10] Mother stage 1 hypertension is defined as systolic blood pressure ≥ 130 or diastolic blood pressure ≥ 80. Mother stage 2 hypertension is defined as systolic blood pressure ≥ 140 or diastolic blood pressure ≥ 90.[12]

© 2024 Conti G et al. *JAMA Network Open.*

eTable 10. NFP Postintervention Treatment Effects on Child Obesity and Mother Hypertension—Robustness to Inclusion of Covariates Unbalanced at Baseline

	N	CM	TM	ARR (Lasso)	Asy. p-value (Lasso)	ARR (Imbal.)	Asy. p-value (Imbal.)
Child	***Girls***						
Obese at 12y	286	0.32	0.15	0.493	***0.002***	0.543	***0.007***
Obese at 18y	307	0.31	0.22	0.698	***0.075***	0.770	*0.216*
Obese at 12y & 18y	269	0.25	0.11	0.449	***0.003***	0.508	***0.013***
Severely Obese at 12y	287	0.18	0.07	0.396	***0.007***	0.429	***0.009***
Severely Obese at 18y	307	0.18	0.11	0.561	***0.046***	0.652	*0.102*
Severely Obese at 12y & 18y	269	0.12	0.02	0.185	***0.000***	0.236	***0.001***
Mother	***Mothers of Girls***						
Stage 1 HTN, child 12y	253	0.61	0.43	0.695	***0.006***	0.704	***0.010***
Stage 1 HTN, child 18y	293	0.81	0.67	0.793	***0.003***	0.771	***0.001***
Stage 1 HTN, child 12y & 18y	238	0.57	0.36	0.613	***0.001***	0.593	***0.001***
Stage 2 HTN, child 12y	252	0.33	0.10	0.313	***0.000***	0.283	***0.000***
Stage 2 HTN, child 18y	293	0.51	0.46	0.864	*0.268*	0.875	*0.319*
Stage 2 HTN, child 12y & 18y	232	0.27	0.05	0.217	***0.000***	0.184	***0.000***

Note. The table is composed of two panels: the upper panel presents results for the offspring sample, and the lower panel presents results for their mothers. The columns present the following information: (i) outcome analyzed; (ii) sample size (N); (iii) mean of the control group (CM); (iv) mean of the treatment group (TM); (v) adjusted relative risk (ARR) using the post-double selection lasso to select the baseline covariates; (vi) related two-sided asymptotic p-value for the null hypothesis that the treatment effect is zero (Asy. p-value); (vii) adjusted relative risk including the imbalanced baseline covariates (from Table S2A and S2B); (viii) related two-sided asymptotic p-value for the null hypothesis that the treatment effect is zero. P-values less than or equal to 0.05 are bolded. Offspring obesity at ages 12 and 18 is defined as a standardized BMI at or above the 95th percentile of the BMI-for-age distribution (2000 CDC growth charts) [10]. Offspring severe obesity at ages 12 and 18 is defined as a standardized BMI at or above the 99th percentile of the BMI-for-age distribution (2000 CDC growth charts)[10]. Mother stage 1 hypertension is defined as systolic blood pressure ≥ 130 or diastolic blood pressure ≥ 80. Mother stage 2 hypertension is defined as systolic blood pressure ≥ 140 or diastolic blood pressure ≥ 90.[12]

© 2024 Conti G et al. *JAMA Network Open.*

eTable 11. Postintervention NFP Effects on Offspring Obesity and Severe Obesity by Offspring Gender Robustness to Exclusion of Self-Reported BMI Values

Outcome	N (%)	Control Mean (SD)	Treatment Mean (SD)	Adjusted Relative Risk (95% CI)	*p-value*	MHT *p-value*
Obese at 12y						
Overall sample	562	0.27 (0.44)	0.23 (0.42)	0.843 (0.623, 1.140)	*0.251*	*0.377*
Female	283 (50.5)	0.32 (0.47)	0.15 (0.36)	0.488 (0.289, 0.824)	***0.001***	***0.005***
Male	277 (49.5)	0.22 (0.41)	0.30 (0.46)	1.349 (0.883, 2.061)	*0.186*	*0.306*
Obese at 18y						
Overall sample	578	0.25 (0.43)	0.21 (0.41)	0.832 (0.609, 1.137)	*0.230*	*0.377*
Female	292 (50.6)	0.32 (0.47)	0.22 (0.42)	0.707 (0.456, 1.096)	*0.093*	*0.107*
Male	285 (49.4)	0.18 (0.38)	0.20 (0.40)	1.134 (0.679, 1.895)	*0.640*	*0.684*
Obese at 12y & 18y						
Overall sample	517	0.20 (0.39)	0.15 (0.35)	0.751 (0.488, 1.154)	*0.163*	*0.345*
Female	262 (50.9)	0.26 (0.44)	0.11 (0.32)	0.453 (0.237, 0.864)	***0.004***	***0.009***
Male	253 (49.1)	0.14 (0.34)	0.17 (0.38)	1.215 (0.656, 2.253)	*0.550*	*0.684*
Severely Obese at 12y						
Overall sample	562	0.14 (0.35)	0.11 (0.32)	0.778 (0.481, 1.259)	*0.282*	*0.463*
Female	284 (50.6)	0.18 (0.39)	0.07 (0.26)	0.390 (0.166, 0.920)	***0.006***	***0.027***
Male	277 (49.4)	0.11 (0.31)	0.15 (0.36)	1.288 (0.665, 2.495)	*0.471*	*0.738*
Severely Obese at 18y						
Overall sample	575	0.15 (0.36)	0.12 (0.33)	0.804 (0.505, 1.282)	*0.337*	*0.463*
Female	291 (50.5)	0.18 (0.38)	0.11 (0.32)	0.607 (0.316, 1.166)	*0.092*	*0.091*
Male	285 (49.5)	0.12 (0.32)	0.12 (0.32)	1.015 (0.504, 2.043)	*0.967*	*0.968*
Severely Obese at 12y & 18y						
Overall sample	516	0.09 (0.29)	0.06 (0.23)	0.597 (0.291, 1.223)	*0.192*	*0.281*
Female	263 (52.4)	0.13 (0.33)	0.03 (0.16)	0.187 (0.046, 0.752)	***0.001***	***0.020***
Male	239 (47.6)	0.06 (0.24)	0.09 (0.29)	1.376 (0.534, 3.544)	*0.429*	*0.738*

Note. The columns present the following information: (i) outcome analyzed; (ii) sample size (N); (iii) mean of the control group; (iv) mean of the treatment group; (v) adjusted relative risk estimated through a logistic regression with 95% confidence interval in parenthesis (95% CI); (vi) two-sided asymptotic p-value for the null hypothesis that the treatment effect is zero; (vii) two-sided multiple hypothesis testing (MHT) p-value using the step-down methodology of Romano and Wolf (2005).[17] Offspring obesity at ages 12 and 18 is defined as a standardized BMI at or above the 95th percentile of the BMI-for-age distribution (2000 CDC growth charts).[10] Offspring severe obesity at ages 12 and 18 is defined as a standardized BMI at or above the 99th percentile of the BMI-for-age distribution (2000 CDC growth charts).[10]

© 2024 Conti G et al. *JAMA Network Open.*

eTable 12. Postintervention NFP Effects on Maternal Obesity and Severe Obesity by Offspring Gender—Robustness to Exclusion of Self-Reported BMI Values

Outcome	N (%)	Control Mean (SD)	Treatment Mean (SD)	Adjusted Relative Risk (95% CI)	*p-value*	MHT *p-value*
Obese at 12y						
Overall sample	497	0.54 (0.50)	0.57 (0.50)	0.993 (0.861, 1.144)	*0.917*	*0.990*
Mothers of females	249 (50.3)	0.60 (0.49)	0.55 (0.50)	1.012 (0.837, 1.224)	*0.900*	*0.984*
Mothers of males	246 (49.7)	0.48 (0.50)	0.58 (0.50)	0.985 (0.793, 1.223)	*0.889*	*0.949*
Obese at 18y						
Overall sample	573	0.60 (0.49)	0.58 (0.49)	0.954 (0.835, 1.089)	*0.481*	*0.747*
Mothers of females	283 (49.4)	0.62 (0.49)	0.55 (0.50)	0.945 (0.777, 1.150)	*0.569*	*0.837*
Mothers of males	290 (50.6)	0.58 (0.49)	0.61 (0.49)	0.940 (0.782, 1.129)	*0.501*	*0.793*
Obese at 12y & 18y						
Overall sample	462	0.49 (0.50)	0.51 (0.50)	0.995 (0.840, 1.179)	*0.953*	*0.990*
Mothers of females	231 (50.1)	0.54 (0.50)	0.49 (0.50)	0.986 (0.766, 1.269)	*0.912*	*0.988*
Mothers of males	230 (49.9)	0.43 (0.50)	0.53 (0.50)	0.974 (0.769, 1.233)	*0.825*	*0.949*
Severely Obese at 12y						
Overall sample	497	0.32 (0.47)	0.36 (0.48)	1.023 (0.827, 1.266)	*0.835*	*0.964*
Mothers of females	251 (50.5)	0.34 (0.47)	0.36 (0.48)	1.157 (0.855, 1.566)	*0.354*	*0.595*
Mothers of males	246 (49.5)	0.30 (0.46)	0.36 (0.48)	0.857 (0.629, 1.168)	*0.314*	*0.565*
Severely Obese at 18y						
Overall sample	573	0.34 (0.47)	0.36 (0.48)	0.999 (0.821, 1.214)	*0.988*	*0.986*
Mothers of females	282 (49.3)	0.38 (0.49)	0.34 (0.48)	0.921 (0.682, 1.243)	*0.583*	*0.760*
Mothers of males	290 (50.7)	0.30 (0.46)	0.38 (0.49)	1.022 (0.791, 1.321)	*0.869*	*0.956*
Severely Obese at 12y & 18y						
Overall sample	462	0.26 (0.44)	0.30 (0.46)	1.042 (0.808, 1.344)	*0.754*	*0.959*
Mothers of females	230 (49.9)	0.29 (0.46)	0.28 (0.45)	1.071 (0.733, 1.564)	*0.728*	*0.760*
Mothers of males	231(50.1)	0.23 (0.42)	0.32 (0.47)	0.963 (0.684, 1.355)	*0.828*	*0.956*

Note. The columns present the following information: (i) outcome analyzed; (ii) sample size (N); (iii) mean of the control group; (iv) mean of the treatment group; (v) adjusted relative risk estimated through a logistic regression with 95% confidence interval in parenthesis (95% CI); (vi) two-sided asymptotic p-value for the null hypothesis that the treatment effect is zero; (vii) two-sided multiple hypothesis testing (MHT) p-value using the step-down methodology of Romano and Wolf (2005).[17]. Offspring obesity at ages 12 and 18 is defined as a standardized BMI at or above the 95th percentile of the BMI-for-age distribution (2000 CDC growth charts).[10] Offspring severe obesity at ages 12 and 18 is defined as a standardized BMI at or above the 99th percentile of the BMI-for-age distribution (2000 CDC growth charts).[10]

© 2024 Conti G et al. *JAMA Network Open.*

eTable 13. NFP Treatment Effects on Weight-for-Age and Height-for-Age in the First Year of Life

	**Weight**		**Height**	
	**Girls**	**Boys**	**Girls**	**Boys**
Treatment	0.147	0.328**	-0.196	0.014
	(0.169)	(0.163)	(0.214)	(0.193)
Age (days)	0.714***	0.593***	-0.072	-0.133**
	(0.037)	(0.040)	(0.062)	(0.064)
Treatment*Age	-0.270***	-0.087	-0.069	0.059
	(0.072)	(0.072)	(0.117)	(0.117)
Age squared	-0.099***	-0.085***	0.029***	0.032***
	(0.006)	(0.006)	(0.011)	(0.011)
Treatment*Age squared	0.042***	0.011	0.013	-0.001
	(0.011)	(0.012)	(0.021)	(0.021)
Age cubed	0.004***	0.004***	-0.002***	-0.002***
	(0.000)	(0.000)	(0.001)	(0.001)
Treatment*Age cubed	-0.002***	-0.000	-0.001	-0.000
	(0.001)	(0.001)	(0.001)	(0.001)
No. of children	329	336	329	334
No. of observations	2,068	2,104	1,601	1,593

Note: This table presents estimates (coefficients and standard errors in parentheses) from a growth curve model with a cubic in age interacted with the treatment status, child-specific random intercept and quadratic random slopes and unrestricted covariance between the two random effects. Weight and height have been measured during the developmental screening in the first year of life and have been standardized using the 2000 CDC growth charts.[10] All the models control for the variables used in the randomization protocol. ***p<0.01, **p<0.05, *p<0.001.

eTable 14. Self-Reported Race and Ethnicity by Treatment Status

	Control	Treatment	Total
Aleut, Eskimo, or American Indian	6 (1.17%)	1 (0.44%)	7 (0.94%)
Asian or Pacific Islander	1 (0.19%)	0 (0.00%)	1 (0.13%)
Black	474 (92.22%)	205 (89.91%)	679 (91.51%)
White	33 (6.42%)	21 (9.21%)	54 (7.28%)
Other	0 (0.00%)	1 (0.44%)	1 (0.13%)
Total	514 (100%)	228 (100%)	742 (100%)

Note: This table displays the frequency distribution of self-reported race and ethnicity among trial participants, categorized by treatment status. Relative frequencies by treatment status are presented in parentheses.

© 2024 Conti G et al. *JAMA Network Open.*

eFigure 1. NFP Treatment Effects on Weight-for-Age in the First Year of Life

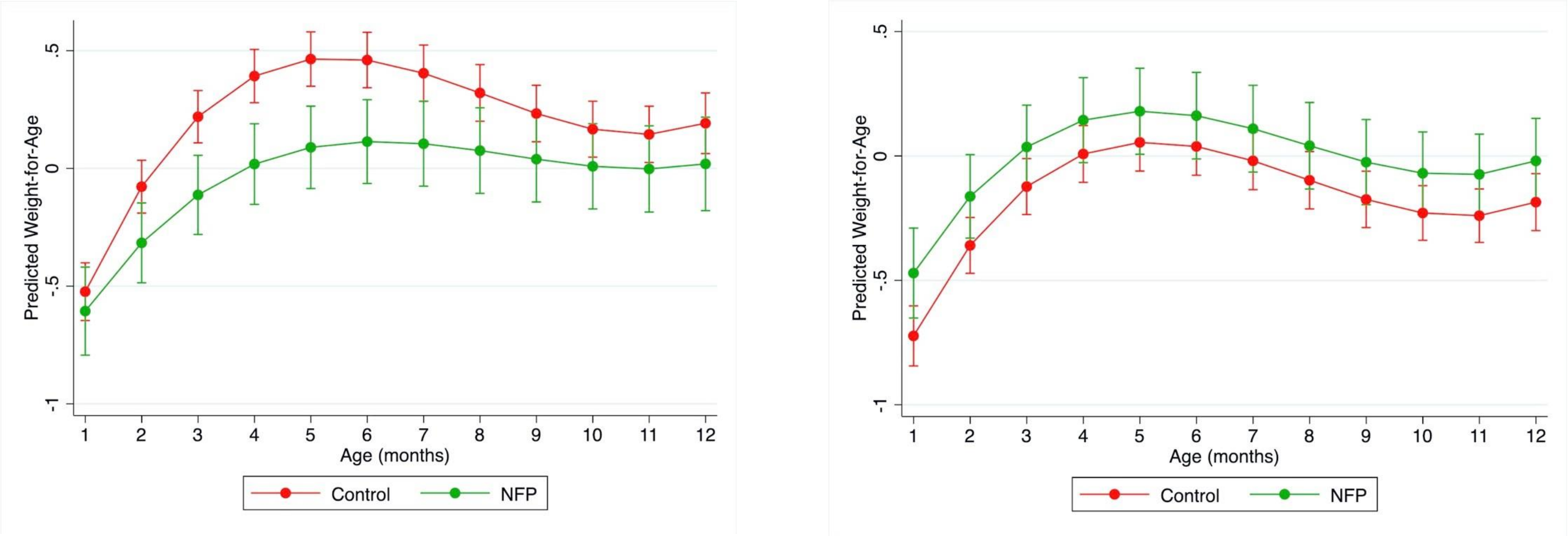

(a) Girls ***(b) Boys***

Note. The figures plot predicted weight-for-age in the first year of life for treatment (green line) and control (red line) groups for girls (a) and boys (b), based on a growth model with a cubic in age interacted with treatment status, a random intercept at the child level and random slopes for age and age squared, and unrestricted covariance between the random effects. We also control for the variables used in the randomization protocol (race, age, gestational age at intake, head of household employed and region of residence, see top panel of Table S2A). Weight was measured during the developmental screenings that both treatment and control group received; z-scores have been computed using the 2000 CDC Growth Reference Charts[10].

© 2024 Conti G et al. *JAMA Network Open.*

eFigure 2. NFP Treatment Effects on Height-for-Age in the First Year of Life

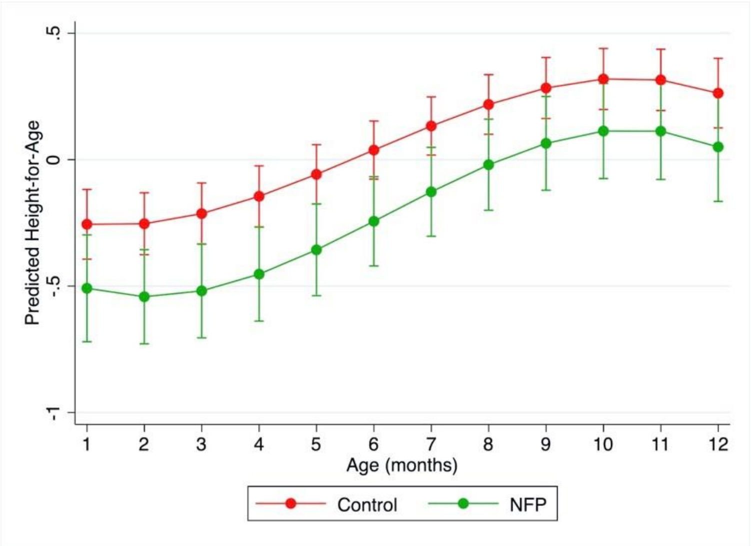

(a) Girls

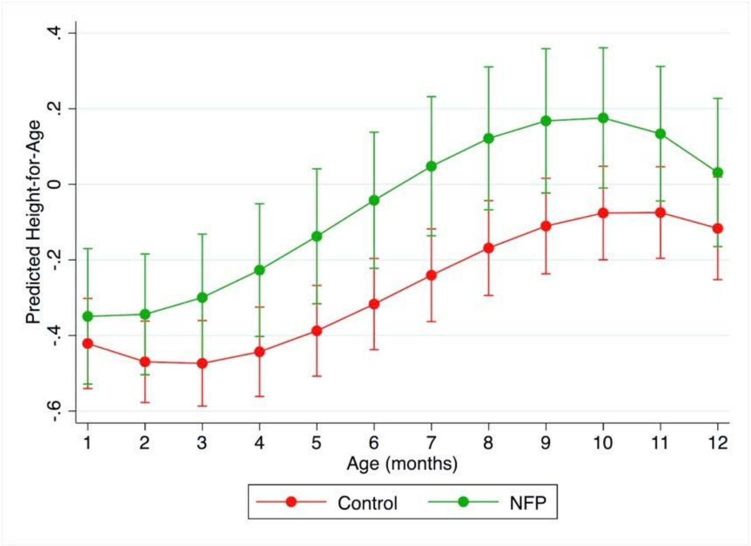

(b) Boys

Note. The figures plot predicted height-for-age in the first year of life for girls (a) and boys (b), based on a growth model with a cubic in age interacted with treatment status, a random intercept at the child level and random slopes for age and age squared, and unrestricted covariance between the random effects. We also control for the variables used in the randomization protocol (race, age, gestational age at intake, head of household employed and region of residence, see top panel of Table S2A). Height was measured during the developmental screenings that both treatment and control group received; *z*-scores have been computed using the 2000 CDC Growth Reference Charts[10].

© 2024 Conti G et al. *JAMA Network Open.*

eReferences

1. Olds, D.L. (2002). Prenatal and infancy home visiting by nurses: from randomized trials to community replication. *Prevention Science: the official journal of the Society for Prevention Research*, 3(3), 153–172.
2. Swain, J.E., Kim, P., Spicer, J., Ho, S.S., DaytSton, C.J., Elmadih, A., & Abel, K.M. (2014). Approaching the biology of human parental attachment: brain imaging, oxytocin and coordinatedassessments of mothers and fathers. *Brain research*, 1580, 78–101.
3. Watson, J. (1979). *Nursing: The philosophy and science of caring*. Little.
4. Buka, S. (2005). Developmental Epidemiology: The Role of Developmental Psychology for Public Health in the 21st Century. In D. Pillemer & S. White (Eds.), *Developmental Psychology and Social Change: Research, History and Policy* (Cambridge Studies in Social and Emotional Development, pp. 173-202). Cambridge: Cambridge University Press. doi:10.1017/CBO9780511610400.009
5. Bowlby, J. (1969). *Attachment and loss, Vol. 1: Attachment*. Basic Books.
6. Bronfenbrenner, U. (1979). *The ecology of human development: Experiments by nature and design*.Harvard University Press.
7. Bandura, A. (1977). Self-efficacy: Toward a unifying theory of behavioral change. *Psychological Review,84*(2), 191-215.
8. Ainsworth, M.D., Blehar, M.C., Waters, E., & Wall, S. (1978). *Patterns of attachment: A Psychological study of the strange situation*. Erlbaum.
9. Olds, D.L., Henderson Jr, C.R., Tatelbaum, R., & Chamberlin, R. (1988). Improving the life-course development of socially disadvantaged mothers: a randomized trial of nurse home visitation. *American journal of public health*, *78*(11), 1436-1445.
10. Kuczmarski, R. J. (2002). 2000 CDC growth charts for the United States: methods and development (No. 246). Department of Health and Human Services, Centers for Disease Control and Prevention, National Center for Health Statistics.
11. National High Blood Pressure Education Program Working Group on High Blood Pressure in Children and Adolescents. "The fourth report on the diagnosis, evaluation, and treatment of high blood pressure in children and adolescents." *Pediatrics* 114.Supplement_2 (2004): 555-576 (p.37 and Table B1)
12. The Seventh Report of the Joint National Committee on Prevention, Detection Evaluation and Treatment of High Blood Pressure. U.S. Department OF Health and Human Services https://www.nhlbi.nih.gov/files/docs/guidelines/express.pdf
13. IOM. *Weight Gain During Pregnancy: Reexamining the Guidelines*. Institute of Medicine (US) and National Research Council (US) and Committee to Reexamine IOM Pregnancy Weight Guidelines; 2009.
14. Achenbach TM, Edelbrock C, Howell CT. Empirically based assessment of the behavioral/emotional problems of 2- and 3- year-old children. *Journal of Abnormal Child Psychology*. 1987;15(4):629-650. doi:10.1007/bf00917246
15. Belloni, A., Chernozhukov, V., & Hansen, C. (2014). Inference on treatment effects after selection among high-dimensional controls. *The Review of Economic Studies*, 81(2), 608- 650.
16. Duflo, E. (2018). Machinistas meet randomistas: useful ML tools for empirical researchers. Master lecture *NBER* Summer Institute.
17. Romano, J. P., & Wolf, M. (2005). Exact and approximate stepdown methods for multiple hypothesis testing. *Journal of the American Statistical Association*, 100(469), 94-108.
18. Campbell F, Conti G, Heckman JJ, et al. Early childhood investments substantially boost adult health. *Science*. 2014:343(6148):1478-85.

© 2024 Conti G et al. *JAMA Network Open.*

19. Koenker, R. 2005. Quantile Regression. Cambridge University Press: New York.
20. Gelbach, J. B. (2016). When do covariates matter? And which ones, and how much?
21. *Journal of Labor Economics*, 34(2), 509-543.
22. Kitzman H, Olds DL, Henderson CR Jr., et al. Effect of prenatal and infancy home visitation by nurses on pregnancy outcomes, childhood injuries, and repeated childbearing. A randomized controlled trial. *JAMA*. 1997;278(8):644-52

© 2024 Conti G et al. *JAMA Network Open.*
