# Supplementary material for: Early Home Visits and Health Outcomes in Low-Income Mothers and Offspring: 18-Year Follow-Up of a Randomized Clinical Trial
Source: JAMA Netw Open. 2024 Jan 18;7(1):e2351752. doi: 10.1001/jamanetworkopen.2023.51752 (PMC10797459; doi:10.1001/jamanetworkopen.2023.51752)
Supplement: Supplement 3. — Data Sharing Statement [file jamanetwopen-e2351752-s003.pdf]

Data Sharing Statement

Conti. Early Home Visits and Health Outcomes in Low-Income Mothers and Offspring. *JAMA Netw Open*. Published January 18, 2024. doi:10.1001/jamanetworkopen.2023.51752

Data
Data available: Yes
Data types: Deidentified participant data, Data dictionary
How to access data: The data will be made available on publication to researchers who provide a methodologically sound proposal for their use. Please contact David Olds at david.olds@cuanschutz.edu for additional details.
When available: With publication

Supporting Documents
Document types: Statistical/analytic code
How to access documents: The data will be made available on publication to researchers who provide a methodologically sound proposal for their use. Please contact David Olds at david.olds@cuanschutz.edu for additional details.
When available: With publication

Additional Information
Who can access the data: The data will be made available on publication to researchers who provide a methodologically sound proposal for their use. Please contact David Olds at david.olds@cuanschutz.edu for additional details.
Types of analyses: The data will be made available on publication to researchers who provide a methodologically sound proposal for their use. Please contact David Olds at david.olds@cuanschutz.edu for additional details.
Mechanisms of data availability: The data will be made available on publication to researchers who provide a methodologically sound proposal for their use. Please contact David Olds at david.olds@cuanschutz.edu for additional details.
